# *p*-Pyridinyl oxime carbamates: synthesis, DNA binding, DNA photocleaving activity and theoretical photodegradation studies

**DOI:** 10.3762/bjoc.16.33

**Published:** 2020-03-09

**Authors:** Panagiotis S Gritzapis, Panayiotis C Varras, Nikolaos-Panagiotis Andreou, Katerina R Katsani, Konstantinos Dafnopoulos, George Psomas, Zisis V Peitsinis, Alexandros E Koumbis, Konstantina C Fylaktakidou

**Affiliations:** 1 Laboratory of Organic, Bioorganic and Natural Product Chemistry, Molecular Biology and Genetics Department, Democritus University of Thrace, University Campus, Dragana, 68100, Alexandroupolis, Greece; 2 Laboratory of Biochemistry and Molecular Virology, Molecular Biology and Genetics Department, Democritus University of Thrace, University Campus, Dragana, 68100, Alexandroupolis, Greece; 3 Laboratory of Inorganic Chemistry, Chemistry Department, Aristotle University of Thessaloniki, 54124, Thessaloniki, Greece; 4 Laboratory of Organic Chemistry, Chemistry Department, Aristotle University of Thessaloniki, 54124, Thessaloniki, Greece

**Keywords:** DNA binding, DNA photocleavage, N–O homolysis, oxime carbamates, photocleavage agents

## Abstract

A number of *p*-pyridinyl oxime carbamate derivatives were prepared upon the reaction of the corresponding oximes with isocyanates. These novel compounds reacted photochemically in the presence of supercoiled plasmid DNA. Structure–activity relationship (SAR) studies revealed that the substituent on the imine group was not affecting the extend of the DNA damage, whereas the substituent of the carbamate group was critical, with the halogenated derivatives to be able to cause extensive single and double stranded DNA cleavages, acting as “synthetic nucleases”, independently of oxygen and pH. Calf thymus–DNA affinity studies showed a good-to-excellent affinity of selected both active and non-active derivatives. Preliminary theoretical studies were performed, in an effort to explain the reasons why some derivatives cause photocleavage and some others not, which were experimentally verified using triplet state activators and quenchers. These theoretical studies seem to allow the prediction of the activity of derivatives able to pass intersystem crossing to their triplet energy state and thus create radicals able to damage DNA. With this study, it is shown that oxime carbamate derivatives have the potential to act as novel effective photobase generating DNA-photocleavers, and are proposed as new leads for “on demand” biotechnological applications in drug discovery and medicine.

## Introduction

Small organic molecules able to bind DNA provide promises for anticancer activity due to alteration of the structure and function of the genetic material. Amongst a plethora of such binders [1–8], various oxime derivatives were found to show affinity towards DNA [9–12], whereas others were found to cleave DNA as metal-free artificial nucleases [13–14].

The interaction of molecules with DNA and their affinity towards this macromolecule also plays a key role in photosensitization techniques. Light activated oxidative DNA cleaving agents are called “DNA photocleavers”. These compounds are able to absorb light and to be selectively excited. A variety of reaction mechanisms are initiated, which, aiming to the photocleaver, may lead to DNA damage. A requirement for nucleic acid’s and most protein’s “transparency” is irradiating at wavelengths longer than 310 nm [15]. “Transparency” means lack of damage due to irradiation itself and action via its combination with the photosensitizer. It is worth mentioning that, mainly in dermatology, even UVB irradiation is considered of therapeutic use [16–18]. Besides the anticancer activities of photosensitizers [19–21], “post-antibiotic era” is experimenting with photosensitizers as alternative therapeutics for the fight against multiresistant bacteria both for medicinal [22–25] and environmental purposes [26–27].

Several organic compounds were found to be “DNA photocleavers”, exhibiting their action at 312 nm, like [1,2,4]triazolo[4,3-*a*]quinoxaline [28] and quinoxalin-4(5*H*)-one [29] derivatives, various enediyne [30–32], proflavine [33], *N*-nitroso carboxamide [34], naphazoline [35] and triazole [36] derivatives, azido carbonyl compounds [37] and *N*,*O*-diacyl-4-benzoyl-*N*-phenylhydroxylamines [38].

**Figure 1:**
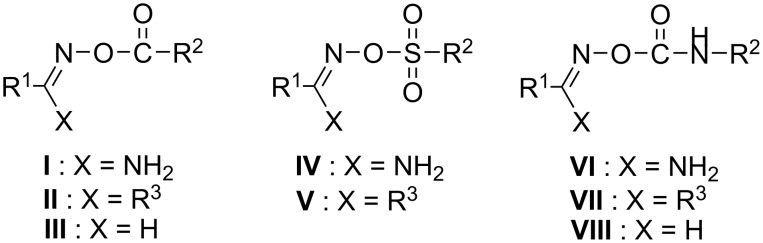
General structures of oxime derivatives with possible DNA photocleavage ability. Left: Oxime carboxylates: *O*-acyl amidoximes (**I**), *O*-acyl ketoximes (**II**), *O*-acyl aldoximes (**III**). Centre: Oxime sulfonates: *O*-sulfonyl amidoximes (**IV**), *O*-sulfonyl ketoximes (**V**). Right: Oxime carbamates: *O*-carbamoyl amidoximes (**VI**), *O*-carbamoyl ketoximes (**VII**) and *O*-carbamoyl aldoximes (**VIII**).

*O*-Acyl amidoximes, ketoximes and aldoximes (**I**, **II** and **III**, respectively, Figure 1) are also recognized as DNA “photocleavage” agents owing their action to the homolysis of their vulnerable N–O bond, at 312 nm [9,39–43] or 365 nm [44–45] yielding photogenerated carbonyloxyl radicals (CRs), which are able to cause oxidative DNA damage. We have recently reported the DNA photocleavage from sulfonylamidoximes and ethanone oximes (**IV** and **V**, Figure 1), which were found to attack DNA via sulfonyloxyl radicals (SRs) [10–11]. All the above radical species exhibit photoreactivity towards DNA.

Those oxime derivatives are considered photoacid generators (PAGs) since one of the residual fragments produced by the N–O homolytic cleavage is a carboxylic or a sulfonic acid, generated upon hydrogen abstraction from the corresponding oxygen centred radicals, carbonyloxyl and sulfonyloxyl, respectively. Some are used in the field of photoresists for semiconductor fabrication [46–47]. Oxime carboxylic or sulfonic esters also produce nitrogen-centered iminyl (R_2_C=N^∙^) or amidinyl [R(NH_2_)C=N^∙^] radicals, nevertheless, their damage towards DNA was not well recognized and, thus, DNA photocleavage aimed by nitrogen-centered stable organic radicals has been less investigated. Reports involve arylaminyl radicals (ArNH^∙^) formed from arylhydrazones through the photoinduced cleavage of N–N single bonds (along with an iminyl radical) [48] or from benzotriazole derivatives after also a N–N single bond cleavage and nitrogen elimination [36]. It has been reported that amine-centred radicals are also produced from 2-(1-naphthylmethyl)imidazoline [35], whereas acylaminyl radicals [R(COR)N^∙^] are formed from the photocleavage of the N–O bond of *N*,*O*-diacyl-*N*-phenylhydroxylamines, along with a carbonyloxyl radical [38].

The photochemistry of *O*-carbamoyl oximes (or oxime carbamates) is well studied. These compounds are categorized as highly photoreactive photobase generators (PBGs), providing amines upon rapid decarboxylation of the initially formed carbamoyloxyl radicals (R_2_NCOO^∙^) [49–53]. To the best of our knowledge oxime carbamates have never been photocleaved in the presence of DNA. Based on our interest in the chemistry and biology of the oxime functionality [54–57], as well as in their DNA photolytic interaction upon UV irradiation [9–11,43] we have decided to investigate the behaviour of carefully designed *O*-carbamoyl derivatives of *p*-pyridine amidoxime, ethanone oxime and aldoxime (**VI**, **VII**, **VIII**, R_1_ = *p*-pyridyl, Figure 1) as DNA photosensitizers. Based on our previous experimental results with *o*-, *m*- and *p*-pyridine oximes as carriers of the carboxylic [9] and sulfonic [11] ester conjugates, we proclaimed *p*-substituted pyridine ring as the most appropriate oxime supporting scaffold for experimenting with our novel carbamate esters, because this ring exhibited a better profile compared to the other pyridine analogues [9,11]. In order to provide a structure–activity relationship study, a series of *O*-carbamoyl conjugates consisted of benzyl as well as phenyl groups, bearing electron donating or withdrawing substituents, were synthesized and evaluated.

## Results and Discussion

### Synthesis

**Scheme 1:**
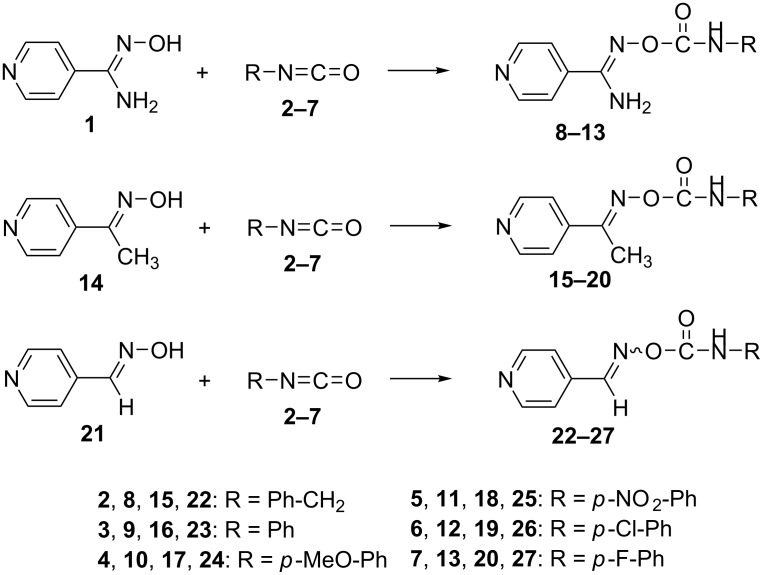
Synthesis of *O*-carbamoyl amidoximes (**8**–**13**), ethanone oximes (**15**–**20**) and aldoximes (**22**–**27**). Oxime **1** or **14** or **21**, Et_3_N (1.1 equiv), R–NCO (1.1–1.8 equiv), dry chloroform or tetrahydrofuran, Ar, 0 °C → rt or reflux, 19–97%.

All compounds were synthesized upon the reaction of the appropriate parent *p*-pyridine amidoxime **1** [58], ethanone oxime **14** [59] and aldoxime **21** [60] with the corresponding isocyanates **2**–**7** in good to excellent yields (Scheme 1). When the reactions have been performed at room temperature the yields were poor for most of the products. However, changing the conditions to reflux, the yields were extraordinarily elevated above 90%. The NMR spectra of the products obtained at rt and under reflux were identical, meaning that no isomerization or decomposition occurred and that the delivered product in both cases is the more thermodynamically stable.

Interestingly, although carbamates are important in both medicinal and polymer chemistry, besides compound **23** [61], all the rest were new. Compounds **8**–**13** were produced as a sole product, bearing the *Z*-conformation [9,43,62]. Their spectroscopic data were in accordance with the proposed structures. In IR spectra all compounds gave 2–3 absorptions above 3200 cm^−1^ for the NH_2_ and NH moieties, and the carbonyl absorption at 1700–1720 cm^−1^, characteristic of the amide moiety. This low carbonyl absorption probably indicates an intramolecular hydrogen bonding between NH_2_ and the oxime oxygen, which further verifies the *Z*-conformation of the amidoxime derivatives. The hydroxylimino structure is verified in ^1^H NMR spectra from the existence of a broad singlet peak, at 6.2–7.1 ppm integrated for two protons (NH_2_), whereas NH for the aryl derivatives **9**–**13** appeared in the area 8.9 to 10.2 ppm. In a similar way, reactions of compound **14** with isocyanates delivered a single product with the *E*-configuration [63]. In the IR spectra the NH group appears around 3200–3300 cm^−1^ and the carbonyl absorption had the characteristics of an ester, rather than an amide, at 1720 (benzyl derivative, **15**) and 1750–1786 cm^−1^ for compounds with the NH adjusted on the aromatic ring. In the ^1^H NMR spectra the NH of the aryl derivatives **16**–**20** appeared at 8.1 to 10.7 ppm and for the benzyl derivative **15** at 6.63 ppm.

In the case of aldoxime carbamates three reactions gave mixtures of inseparable *Z*-stereoisomers ≈10% along with the major *E*-stereoisomer (products **25**–**27**). It has been noted in the literature the preferable *E*-conformation for oxime carbamates [61,64–65] where ^1^H NMR spectroscopy has been used in order to distinguish between the two [61,65]. Thus, the imine benzylic proton of the *E*-stereoisomers shows a singlet in the area 8–8.7 ppm, whereas the ones belonging to a *Z*-conformation are upfield and appear between 7.3–7.6 ppm. Indeed, all aldoxime derivatives had the corresponding absorption of the oxime C–H in the area 8.3–8.8 ppm, and the three products giving the *Z*-stereoisomer showed the same proton upfield at ≈8.16 ppm.

However, we wanted to further investigate which is the most stable stereoisomer. Thus, ^1^H chemical shifts of compounds **25**–**27** were calculated using DFT computational methods with discrete solute–solvent hydrogen bond interactions [66] (see optimized structures in Supporting Information File 1). Geometry optimizations were calculated at the DFT (B3PW91) level, using the 6-31G(d) basis set. The predicted ^1^H chemical shifts with GIAO method in PBE0/6-311+G(2d,p) level of theory were in complete agreement with the experimentally found values, allowing us to verify the preferred stereoisomer of compounds **25**–**27** as *E*. Calculated ^1^H chemical shifts for C(H)=NO proton of the *E*- and *Z*-stereoisomer compared with the experimental values are shown in Table 1.

**Table 1:** Calculated ^1^H chemical shifts for C(H)=NO proton vs experimental values. (Exp. = experimental, Calcd = calculated).

Compound No	Exp. C(H)=NO chemical shift (ppm) *E*-isomer	Calcd C(H)=NO chemical shift (ppm) *E*-isomer	Exp. C(H)=NO chemical shift (ppm) *Ζ*-isomer	Calcd C(H)=NO chemical shift (ppm) *Ζ*-isomer

**25**	8.7	8.7	8.2	7.9
**26**	8.7	8.6	8.2	7.8
**27**	8.7	8.7	8.2	7.8

### DNA binding studies

**Figure 2:**
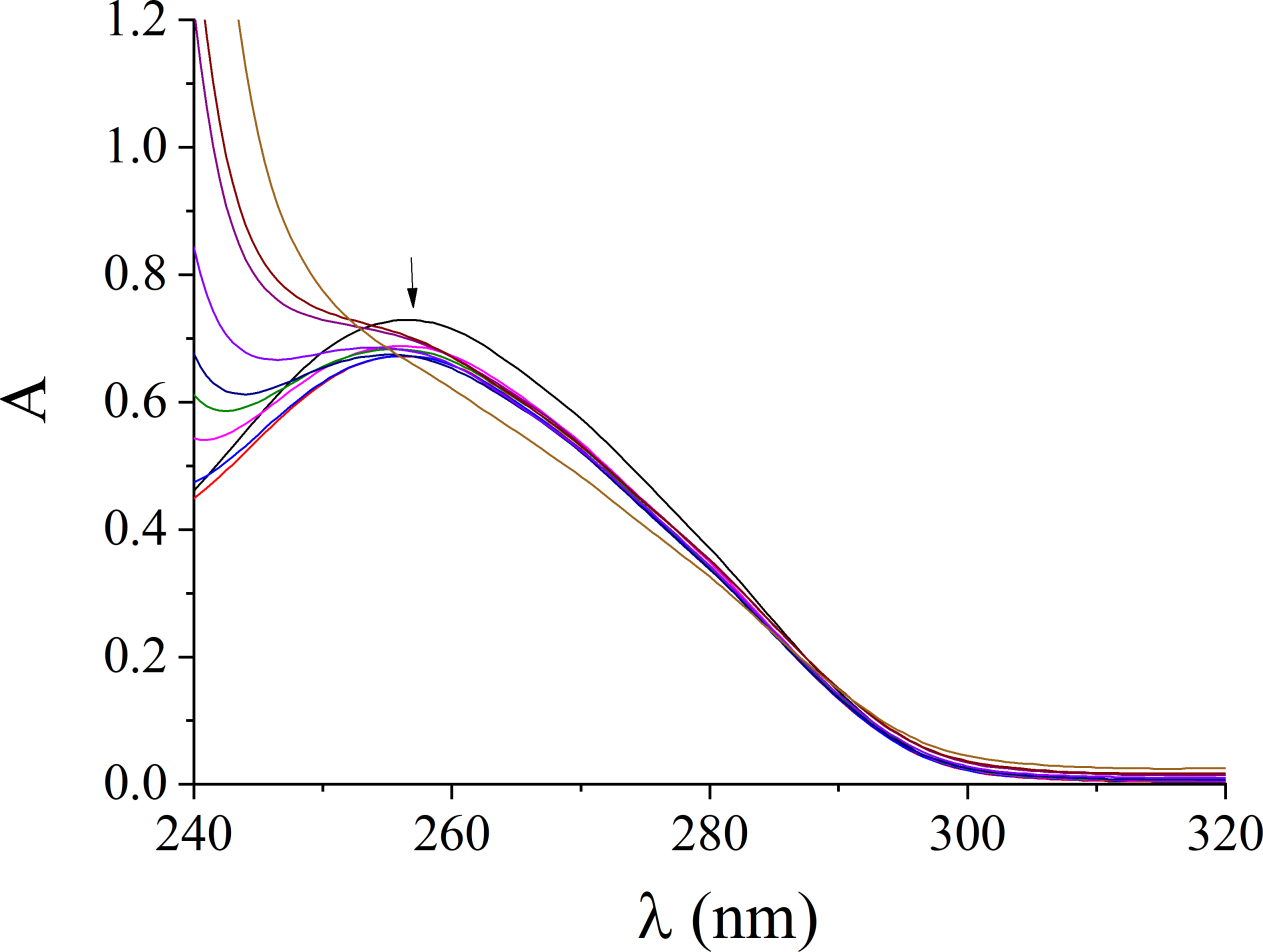
UV–vis spectra of CT DNA ([DNA] = 1.1 × 10^−4^ M) in buffer solution in the absence or presence of increasing amounts of compound **11** (*r* = 0–0.8). The arrow indicates the changes with increasing amounts of the compound.

Our previous DNA-binding studies with a series of amidoxime, ethanone oxime and aldoxime carboxylates as well as sulfonates of high, moderate or poor DNA photocleaving ability showed no substantial differences on the DNA affinities among the three kinds of oxime functionalities [9–11]. Thus, in the present study, due to the fact that all three series of oxime derivatives exhibited the same photocleaving activity we have chosen to investigate the DNA affinity of an active and a nonactive DNA photocleaver in order to, hopefully, explain the cause of the activity. Therefore, the interaction of selected compounds, i.e., **11** and **12**, with CT DNA was monitored by UV–vis spectroscopy and viscosity measurements. Additionally, the EB−displacing ability of the compounds was evaluated by fluorescence emission spectroscopy. The UV–vis spectra of a CT DNA solution were recorded in the presence of increasing concentrations of the compounds (1.0–1.1 × 10^−4^ M) and are representatively shown for compound **11** in Figure 2. The DNA UV-band with λ_max_ = 258 nm presented upon addition of the compounds a hypochromism which was accompanied by a slight red-shift; such changes may show the binding of the compounds to DNA which may result in the formation of a new conjugate between DNA and the compound under study [67].

The UV–vis spectra of the compounds (1 × 10^−4^ M) were recorded in the presence of CT DNA at increasing concentrations (diverse *r’* values) (Supporting Information File 1, Figure S-4.1). Both bands of the compound **11** (Supporting Information File 1, Figure S-4.1A) with λ_max_ at 262 nm and 388 nm show hypochromism up to 14% and 2%, respectively, while for the band appearing at 271 nm a hyperchromism of 12% is observed. In the UV–vis spectra of compound **12** (Supporting Information File 1, Figure S-4.1B), the bands located at 262 nm and 297 nm presented hypochromism of 8% and 3.5%, respectively, upon addition of CT DNA.

The K_b_ constants of the compounds were calculated by the Wolfe–Shimer equation and corresponding plots [DNA]/(ε_A_ − ε_f_) versus [DNA] (Supporting Information File 1, Figure S-4.2) [68]. The *K*_b_ constant of **11** [*K*_b(_**_11_**_)_ = 1.25 (±0.20) × 10^6^ M^−1^) is much notably than that of **12** (K_b(_**_12_**_)_ = 5.18 (±0.10) × 10^4^ M^−1^), suggesting tighter binding to DNA for compound **11**. Especially for compound **11**, the *K*_b_ constant is higher than the K_b_ constant of the classic DNA intercalator EB (*K*_b_ = 1.23 × 10^5^ M^−1^) as previously reported [69].

The data obtained by the UV–vis spectroscopy studies most probably indicate the interaction of the compounds with CT DNA. However, the exact mode of binding is not safe to be proposed before more experimental data can be collected, i.e., DNA-viscosity measurements [70].

**Figure 3:**
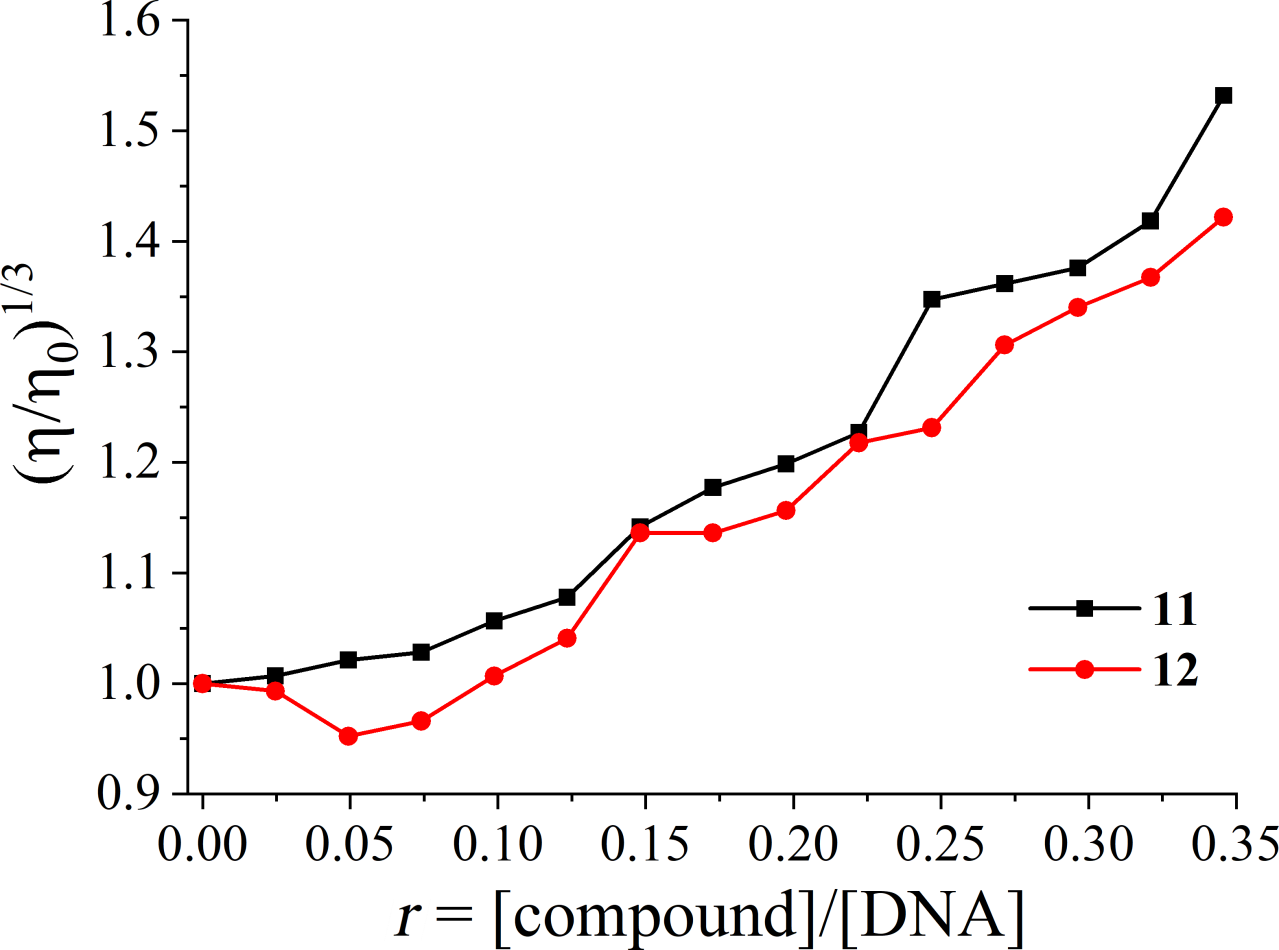
Relative viscosity (η/η_0_)^1/3^ of CT DNA (0.1 mM) in buffer solution in the presence of compounds **11** and **12** at increasing amounts.

Thus, the monitoring of the DNA viscosity changes when compounds **11** and **12** are present, may be elucidating in regard to their binding mode to DNA. As known, the changes of the relative DNA viscosity (η/η_o_)^1/3^ are proportionally related to changes of the relative DNA length (*L*/*L*_0_) [71]. Having this relation in mind, the viscosity of a CT DNA solution (0.1 mM) was determined (Figure 3) in the presence of increasing amounts of compounds **11** and **12** (up to the value of *r* = 0.35).

For compound **11** the DNA viscosity increases in the presence of the compound. For compound **12** the DNA viscosity presents a slight decrease up to an *r* value of 0.07, and for higher concentrations a noteworthy increase may be observed. Considering the overall changes of the DNA viscosity in the presence of compounds **11** and **12**, we may suggest that compound **11** shows the behaviour of a typical intercalator while compound **12** may initially interact to CT DNA probably by nonclassical intercalation (i.e., as groove-binder) and as a subsequent step it may probably intercalate within the CT DNA base pairs [11]. Such features may obviously shed light to the findings from the UV–vis spectroscopic studies.

Given the results derived from DNA-viscosity measurements suggesting intercalation as possible interaction mode between compounds **11** and **12** and CT DNA, the determination of the ability of the compounds to displace EB from the EB–DNA conjugate may further clarify and verify their intercalating ability. EB–DNA conjugate exhibits an intense fluorescence emission band at 592 nm, when its solution is excited at 540 nm. Compounds **11** and **12** have not presented any appreciable fluorescence emission either alone in solution or in the co-existence of CT DNA or EB under the same experimental conditions (λ_excitation_ = 540 nm at room temperature). Thus, the quenching observed in an EB–DNA solution upon addition of the compounds **11** and **12** may reveal their competition to EB for the DNA-intercalation sites as monitored by fluorescence emission spectroscopy with λ_excitation_ = 540 nm.

**Figure 4:**
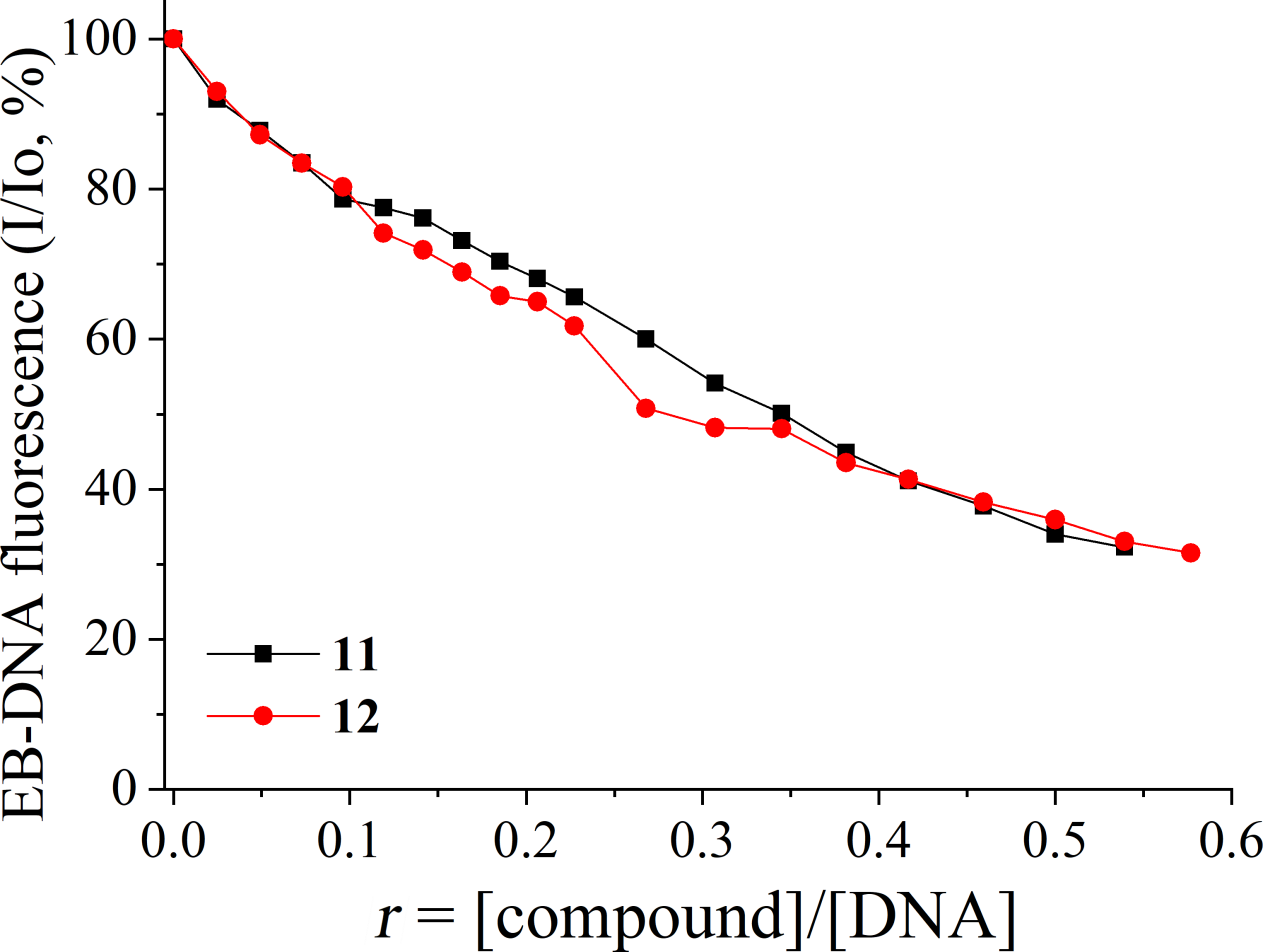
Plot of EB-DNA relative fluorescence emission intensity at λ = 592 nm (*I*/*I*_0_, %) vs *r* (= [compound]/[DNA]) in the presence of compounds **11** and **12** (up to 32.3% of the initial EB–DNA fluorescence intensity for **11** and 31.5% for **12**).

A significant quenching of the EB–DNA fluorescence (up to 68.5% of the initial fluorescence for compound **12** (Figure 4) was found in the presence of the compounds. The as-observed quenching (which is in good agreement (*R* = 0.99) with the linear Stern–Volmer equation [72]) may be attributed to the competition of compounds **11** and **12** with EB for the DNA-intercalation sites and may indirectly suggest that the compounds may bind to CT DNA via intercalation [73–74].

The values of the Stern–Volmer constant (*K*_SV_) were calculated from the corresponding Stern–Volmer plots (Supporting Information File 1, Figure S–4.3). The rather high *K*_SV_ values (*K*_sv(_**_11_**_)_ = 3.20 (±0.08) × 10^5^ M^−1^ and *K*_sv(_**_12_**_)_ = 9.16 (±0.16) × 10^4^ M^−1^) may verify the tight binding of the compounds to CT DNA [11,73–74]. More specifically, the *K*_SV_ of compound **11** is higher than that of compound **12**, probably suggesting tighter binding to DNA for compound **11** via intercalation, in accordance to the *K*_b_ constants and the conclusions from the DNA-viscosity measurements.

### DNA photocleavage studies

Carbamoyl oxime derivatives **8**–**13**, **15**–**20** and **22**–**27** (500 μΜ) were incubated as DMF solutions, with the supercoiled circular pBluescript KS II plasmid DNA (Form I) and this mixture was irradiated with UV light (312 nm) under aerobic conditions at room temperature for 30 min. All experiments were realized at minimum three times, including incubations of all compounds with DNA in dark. All compounds showed at least some UV absorption at the area of irradiation (Figures S–5.1, S–5.2, and S–5.3, Supporting Information File 1). In the presence of the halogenated compounds **12**–**13**, **19**–**20** and **26**–**27** the double helix of the supercoiled plasmid DNA (Form I) suffered single-stranded (ss) nicks, generating the relaxed circular DNA (Form II). In several cases, double-stranded (ds) nicks were generated and linear DNA (Form III) appeared as well.

**Figure 5:**
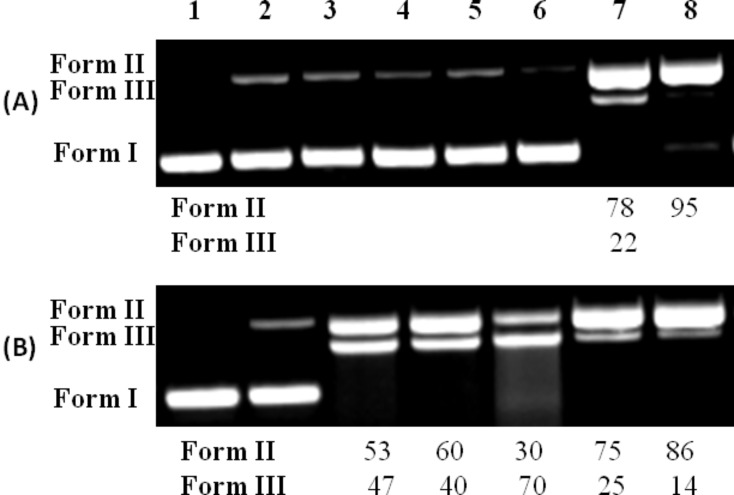
DNA photocleavage of amidoxime carbamates at a concentration of 500 μM and mechanistic studies of aldoxime carbamate **26**. Gel electrophoreses pictures. For (A) and (B) wells 1 and 2 represent DNA in dark and DNA UV irradiated, respectively. Photo (A): wells 3–8: DNA + carbamoyl amidoximes (**8** or **9**, or **10**, or **11**, or **12**, or **13**, respectively) + UV irradiation; Photo (B): Mechanistic studies involved by derivative **26** under UV irradiation: well 3: DNA + **26**; well 4: DNA + **26** + argon; well 5: DNA + **26** + DMSO (20%); well 6: DNA + **26** + NaN_3_ (20 mM); well 7: DNA + **26** + D_2_O; Legends: The % calculated damage of DNA via its conversion to Form II and Form III.

The results regarding the carbamoyl amidoxime **8**–**13** are depicted in Figure 5A. Ethanone oxime and aldoxime derivatives **15**–**20** and **22**–**27** showed quite similar results (for the comparable results see Figure S-6.1, Supporting Information File 1). None of the benzyl (Bn), phenyl (Ph), *p*-methoxyphenyl (PMP) or *p*-nitrophenyl (PNP) derivatives showed any activity [Figure 5A, wells 3–6, respectively]. On the contrary, both *p*-chlorophenyl (PCP) and *p*-fluorophenyl (PFP) showed the best photocleaving action, exhibiting a percentage of ds cleavages, as well (Figure 5A, wells 7 and 8, respectively).

Interestingly, we note that in the series of carbonyl aldoximes, ketoximes and amidoximes [9,43], as well as sulfonyl amidoximes and ethanone oximes [10–11], the PNP derivatives were by far the most active derivatives. Additionally, halogenated sulfonylethanone oxime derivatives were less reactive than the PNP one [10].

Due to the similarity in DNA photocleavage by *p*-pyridine amidoxime, ethanome oxime and aldoxime carbamoyl derivatives, mechanistic studies were performed for two active compounds from the series of amidoximes and aldoximes, **12** and **26**, Figure 5B (For the comparable results see Figure S-6.2A and B, Supporting Information File 1).

The mode of action under aerobic conditions does not involve hydroxyl radicals (in fact, DMSO seems to enhance the ds nicks), Figure 5B, well 5. It is possible that excess of this solvent facilitates the radical to escape from the cage. However, performing the same experiment for the rest of the amidoxime derivatives **8**–**11** (20% DMSO, data not shown), we did not observe any enhancement of the photocleavage above the statistical error. Counting for the singlet oxygen it is not very clear whether it is implicated or not, since the action is not improved in D_2_O, Figure 5B, wells 6 and 7, respectively. Nevertheless, the action under anaerobic conditions is unaffected (Figure 5B, well 4) and the same happens in accordance to the pH (Supporting Information File 1, Figure S-6.2C). The photocleavage is independent of pH in the range 5–10. The release of amines from carbamoyl oximes, which may imbalance the pH, and the activity in the absence of oxygen, as well as in various external pHs may be very fruitful for the treatment of solid tumours, where acidic and hypoxic environments predominate [75–76].

The DNA photocleavage experimental results along with the observations of the DNA affinity experiments, where compound **11** showed tighter binding to DNA than the chloro derivative **12** and still being inactive towards plasmid DNA upon irradiation, has prompted us to perform photochemical experiments on both latter compounds and check their photoproducts using UV spectrometry. Indeed, when they were irradiated in 1, 2, 5, 10, 15, 20, 25 and 30 min time intervals compound **12** showed the formation of an intermediate with λ_max_ at 338 nm which gradually increased, probably indicating a one-way photochemical path during irradiation process. On the other hand, the nitro derivative **11** showed the formation of an intermediate with λ_max_ at 385 nm which gradually increased for the first 3 min. This intermediate seems subsequently to act as a light filter absorbing the irradiation energy and to be converted to other products not allowing compound **11** to act as a DNA photocleaver (Figures S-7.1 and S-7.2, Supporting Information File 1). The study over those two compounds and the explanation of their behaviour has been attempted with more experimentation and is presented below.

### A computational study and photochemical aspects of compounds **11** and **12**

The ground state structures (S_0_) for both molecules **12** and **11** in their *Z*-conformations are similar (Figure S-8.1A and B, Supporting Information File 1) with the length of the N–O bond varying between *r*(N–O) = 1.423 (**12**) and 1.425 Å (**11**). (Table S-8.1, Supporting Information File 1).

The Franck–Condon (FC) vertical excitation energies (Δ*E_e_*_x_) of both molecules in their first triplet (T_1_) and singlet (S_1_) excited states were calculated (Table S-8.2, Supporting Information File 1) using additionally, in order to obtain better excitation energies and wavelengths, the PBE0 functional on the optimized (B3PW91/6-31G(d)) ground state geometries. It is shown, that **11** has lower excitation energies than **12** and this must be reflected in the difference between the two substituents (Cl and NO_2_).

Although the two molecules have essentially the same basic chromophore groups when we examine the transitions (S_0_ → T_1_ and S_0_ → S_1_) we find that there are major differences in the nature of the transitions. In particular, for the transition S_0_ → T_1_ the excitation is localized on the pyridine moiety for **12**, while for **11** it is localized on the *p*-nitrophenyl group. As far as the S_0_ → S_1_ vertical transition is concerned, for molecule **12** the excitation is localized again on the pyridine ring (involving a π* molecular orbital), whereas for **11** the excitation is of π(Ο–Ν–Ο) → π*(nitrophenyl) type.

These observations differentiate the two molecules giving them different photochemical characteristics and properties. We note that the adiabatic excitation energy of **12** (including the zero-point energy, ZPE) is Δ_0_[Τ_1_ − S_0_] = 53.40 kcal/mol, which corresponds to a wavelength of 535.6 nm (visible region). The ground state (S_0_) bond dissociation energies (BDE), *D*_0_, for both molecules under consideration, are shown in Table 2 and were calculated using Gaussian 09 software program package [77].

**Table 2:** Ground state bond dissociation energies (*D*_0_) and adiabatic excitation energies, Δ_0_[Τ_1_ − S_0_] for **11** and **12**. All numerical values in kcal/mol, in aqueous solution.

No	**11**	**12**

*D*_0_	51.54	41.38
Δ_0_[T_1_ − S_0_]	57.29	53.40

The BDE difference between the two compounds is about 10 kcal/mol, while that in their adiabatic excitation energies is 3.9 kcal/mol. Since Δ_0_ − *D*_0_ is higher for **12** than **11** someone would expect **12** to be more reactive in the T_1_ state as compared to **11**, something which is confirmed from our calculations shown below.

**Figure 6:**
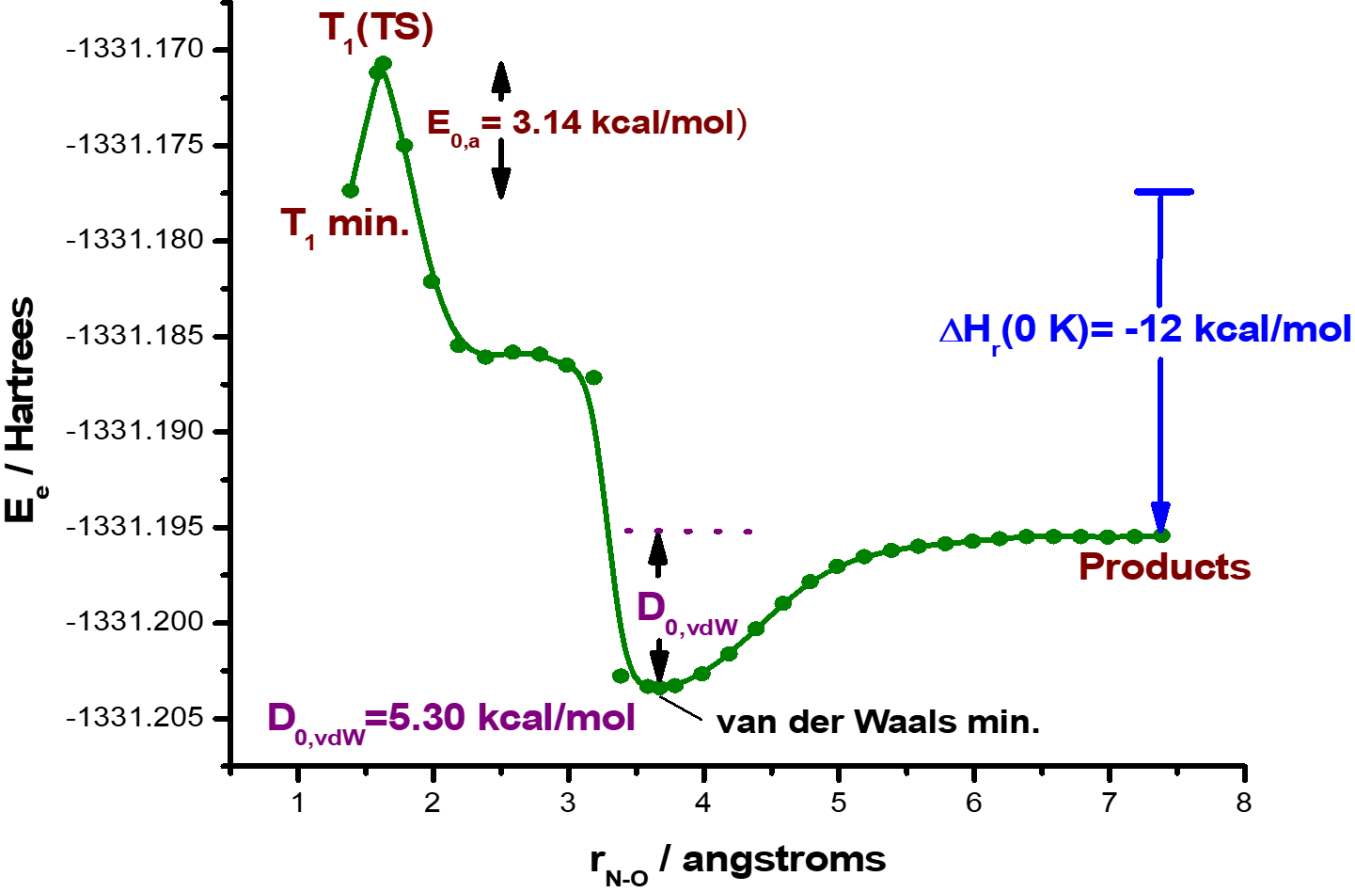
Potential energy curve for the dissociation of **12** in the first excited triplet state, T_1_. For compound **11** see Figure S-8.3, Supporting Information File 1.

It is well known that compounds characterized by triplet energies higher than BDEs should exhibit high reactivity [78–79]. After excitation to the T_1_ FC point, **12** having enough energy reaches T_1_ min. From there, dissociation of the N–O bond starts to take place and proceeds uphill through a transition state, T_1_(TS), the structure of which was optimized fully. Vibrational analysis gives a single imaginary frequency (−550.12 cm^−1^) which corresponds to the N–O bond stretching confirming that the optimized structure is a first order saddle point. Relaxed PES scan was performed until complete dissociation to the respective ground state radicals occurred. In Figure 6 we plot the variation of the energy for **12** in the T_1_ state with respect to the reaction coordinate which is the N–O bond distance, *r*_N–O_.

**Scheme 2:**
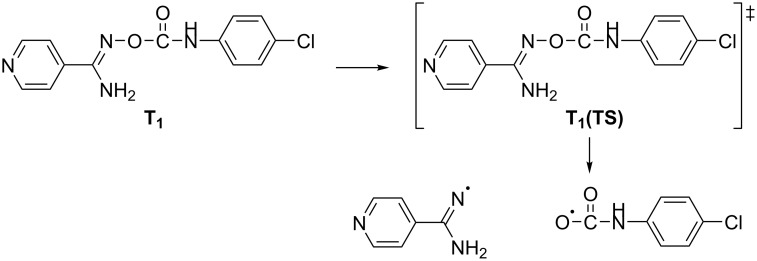
Photodissociation reaction of the derivative **12** in the T_1_ state and the formation of ground state radicals.

For the dissociation reaction of compound **12** in the triplet state, the molecule passes through a very small energy barrier and dissociates finally into its photoproducts which are the two ground state radicals indicated in Scheme 2.

The almost negligible change in geometry on going from T_1_ min to T_1_(TS) is reflected in the low value of the activation entropy, ΔS^≠^ = 0.474 cal/mol∙K, which means that there is a very small disorganization in the transition state as compared to the reactant in the T_1_ minimum state. The length of the bond N–O in the transition state is equal to 1.629 Å.

The corresponding activation energy (Equation 1) and free activation energy (Equation 2) were calculated for compound **12** and found 3.14 and 2.95 kcal/mol, respectively. These values were used in Equation 3 in order to calculate the rate constant for the N−O bond dissociation. Accordingly, *kr*, was found to be 4.27 ∙10^10^ s^−1^, which is a very large value, indicating a fast N−O bond dissociation for compound **12**. Equations 1–3 are described in the theoretical calculations section.

**Scheme 3:**
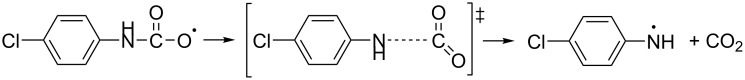
Decarboxylation reaction of the *p*-chlorophenylcarbamoyloxyl radical.

As soon as the two radicals (amidinyl and *p*-chlorocarbamoyloxyl) are formed, the second radical starts to decarboxylate according to the chemical reaction below (Scheme 3).

The activation free energy for the decarboxylation reaction is only 1.09 kcal/mol and by using Equation 4 (see theoretical calculations section) we find a rate constant *k*_r_ = 9.87∙10^11^ s^−1^, characterizing the reaction as an ultrafast one, with a corresponding life-time of the radical τ = 1ps. This is in complete agreement with the prediction made by McBurney and Walton [51] for the decarboxylation of *N*-arylcarbamoyloxyl radicals where they were expected to decarboxylate with great rapidity having almost no finite lives.

Excitation of **12** to the singlet excited state (S_1_) forces the molecule to pass over an energy barrier of approximately 7 kcal/mol dissociating further and giving ground state radicals again. For the photodissociation of **11** from either electronic excited state (T_1_, S_1_) calculations show that this is not possible since in this case the intervening energy barriers are very large, 29.37 kcal/mol for T_1_ and 31 kcal/mol for the S_1_ state. Hence, **11** does not show any photoreactivity, in accordance with our experimental results.

### Photoexcitation in the presence of a triplet state energy activator and a triplet state quencher

Triplet photosensitizers (TPSs) or triplet state energy activators are compounds that have the ability to be efficiently excited to their triplet excited state. TPSs may be used, among others, to transfer their triplet energy to other molecules that have a low yield of intersystem crossing (ISC) and inefficient production of triplet state [78,80]. Acetophenone (AP) is such a compound, that when initially excited to its first singlet excited state, exhibits a singlet-to-triplet conversion quantum yield close to 100% [81] and has been used for its triplet energy transfer [82].

**Figure 7:**
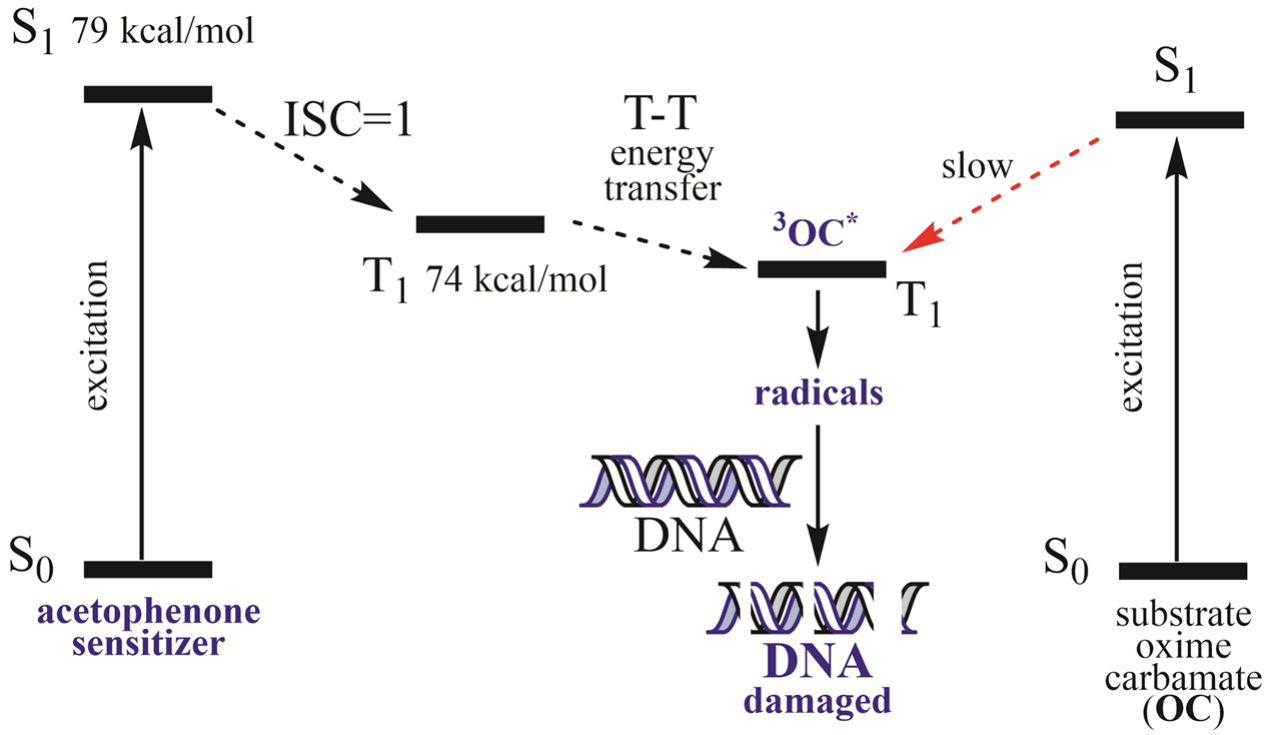
Proposed scheme showing a possible energy transfer from acetophenone sensitizer to oxime carbamate substrate.

In order to experimentally prove that DNA dissociation occurs from the triplet state of oxime carbamates, when the system has the ability to pass ISC to the triplet energy state, as in the case of compound **12** (and accordingly all halogenated compounds of the series) we have designed the following experiment: We hypothesized that, upon selective excitation oxime carbamates **8**–**13** might be excited at their triplet states via triplet state energy transfer from acetophenone as a sensitizer, dissociate to their iminyl/carbamoyloxyl and subsequent anilinyl radicals, attack DNA and cleave it (Figure 7).

**Figure 8:**
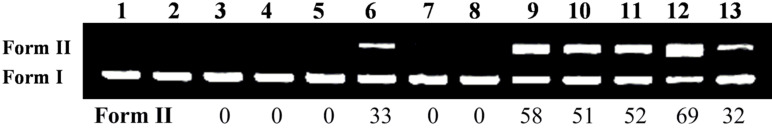
DNA photocleavage of compounds **8**–**10** and **12**–**13** at concentration of 500 μM, at 365 nm, in the absence and presence of acetophenone (AP, 1 mM, absorption ≈0.1 at 365 nm), 2 h, 10 cm distance, aerobic conditions. wells 1 and 2 represent DNA in dark and DNA UV irradiated, respectively. Photo: wells 3–8: DNA + **8**, or **9**, or **10**, or **12**, or **13** + UV, respectively; well 8: DNA + AP + UV; wells 9–13: DNA + **8**, or **9**, or **10**, or **12**, or **13** + AP + UV, respectively.

As shown in Figure 8 none of the compounds show any cleavage at 365 nm in the absence of AP (wells 3–7), nevertheless in the presence of the photosensitizer they are able to cause DNA photocleavage around 50% (wells 9–13). AP itself is inactive towards DNA, under the experimental conditions (well 8).

**Figure 9:**
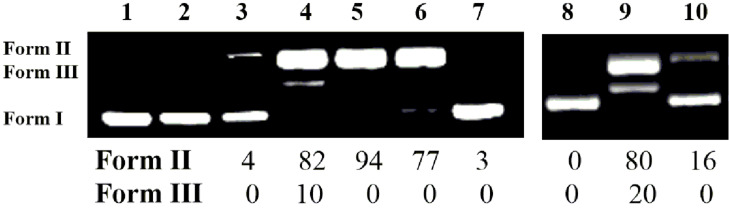
DNA photocleavage of compound **12** at a concentration of 500 μM, at 312 nm, in the absence and presence of FL and CR, as quenchers. wells 1 and 2 represent DNA in dark and DNA UV irradiated, respectively. Photo: well 3: DNA + FL (100 μM) + UV; wells 4–7: DNA + **12** + UV + FL (10, or 25, or 50, or 100 μM, respectively; well 8: DNA + CR (200 μM) + UV; lane 9: DNA + **12** + UV; lane 10: DNA + **12** + CR (200 μM) + UV.

Finally, we have used triplet energy quenchers such as fluorenone (FL) and carotene (CR), which exhibit low triplet state energy (≈50 and 19 kcal/mol, respectively) [83] and may, acting as a triplet quencher, accept energy transfer from oxime carbamate **12**, having now the latter compound as the sensitizer. In this case we expect to have decrease or elimination of the activity of compound **12**. The results are shown in Figure 9.

It is obvious that in the presence of increasing concentrations of FL the activity of **12** gradually decreases (wells 4–6) until it is totally eliminated (well 7). Additionally, in the presence of CR as a quencher, the energy of the triplet state of **12** is transferred to CR and DNA is not damaged (well 10).

## Conclusion

*p*-Pyridyl oxime carbamates bearing electron-donating or electron-withdrawing substituents on the aromatic carbamate group and an amine, a methyl or a hydrogen on the imine moiety, were successfully synthesized in high yields and subjected to UV irradiation in the presence of plasmid DNA. Not all derivatives were active, nevertheless all amidoxime, ethanone oxime and aldoxime derivatives showed similar effects, indicating the cause of action to be considered on the carbamate moiety rather than the oxime. The affinity of selective compounds with DNA was good to excellent, as verified with extensive CT DNA-affinity studies.

A deeper insight in the photochemical behaviour of the nitro (**11**) and the chloro (**12**) derivatives using UV spectroscopy in the absence of DNA was indicative of the disruption of **11** whereas **12** showed a gradual formation of one product. A theoretical photochemical dissociation study resulted to the observation that **12** has the ability to dissociate, as expected, with homolysis of its N–O bond overcoming very low energy barriers with high rate constants, on the contrary to **11**, where its photochemical activity seems to be located at the nitro group. The explanation of the activity seems to be the ability of **12** to obtain its triplet state and create active radicals able to abstract hydrogen atoms from DNA and cause its damage. This was experimentally verified using acetophenone, as a triplet state sensitizer, which transferred its energy to inactive oxime carbamates and enabled them to damage DNA. Additionally, the activity of compound **12** was diminished when triplet state quenchers as fluorenone and carotene were introduced into the irradiated DNA mixture.

Accordingly, halogenated carbamate derivatives, of both amidoxime, ethanone oxime and aldoxime derivatives showed, similarly, significant DNA photocleavage. Mechanistic studies on DNA photocleavage showed that these “synthetic nucleases” act independently of oxygen and pH. As photobase generators, upon the homolysis of their N–O bond, oxime carbamates are able to release amines which in in vivo action may imbalance the pH of the tissues, and still retain the ability to create radicals. The same applies to hypoxic environments and anaerobic conditions.

Finally, with this study, we have shown that oxime carbamate derivatives have the potential to act as novel effective photobase generating DNA-photocleavers with predicted DNA photocleaving, able, as well, to be subjected to photoinduced sensitizing. Thus as a novel class of photocleavers, oxime carbamates may serve in the discovery of new leads for “on demand” biotechnological, technological and medical applications.

## Experimental

### Materials and methods

All commercially available reagent grade chemicals and solvents were used without further purification. Dry solvents were purchased from Sigma–Aldrich Co. Calf-thymus (CT) DNA, ethidium bromide (EB), NaCl and trisodium citrate were purchased from Sigma–Aldrich Co. CT DNA stock solution was prepared according to standard procedures [84]. The CT DNA concentration was determined by the UV absorbance at 260 nm after 1:20 dilution using ε = 6600 M^–1^cm^–1^ [85].

High-resolution mass spectra (HRMS) were recorded on micrOTOF GC–MS QP 5050 Shimadzu single−quadrupole mass spectrometer. UV–visible (UV–vis) spectra were recorded on a Hitachi U-2001 dual beam spectrophotometer. Fluorescence emission spectra were recorded in solution on a Hitachi F-7000 fluorescence spectrophotometer. Viscosity experiments were carried out using an ALPHA L Fungilab rotational viscometer equipped with an 18 mL LCP spindle and the measurements were performed at 100 rpm. All samples were irradiated with Philips 2 × 9W/01/2P UV-B narrowband lamps at 312 nm.

Melting points were measured on a Kofler hot-stage apparatus or a melting point meter M5000 KRÜSS, and are uncorrected. FTIR spectra were obtained in a Perkin–Elmer 1310 spectrometer using potassium bromide pellets. NMR spectra were recorded on an Agilent 500/54 (500 MHz and 125 MHz for ^1^H and ^13^C, respectively) spectrometer using CDCl_3_, and/or DMSO-*d*_6_ as solvent. Chemical shifts are given in ppm and *J* values in Hz using solvent as an internal reference. All reactions were monitored on commercial available pre-coated TLC plates (layer thickness 0.25 mm) of Kieselgel 60 F254. Yields were calculated after recrystallization.

### Synthesis of carbamates

All compounds were synthesized upon the reaction of the appropriate parent *p*-pyridine amidoxime **1** [58], ethanone oxime **14** [59] and aldoxime **21** [60] with the corresponding isocyanates **2**–**7** in good to excellent yields (Scheme 1). All carbamates are new, besides compound **23** [61]. For each compound, all spectroscopic and other data were collected in order to establish identity. General procedures for the syntheses, as well as data and pictures of all spectra are provided in Supporting Information File 1.

### Interaction with CT DNA

#### DNA-binding studies with UV–vis spectroscopy

The binding constants of compounds **11** and **12** to CT DNA (*K*_b_) were calculated by the Wolfe–Shimer equation [11,68] using UV–vis spectroscopy, in order to estimate their interaction of with CT DNA.

#### DNA-viscosity studies

The viscosity of CT DNA ([DNA] = 0.1 mM) in buffer solution (150 mM NaCl and 15 mM trisodium citrate at pH 7.0) was measured in the presence of increasing amounts of compounds **11** and **12** (up to the value of *r* = 0.35) [11].

#### EB-displacement studies

The ability of compounds **11** and **12** to displace EB from its DNA–EB conjugate was investigated by fluorescence emission spectroscopy. The Stern–Volmer constant (*K*_SV_, in M^−1^) was used to evaluate the quenching efficiency for each compound according to the Stern–Volmer equation [11,72].

### DNA cleavage experiments

All synthesized *O*-carbamoyl oximes were incubated with supercoiled circular Bluescript KS II DNA and irradiated at 312 or 365 nm for 30 min and 2 h, respectively. The mixture was subjected to electrophoresis, the gel was visualized by 312 nm UV transilluminator and photographed. Finally, quantitation of DNA-cleaving activities was performed using the program “Image J” available at the site http://rsb.info.nih.gov/ij/download.html and the single-strand (ss%) as well as double-strand (ds%) damage was calculated, using a correction factor of 1.43 [11].

### Theoretical calculations

#### Calculations for the photodegradation of carbamates

The structures, properties and the basic photochemistry of compounds **11** and **12** was studied using the density functional theory (DFT) method [86–89] and the functional B3PW91 along with the 6-31 G(d) basis set. This functional has been found to describe accurately the bond dissociation process [90]. Furthermore, all calculations were carried out in aqueous solution using the polarizable continuum model with the integral equation formalism (IEFPCM). All relevant structures were optimized fully and characterized accordingly as stationary points (minima or maxima) on the corresponding potential energy surfaces (PESs).

Equations 1–5 were used for the calculations of the rates and the physicochemical data of the N−O bond dissociation of the most active compound **12** in radicals. The corresponding activation energy and free energy of activation are given in Equation 1 and Equation 2, respectively:

[1] $$\Delta E_{0}‡\;=\;E_{0}\left({\text{T}}_{1}/TS\right)\;-\;E_{0}\left({\text{T}}_{1}{}_{\;min}\right)$$

[2] $$\Delta G‡\;=\;G\left({\text{T}}_{1}/TS\right)\;-\;G\left({\text{T}}_{1\;min}\right)$$

For the calculation of the rate constant, *k_r_*, the Eyring’s classical Equation 3 was used, where in the above equation *k_B_* is the Boltzmann’s constant (1.380662∙10^−23^ J/K), *h* is the Planck’s constant (6.626176∙10^−34^ J∙s), *R* is the universal gas constant (1.987 kcal/mol∙K) and *T* is the absolute temperature in K.

[3] $$k_{\text{r}}=\kappa \frac{k_{\text{B}}\cdot T}{h}\cdot e^{-\Delta G^{\neq }/RT}$$

Improvement of the Eyring’s equation, incorporating the quantum mechanical tunnelling coefficient, κ, was also used, Equation 4. The quantum phenomenon in this case is the tunnelling of the electrons of the bond under dissociation, that is, the N−O bond which constitutes the potential well.

[4] $$k_{\text{r}}=\kappa \frac{k_{\text{B}}\cdot T}{h}\cdot \frac{Q^{\neq }}{Q_{\text{R}}}e^{-\Delta E_{0}^{\neq }/RT}$$

In Equation 4 *Q*^≠^ and *Q*_R_ represent the molecular partition functions in the transition state and in T_1_ min with their values being 0.241907∙10^24^ and 0.184606∙10^24^, respectively. Additionally, Equation 5 was used to calculate the quantum mechanical tunneling coefficient by employing the Skodje–Truhlar potential for parabolic potential barriers [91].

[5]
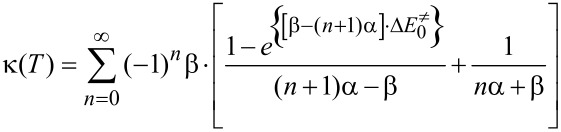


In Equation 5 the parameters α = 2π/h∙Im(ν^≠^) = 5.74672∙10^20^ J^−1^ and β = 1/*k*_B_∙*T* = 2.42928∙10^20^ J^−1^ are defined through the fundamental constants, ν^≠^ is the imaginary frequency of the transition state corresponding to that normal mode of vibration which takes the transition state towards the product without a restoring force. In our case, ν^≠^ = 550.12*i* cm^−1^ and Im(ν^≠^) = 550.12 cm^−1^ = 1.64921∙10^13^ s^−1^, which is a real number.

Finally, the N−O bond energies were calculated according to previously reported methods [9].

## Supporting Information

The Supporting Information features 1) general procedures for the synthesis of all compounds and data analysis; 2) ^1^H NMR and ^13^C NMR of amidoxime, ethanone oxime and aldoxime carbamates; 3) Optimized structures of compounds **25**–**27** with the ab initio DFT computational methods, 4) DNA binding studies; 5) UV absorption spectra of all amidoxime, ethanone oxime and aldoxime carbamates; 6) Gel electrophoresis pictures of all amidoxime, ethanone oxime and aldoxime carbamate; 7) UV absorption spectra of amidoxime carbamates **11** and **12** under irradiation; 8) A computational study and photochemical aspects of compounds **11** and **12**.

File 1 Experimental part.
