# Supplementary material for: p-Pyridinyl oxime carbamates: synthesis, DNA binding, DNA photocleaving activity and theoretical photodegradation studies
Source: Beilstein J Org Chem. 2020 Mar 9;16:337–50. doi: 10.3762/bjoc.16.33 (PMC7082612; doi:10.3762/bjoc.16.33)
Supplement: File 1 — Experimental part. [file Beilstein_J_Org_Chem-16-337-s001.pdf]

BEILSTEIN JOURNAL OF ORGANIC CHEMISTRY

Supporting Information

for

p-Pyridinyl oxime carbamates: synthesis, DNA binding, DNA photocleaving activity and theoretical photodegradation studies

Panagiotis S. Gritzapis, Panayiotis C. Varras, Nikolaos-Panagiotis Andreou, Katerina R. Katsani, Konstantinos Dafnopoulos, George Psomas, Zisis V. Peitsinis, Alexandros E. Koumbis and Konstantina C. Fylaktakidou

Beilstein J. Org. Chem. **2020,** *16,* 337–350. doi:10.3762/bjoc.16.33

Experimental part

License and Terms: This is a supporting information file under the terms of the Creative Commons Attribution License (https://creativecommons.org/licenses/by/4.0). Please note that the reuse, redistribution and reproduction in particular requires that the authors and source are credited.
The license is subject to the *Beilstein Journal of Organic Chemistry* terms and conditions: (https://www.beilstein-journals.org/bjoc)

Table of contents

1.	***General procedures for the synthesis of all compounds and data analysis***	S3
2.	***^{1}H NMR and ^{13}C NMR of amidoxime, ethanone oxime and aldoxime carbamates***	S7
	(*Z*)-*N'*-[(benzyl-carbamoyl)oxy]isonicotinimidamide (**8**)	S7
	(*Z*)-*N'*-[(phenyl-carbamoyl)oxy]isonicotinimidamide (**9**)	S9
	(*Z*)-*N'*-[(4-methoxyphenyl-carbamoyl)oxy]isonicotinimidamide (**10**)	S11
	(*Z*)-*N'*-[(4-nitrophenyl-carbamoyl)oxy]isonicotinimidamide (**11**)	S13
	(*Z*)-*N'*-[(4-chlorophenyl-carbamoyl)oxy]isonicotinimidamide (**12**)	S15
	(*Z*)-*N'*-[(4-fluorophenyl-carbamoyl)oxy]isonicotinimidamide (**13**)	S17
	(*E*)-1-(pyridin-4-yl)ethanone O-benzylcarbamoyl oxime (**15**)	S19
	(*E*)-1-(pyridin-4-yl)ethanone O-phenylcarbamoyl oxime (**16**)	S21
	(*E*)-1-(pyridin-4-yl)ethanone O-4-methoxyphenylcarbamoyl oxime (**17**)	S23
	(*E*)-1-(pyridin-4-yl)ethanone O-4-nitrophenylcarbamoyl oxime (**18**)	S25
	(*E*)-1-(pyridin-4-yl)ethanone O-4-chlorophenylcarbamoyl oxime (**19**)	S27
	(*E*)-1-(pyridin-4-yl)ethanone O-4-fluorophenylcarbamoyl oxime (**20**)	S29
	(*E*)-isonicotinaldehyde O-benzylcarbamoyl oxime (**22**)	S31
	(*E*)-isonicotinaldehyde O-phenylcarbamoyl oxime (**23**)	S33
	(*E*)-isonicotinaldehyde O-4-methoxyphenylcarbamoyl oxime (**24**)	S35
	(*E*)/(*Z*)-isonicotinaldehyde O-4-nitrophenylcarbamoyl oxime (**25**)	S37
	(*E*)/(*Z*)-isonicotinaldehyde O-4-chlorophenylcarbamoyl oxime (**26**)	S39
	(*E*)/(*Z*)-isonicotinaldehyde O-4-fluorophenylcarbamoyl oxime (**27**)	S41
3.	***Optimized structures of compounds 25-27 with the ab initio DFT computational methods***	S43
4.	***DNA binding studies***	S44
	**Figure S-4.1.** UV–vis spectra of DMSO solution of compound (A) **11** and (B) **12** in the presence of increasing amounts of CT DNA.	S44
	**Figure S-4.2.** Plot of [DNA]/(ε_A-ε_f) versus [DNA] for compound (A) **11** and (B) **12**.	S44
	**Figure S-4.3.** Stern-Volmer quenching plot of EB bound to CT DNA for compound (A) **11** and (B) **12**.	S44
5.	***UV absorption spectra of amidoxime, ethanone oxime and aldoxime carbamates***	S45
	**Figure S-5.1.** UV–vis spectra of amidoxime carbamates	S45
	**Figure S-5.2.** UV–vis spectra of ethanone oxime carbamates	S45
	**Figure S-5.3.** UV–vis spectra of aldoxime carbamates	S45
6.	***Gel electrophoresis pictures of amidoxime, ethanone oxime and aldoxime carbamates***	S46
	**Figure S-6.1.** Comparative gel electrophoresis pictures of all carbamates	S46
	**Figure S-6.2.** Mechanistic studies involved by derivatives **12** and **26** and pH effect on compound **12**.	S46
7.	***UV absorption spectra of amidoxime carbamates 11 and 12 under irradiation***	S47
	**Figure S-7.1.** UV–vis spectra of amidoxime carbamate **11**	S47
	**Figure S-7.2.** UV–vis spectra of amidoxime carbamate **12**	S47
8.	***A computational study and photochemical aspects of compounds 11 and 12***	S48
	**Figure S-8.1.** Ground state (S_0) structures of **12** (A) and **11** (B).	S48
	**Table S-8.1.** Molecular geometries of compounds **12** and **11**.	S48
	**Table S-8.2.** Franck-Condon excitation energies.	S48
	**Figure S-8.3.** PES for the dissociation of M2 in the first excited triplet state.	S49
	**8.4.** Mathematical Appendix	S49

1. Synthesis of O−carbamoyl oximes

1.1. General procedure for the synthesis of O−carbamoyl amidoximes

(*Z*)-*N'*-Hydroxyisonicotinimidamide **1** [68] (274 mg, 2 mmol) was dissolved in dry chloroform (12 mL) under Ar atmosphere. Triethylamine (0.3 mL, 2.2 mmol) was added at 0 °C, followed by slow addition (15 min) of the proper isocyanate (2.2 mmol). The mixture was stirred at rt or refluxed for the indicated period of time. Then, water (30 mL) was added and the mixture was extracted with dichloromethane (3 × 30 mL). After drying (Na_2SO_4) the organic solvent was removed in a rotary evaporator and the crude residue was either recrystallized or subjected to a column chromatography and then recrystallized.

1.2. General procedure for the synthesis of O-carbamoyl ethanone oximes

(*E*)-1-(Pyridin-4-yl)ethan-1-one oxime **14** [69] (136 mg, 1 mmol) was dissolved in dry chloroform or tetrahydrofuran (6 mL) under Ar atmosphere. Triethylamine (0.15 mL, 1.1 mmol) was added at 0 °C, followed by slow addition (15 min) of the proper isocyanate (1.1–1.8 mmol). The mixture was stirred at rt or refluxed for the indicated period of time. Then, water (20 mL) was added and the mixture was extracted with dichloromethane (3 × 20 mL). After drying (Na_2SO_4) the organic solvent was removed in a rotary evaporator and the crude residue was either recrystallized or subjected to a column chromatography and then recrystallized.

1.3. General procedure for the synthesis of O-carbamoyl aldoximes

(*E*)-Isonicotinaldehyde oxime **21** [70] (244 mg, 2 mmol) was dissolved in tetrahydrofuran or other indicated solvent (25 mL) under Ar atmosphere. Triethylamine (0.3 mL, 2.2 mmol) was added at 0 °C, followed by slow addition (15 min) of the proper isocyanate (2.2 mmol). The mixture was stirred at rt or refluxed for the indicated period of time. Then, water (30 mL) was added and the mixture was extracted with dichloromethane (3 × 30 mL). After drying (Na_2SO_4) the organic solvent was removed in a rotary evaporator and the crude residue was either recrystallized or subjected to a column chromatography and then recrystallized.

1.4. Data analysis of O-carbamoyl amidoximes 8–13, O-carbamoyl ethanone oximes 15−20, and O-carbamoyl amidoximes 22−27

(Z)-N'-[(Benzyl-carbamoyl)oxy]isonicotinimidamide (8).

Reaction time: 1 h (rt); method of purification: recrystallization; yield: 408 mg (75%); white crystals, mp 132 °C (ethyl acetate/hexanes); IR (KBr): 3378, 3292, 3111, 1701 cm^{-1}; ^{1}H NMR (500 MHz, $CDCl_3$ + DMSO-*d*$_6$) δ 4.28 (d, *J* = 6.0 Hz, 2H), 6.13 (br s, 2H), 6.97 (br t, *J* = 5.1 Hz, 1H), 7.04–7.19 (m, 5H), 7.50 (d, *J* = 4.9 Hz, 2H), 8.45 (d, *J* = 5.0 Hz, 2H) ppm; ^{13}C NMR (125 MHz, $CDCl_3$ + DMSO-*d*$_6$) δ 44.0, 120.2, 126.5, 126.6, 127.8, 137.9, 138.3, 149.2, 151.9, 155.2 ppm; HRMS (ESI) calc $C_{14}H_{15}N_4O_2$ $[M + H]^+$ 271.1190; found 271.1189.

(Z)-N'-[(Phenyl-carbamoyl)oxy]isonicotinimidamide (9).

Reaction time: 2 h (rt); method of purification: column chromatography (eluent: CH_2Cl_2/MeOH 20/1); yield: 353 mg (69%); beige crystals, mp 168 °C (MeOH/H_2O); IR (KBr): 3413, 3304, 1718 cm^{-1}; ^{1}H NMR (500 MHz, $CDCl_3$ + DMSO−*d*$_6$) δ 6.63 (br s 2H), 7.00 (t, *J* = 7.4 Hz, 1H), 7.24 (t, *J* = 7.8 Hz, 2H), 7.46 (d, *J* = 7.8 Hz, 2H), 7.71 (d, *J* = 6.0 Hz, 2H), 8.61 (d, *J* = 6.0 Hz, 2H), 8.98 (br s, 1H) ppm; ^{13}C NMR (125 MHz, $CDCl_3$ + DMSO−*d*$_6$) δ 118.2, 119.9, 122.2, 127.6, 136.8, 137.8, 148.7, 151.3, 152.3 ppm; HRMS (ESI) calc $C_{13}H_{13}N_4O_2$ $[M + H]^+$ 257.1033; found 257.1030.

(Z)-N'-[(4-Methoxyphenyl-carbamoyl)oxy]isonicotinimidamide (10).

Reaction time: 24 h (rt); method of purification: column chromatography (eluent: CH_2Cl_2/MeOH 40/3); yield: 401 mg (70%); grey crystals, mp 167 °C (ethyl acetate); IR (KBr): 3468, 3326, 1718 cm^{-1}; ^{1}H NMR (500 MHz, DMSO-*d*$_6$) δ 3.73 (s, 3H), 6.91 (d, *J* = 9.0 Hz, 2H), 7.04 (br s, 2H), 7.44

(d, J = 9.0 Hz, 2H), 7.84 (d, J = 6.1 Hz, 2H), 8.69 (d, J = 6.1, Hz, 2H), 9.24 (s, 1H) ppm; ^{13}C NMR (125 MHz, DMSO-d_6) δ 55.2, 113.9, 121.0, 121.4, 131.3, 138.8, 150.0, 152.7, 153.2, 155.4 ppm; HRMS (ESI) calc $C_{14}H_{15}N_4O_3$ [M + H]$^+$ 287.1139; found 287.1135.

(Z)-N'-[(4-Nitrophenyl-carbamoyl)oxy]isonicotinimidamide (11).

Reaction time: 4 h (reflux); method of purification: recrystallization; yield: 556 mg (92%); yellow-orange crystals, mp 202 °C (EtOH/H_2O); IR (KBr): 3482, 3358, 3316, 1719 cm^{-1}; ^{1}H NMR (500 MHz, DMSO-d_6) δ 7.10 (br s, 2H), 7.79 (d, J = 6.1 Hz, 2H), 7.80 (d, J = 9.1 Hz, 2H), 8.24 (d, J = 9.3 Hz, 2H), 8.71 (d, J = 6.1 Hz, 2H), 10.14 (br s 1H) ppm; ^{13}C NMR (125 MHz, DMSO-d_6) δ 118.4, 121.0, 125.0, 138.6, 142.0, 145.1, 150.0, 151.9, 154.6 ppm; HRMS (ESI) calc $C_{13}H_{12}N_5O_4$ [M + H]$^+$ 302.0884; found 302.0886.

(Z)-N'-[(4-Chlorophenyl-carbamoyl)oxy]isonicotinimidamide (12).

Reaction time: 24 h (reflux); method of purification: recrystallization; yield: 522 mg (90%); grey crystals, mp 197 °C (EtOH/H_2O); IR (KBr): 3467, 3327, 3176, 1703 cm^{-1}; ^{1}H NMR (500 MHz, $CDCl_3$ + DMSO-d_6) δ 6.59 (br s 2H), 7.20 (d, J = 8.8 Hz, 2H), 7.46 (d, J = 8.8 Hz, 2H), 7.70 (d, J = 6.1 Hz, 2H), 8.61 (d, J = 6.0 Hz, 2H), 9.08 (br s, 1H) ppm; ^{13}C NMR (125 MHz, $CDCl_3$ + DMSO-d_6) δ 119.6, 120.1, 126.9, 127.6, 135.8, 137.9, 148.8, 151.3, 152.5 ppm; HRMS (ESI) calc $C_{13}H_{12}ClN_4O_2$ [M + H]$^+$ 291.0643; found 291.0650, 293.0614 (3:1).

(Z)-N'-[(4-Fluorophenyl-carbamoyl)oxy]isonicotinimidamide (13).

Reaction time: 4 h (reflux); method of purification: recrystallization; yield: 525 mg (96%); grey crystals, mp 182 °C (EtOH/H_2O); IR (KBr): 3408, 3297, 3158, 1714 cm^{-1}; ^{1}H NMR (500 MHz, DMSO-d_6) δ 7.06 (br s, 2H), 7.17 (t, $^3J_{HF}$ = $^3J_{HH}$ = 8.9 Hz, 2H), 7.56 (dd, $^3J_{HH}$ = 8.9 Hz, $^4J_{HF}$ = 5.0 Hz, 2H), 7.82 (d, J = 5.9 Hz, 2H), 8.69 (d, J = 6.0 Hz, 2H), 9.47 (br s, 1H) ppm; ^{13}C NMR (125 MHz, DMSO-d_6) δ 115.3 (d, $^2J_{CF}$ = 22.2 Hz), 121.0, 121.4 (d, $^3J_{CF}$ = 7.9 Hz), 134.8 (d, $^4J_{CF}$ = 2.5 Hz), 138.8, 150.0, 152.6, 153.5, 158.1 (d, $^1J_{CF}$ = 238.0 Hz) ppm; HRMS (ESI) calc $C_{13}H_{12}FN_4O_2$ [M + H]$^+$ 275.0939; found 275.0934.

(E)-1-(Pyridin-4-yl)ethanone O-benzylcarbamoyl oxime (15).

Reaction time: 24 h (reflux); solvent: chloroform; method of purification: column chromatography (eluent: ethyl acetate/hexanes 2/1); yield: 207 mg (77%); off-white crystals, mp 118 °C (ethyl acetate/hexanes); IR (KBr): 3334, 1720 cm^{-1}; ^{1}H NMR (500 MHz, $CDCl_3$) δ 2.42 (s, 3H), 4.53 (d, J = 6.0 Hz, 2H), 6.67 (s, 1H), 7.28–7.37 (m, 5H), 7.53 (d, J = 5.0 Hz, 2H), 8.67 (d, J = 5.6 Hz, 2H) ppm; ^{13}C NMR (125 MHz, $CDCl_3$) δ 13.9, 45.2, 120.8, 127.6, 127.7, 128.7, 137.7, 142.3, 150.1, 154.8, 158.3 ppm; HRMS (ESI) calc $C_{15}H_{16}N_3O_2$ [M + H]$^+$ 270.1237; found 270.1235.

(E)-1-(Pyridin-4-yl)ethanone O-phenylcarbamoyl oxime (16).

Reaction time: 2 h (reflux); solvent: chloroform; method of purification: column chromatography (eluent: ethyl acetate/hexanes 2/1); yield: 225 mg (88%); pale yellow crystals, mp 148 °C (ethyl acetate); IR (KBr): 3230, 1756 cm^{-1}; ^{1}H NMR (500 MHz, $CDCl_3$) δ 2.49 (s, 3H), 7.15 (t, J = 7.4 Hz, 1H), 7.37 (t, J = 7.8 Hz, 2H), 7.52 (d, J = 8.1 Hz, 2H), 7.66 (d, J = 5.6 Hz, 2H), 8.16 (br s, 1H), 8.75 (d, J = 5.6 Hz, 2H) ppm; ^{13}C NMR (125 MHz, $CDCl_3$) δ 14.2, 119.7, 121.1, 124.6, 129.2, 136.6, 142.8, 149.7, 151.4, 158.6 ppm; HRMS (ESI) calc $C_{14}H_{14}N_3O_2$ [M + H]$^+$ 256.1081; found 256.1078.

(E)-1-(Pyridin-4-yl)ethanone O-4-methoxyphenylcarbamoyl oxime (17).

Reaction time: 24 h (rt); solvent: tetrahydrofuran; method of purification: column chromatography (eluent: ethyl acetate/hexanes 2/1); yield: 201 mg (70%); yellow crystals, mp 147 °C (ethyl acetate/hexanes); IR (KBr): 3246, 1752 cm^{-1}; ^{1}H NMR (500 MHz, $CDCl_3$) δ 2.47 (s, 3H), 3.80 (s, 3H), 6.89 (d, J = 8.9 Hz, 2H), 7.41 (d, J = 8.8 Hz, 2H), 7.60 (d, J = 6.1 Hz, 2H), 8.09 (br s,

1H), 8.73 (d, J = 5.9 Hz, 2H) ppm; ^{13}C NMR (125 MHz, $CDCl_3$) δ 14.2, 55.5, 114.3, 120.8, 121.9, 129.6, 142.2, 150.3, 152.0, 156.8, 158.7 ppm; HRMS (ESI) calc $C_{15}H_{16}N_3O_3$ $[M + H]^+$ 286.1186; found 286.1189.

(E)-1-(Pyridin-4-yl)ethanone O-4-nitrophenylcarbamoyl oxime (18).

Reaction time: 24 h (reflux); solvent: tetrahydrofuran; method of purification: recrystallization; yield: 291 mg (97%); yellow crystals, mp 169 °C (DMF/H_2O), IR (KBr): 3204, 1786 cm^{-1}; ^1H NMR (500 MHz, DMSO-d_6) δ 2.46 (s, 3H), 7.77 (d, J = 5.0 Hz, 2H), 7.79 (d, J = 9.0 Hz, 2H), 8.26 (d, J = 8.8 Hz, 2H), 8.72 (d, J = 4.8 Hz, 2H), 10.66 (br s, 1H) ppm; ^{13}C NMR (125 MHz, DMSO-d_6) δ 13.6, 118.5, 121.0, 125.1, 141.8, 142.3, 144.8, 150.3, 151.1, 160.4 ppm; HRMS (ESI) calc $C_{14}H_{13}N_4O_4$ $[M + H]^+$ 301.0931; found 301.0931.

(E)-1-(Pyridin-4-yl)ethanone O-4-chlorophenylcarbamoyl oxime (19).

Reaction time: 24 h (reflux); solvent: tetrahydrofuran; method of purification: column chromatography (eluent: ethyl acetate/hexanes 2/1); yield: 212 mg (73%); pale yellow crystals, mp 169 °C (ethyl acetate/hexanes), IR (KBr): 3223, 1760 cm^{-1}; ^1H NMR (500 MHz, $CDCl_3$) δ 2.47 (s, 3H), 7.31 (d, J = 8.8 Hz, 2H), 7.47 (d, J = 8.8 Hz, 2H), 7.59 (d, J = 6.2 Hz, 2H), 8.24 (br s, 1H), 8.73 (d, J = 6.1 Hz, 2H) ppm; ^{13}C NMR (125 MHz, $CDCl_3$) δ 14.3, 120.8, 120.9, 129.2, 129.6, 135.3, 142.0, 150.4, 151.4, 159.2 ppm; HRMS (ESI) calc $C_{14}H_{13}ClN_3O_2$ $[M + H]^+$ 290.0691; found 290.0689, 292.0658 (3:1).

(E)-1-(Pyridin-4-yl)ethanone O-4-fluorophenylcarbamoyl oxime (20).

Reaction time: 2 h (reflux); solvent: tetrahydrofuran; method of purification: column chromatography (eluent: ethyl acetate/hexanes 2/1); yield: 172 mg (63%); pale yellow crystals, mp 181 °C (CH_2Cl_2/hexanes), IR (KBr): 3197, 1752 cm^{-1}; ^1H NMR (500 MHz, $CDCl_3$ + DMSO-d_6) δ 2.40 (s, 3H), 7.03 (t, $^3J_{HF} = {}^3J_{HH}$ = 8.8 Hz, 2H), 7.53 (dd, $^3J_{HH}$ = 8.8 Hz, $^4J_{HF}$ = 4.9 Hz, 2H), 7.72 (d, J = 6.1 Hz, 2H), 8.64 (d, J = 6.1 Hz, 2H), 9.74 (s, 1H) ppm; ^{13}C NMR (125 MHz, $CDCl_3$ + DMSO-d_6) δ 11.7, 113.4 (d, $^2J_{CF}$ = 22.2 Hz), 119.2, 119.4 (d, $^3J_{CF}$ = 6.1 Hz), 132.7 (d, $^4J_{CF}$ = 2.5 Hz), 140.3, 148.3, 150.0, 156.6 (d, $^1J_{CF}$ = 239.5 Hz), 157.0 ppm; HRMS (ESI) calc $C_{14}H_{13}FN_3O_2$ $[M + H]^+$ 274.0986; found 274.0984.

(E)-Isonicotinaldehyde O-benzylcarbamoyl oxime (22).

Reaction time: 24 h (rt); solvent: tetrahydrofuran; method of purification: column chromatography (eluent: CH_2Cl_2/MeOH 20/1); yield: 100 mg (20%); white crystals, mp 101 °C (ethyl acetate); IR (KBr): 3398, 1718 cm^{-1}; ^1H NMR (500 MHz, DMSO-d_6) δ 4.34 (d, J = 6.2 Hz, 2H), 7.27–7.24 (m, 1H), 7.37–7.30 (m, 4H), 7.78 (d, J = 6.0 Hz, 2H), 8.34 (br t, J = 6.0 Hz, 1H), 8.64 (s, 1H), 8.72 (d, J = 6.0 Hz, 2H) ppm; ^{13}C NMR (125 MHz, DMSO-d_6) δ 44.0, 121.8, 126.9, 127.1, 128.3, 137.9, 139.2, 150.4, 152.2, 154.6 ppm; HRMS (ESI) calc $C_{14}H_{14}N_3O_2$ $[M + H]^+$ 256.1081; found 256.1075.

(E)-Isonicotinaldehyde O-phenylcarbamoyl oxime (23).[71]

Reaction time: 24 h (rt); solvent: tetrahydrofuran; method of purification: column chromatography (eluent: CH_2Cl_2/MeOH 20/1); yield: 203 mg (42%); pale yellow crystals, mp 136 °C (MeOH); IR (KBr): 3180, 1750 cm^{-1}; ^1H NMR (500 MHz, DMSO-d_6) δ 7.08 (t, J = 7.6 Hz, 1H), 7.34 (t, J = 7.8 Hz, 2H), 7.54 (d, J = 7.8 Hz, 2H), 7.77 (d, J = 4.9 Hz, 2H), 8.69 (s, 1H), 8.73 (d, J = 5.0 Hz, 2H), 10.01 (s, 1H) ppm; ^{13}C NMR (125 MHz, DMSO-d_6) δ 119.3, 121.8, 123.4, 128.9, 137.8, 138.1, 150.5, 151.4, 153.3 ppm; HRMS (ESI) calc $C_{13}H_{12}N_3O_2$ $[M + H]^+$ 242.0924; found 242.0927.

(E)-Isonicotinaldehyde O-4-methoxyphenylcarbamoyl oxime (24).

Reaction time: 24 h (rt); solvent: tetrahydrofuran; method of purification: column chromatography (eluent: CH_2Cl_2/MeOH 40/1); yield: 191 mg (35%); pale brown-beige crystals, mp

159 °C (ethyl acetate); IR (KBr): 3195, 1763 cm^{-1}; ^{1}H NMR (500 MHz, DMSO-d_6) δ 3.73 (s, 3H), 6.92 (d, J = 8.9 Hz, 2H), 7.43 (d, J = 8.8 Hz, 2H), 7.77 (d, J = 4.6 Hz, 2H), 8.67 (s, 1H), 8.73 (d, J = 4.6 Hz, 2H), 9.79 (s, 1H) ppm; ^{13}C NMR (125 MHz, DMSO-d_6) δ 55.2, 114.1, 121.3, 121.8, 131.0, 137.9, 150.5, 151.6, 153.0, 155.6 ppm; HRMS (ESI) calc $C_{14}H_{14}N_3O_3$ $[M + H]^+$ 272.1030; found 272.1025.

(E)/(Z)-Isonicotinaldehyde O-4-nitrophenylcarbamoyl oxime (25).

Reaction time: 4 h (reflux); solvent: tetrahydrofuran; method of purification: column chromatography (eluent: CH_2Cl_2/MeOH 40/1); yield: 307 mg (54%); yellow crystals (ethyl acetate); IR (KBr): 3210, 1760 cm^{-1}; ^{1}H NMR (500 MHz, DMSO-d_6, mixture of *E*/*Z* isomers *ca.* 9:1) δ 6.59 (d, J = 9.1 Hz, 0.2H), 7.54 (d, J = 5.7 Hz, 0.2H), 7.76 (d, J = 5.6 Hz, 1.8H), 7.79 (d, J = 9.1 Hz, 1.8H), 7.94 (d, J = 9.1 Hz, 0.2H), 8.17 (s, 0.1H), 8.26 (d, J = 9.1 Hz, 1.8H), 8.59 (d, J = 5.6 Hz, 0.2H), 8.74 (s, 0.9H), 8.74 (d, J = 5.6 Hz, 1.8H), 10.74 (br s, 0.9H), 11.80 (br s, 0.1H) ppm; ^{13}C NMR (125 MHz, DMSO-d_6, mixture of *E*/*Z* isomers *ca.* 9:1) δ 112.4, 118.6, 120.6, 121.9, 125.1, 126.4, 137.6, 140.3, 142.3, 144.7, 146.6, 150.1, 150.5, 151.0, 154.2, 155.7, 156.1 ppm; HRMS (ESI) calc $C_{13}H_{11}N_4O_4$ $[M + H]^+$ 287.0775; found 287.0771.

(E)/(Z)-Isonicotinaldehyde O-4-chlorophenylcarbamoyl oxime (26).

Reaction time: 24 h (reflux); solvent: chloroform; method of purification: column chromatography (eluent: ethyl acetate/hexanes 2/1); yield: 447 mg (81%); white crystals (ethyl acetate); IR (KBr): 3247, 1762 cm^{-1}; ^{1}H NMR (500 MHz, DMSO-d_6, mixture of *E*/*Z* isomers *ca.* 9:1) δ 6.54 (d, J = 8.7 Hz, 0.2H), 7.00 (d, J = 8.7 Hz, 0.2H), 7.40 (d, J = 8.8 Hz, 1.8H), 7.54 (d, J = 6.0 Hz, 0.2H), 7.57 (d, J = 8.9 Hz, 1.8H), 7.76 (d, J = 6.0 Hz, 1.8H), 8.17 (s, 0.1H), 8.59 (d, J = 6.0 Hz, 0.2H), 8.70 (s, 0.9H), 8.73 (d, J = 5.9 Hz, 1.8H), 10.15 (s, 0.9H), 11.80 (s, 0.1H) ppm; ^{13}C NMR (125 MHz, DMSO-d_6, mixture of *E*/*Z* isomers *ca.* 9:1) δ 115.2, 120.6, 120.7, 121.8, 127.1, 128.5, 128.8, 137.1, 137.7, 140.3, 146.6, 150.1, 150.5, 151.3, 153.5 ppm; HRMS (ESI) calc $C_{13}H_{11}ClN_3O_2$ $[M + H]^+$ 276.0534; found 276.0529, 278.0501 (3:1).

(E)/(Z)-Isonicotinaldehyde O-4-fluorophenylcarbamoyl oxime (27).

Reaction time: 24 h (rt); solvent: tetrahydrofuran; method of purification: column chromatography (eluent: CH_2Cl_2/MeOH 40/1); yield: 120 mg (23%); pale yellow crystals, (ethyl acetate/1,4-dioxane); IR (KBr): 3218, 1763 cm^{-1}; ^{1}H NMR (500 MHz, DMSO-d_6, mixture of *E*/*Z* isomers *ca.* 9:1) δ 7.11 (t, $^{3}J_{HF}$ = $^{3}J_{HH}$ = 8.9 Hz, 0.2H), 7.19 (t, $^{3}J_{HF}$ = $^{3}J_{HH}$ = 8.8 Hz, 1.8H), 7.45 (dd, $^{4}J_{HF}$ = 8.9 Hz, $^{3}J_{HH}$ = 5.0 Hz, 0.2H), 7.55 (d, J = 5.8 Hz, 0.2H, obscured), 7.55 (dd, J = 9.1, 5.0 Hz, 1.8H), 7.77 (d, J = 5.7 Hz, 1.8H), 8.17 (s, 0.1H), 8.59 (d, J = 5.8 Hz, 0.2H), 8.69 (s, 0.9H), 8.73 (d, J = 5.7 Hz, 1.8H), 10.03 (s, 0.9H), 11.80 (s, 0.1H) ppm; ^{13}C NMR (125 MHz, DMSO-d_6, mixture of *E*/*Z* isomers *ca.* 9:1) δ 115.2 (d, $^{2}J_{CF}$ = 22.1 Hz, minor isomer), 115.5 (d, $^{2}J_{CF}$ = 22.3 Hz, major isomer), 120.0 (d, $^{3}J_{CF}$ = 7.6 Hz, minor isomer), 120.6, 121.2 (d, $^{3}J_{CF}$ = 7.6 Hz, major isomer), 121.8, 134.4 (d, $^{4}J_{CF}$ = 2.4 Hz, major isomer), 136.0 (d, $^{4}J_{CF}$ = 2.2 Hz, minor isomer), 137.8, 140.3, 146.6, 150.1, 150.5, 151.5, 153.3, 157.3 (d, $^{1}J_{CF}$ = 236.8 Hz, minor isomer), 158.2 (d, $^{1}J_{CF}$ = 238.5 Hz, major isomer) ppm; HRMS (ESI) calc $C_{13}H_{11}FN_3O_2$ $[M + H]^+$ 260.0830; found 260.0833.

2. *^{1}H NMR and ^{13}C NMR of amidoxime, ethanone oxime and aldoxime carbamates*

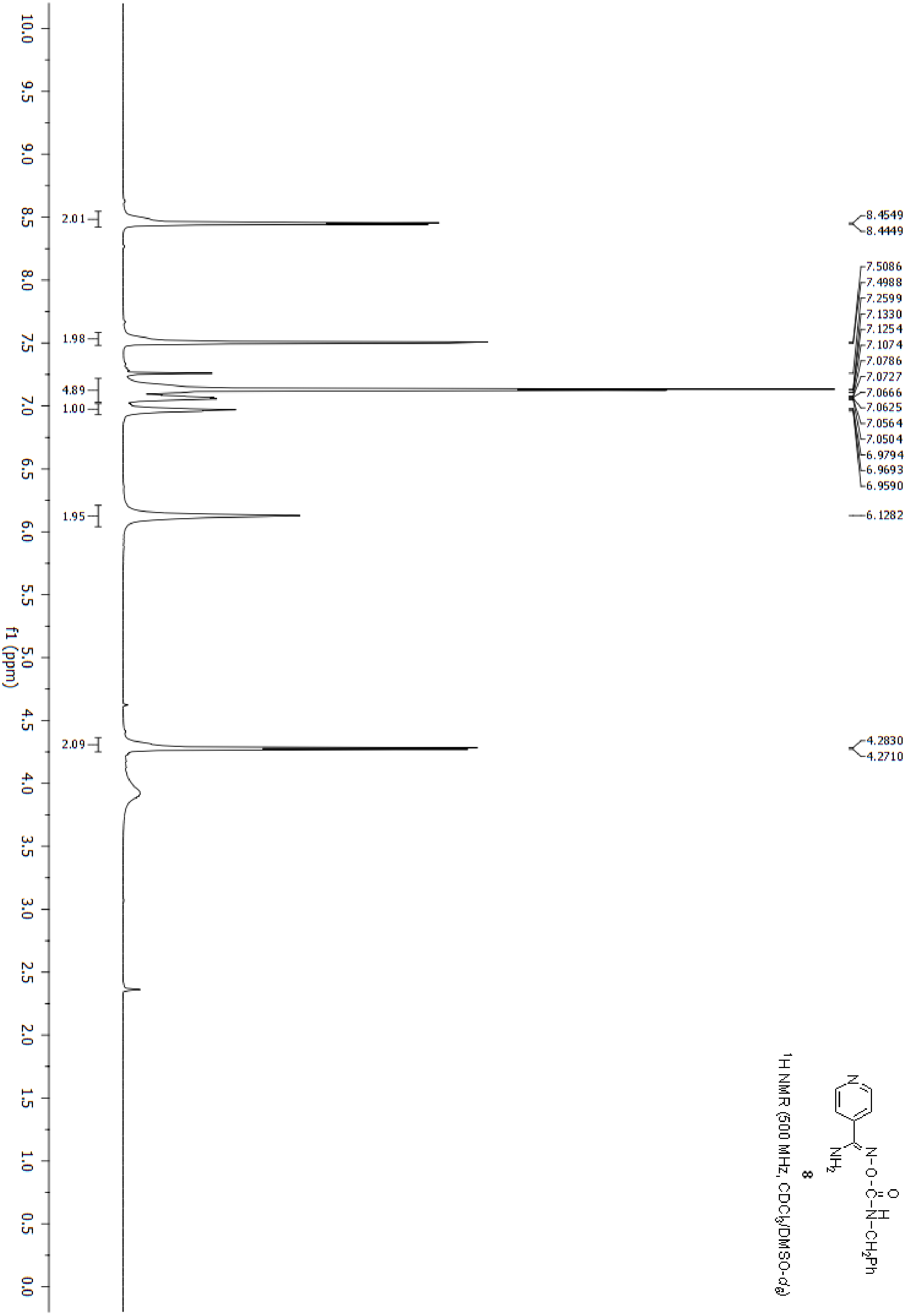

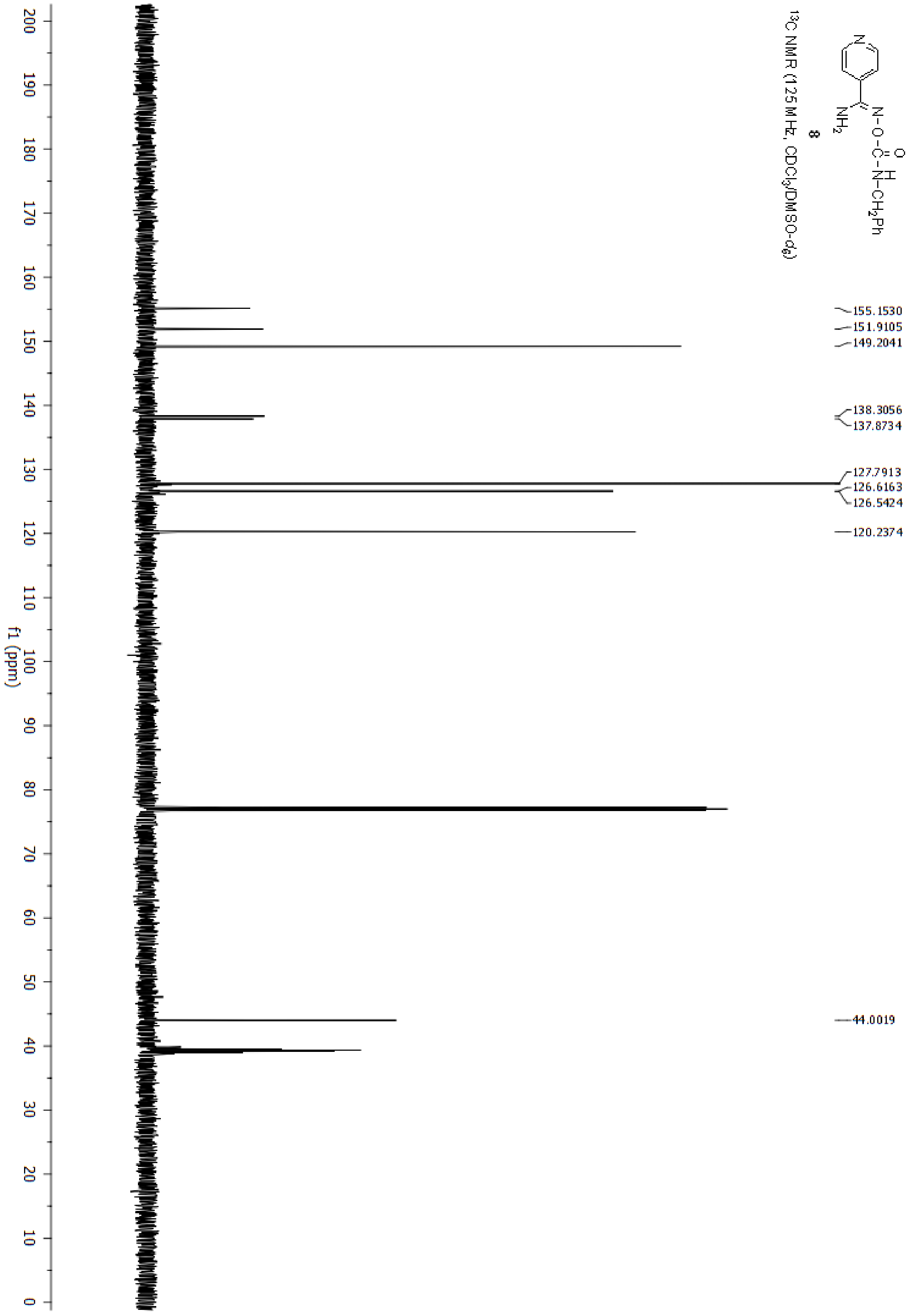
8
13C NMR (125 MHz, CDCl3/DMSO-d6)
155.1530
151.9105
149.2041
138.3056
137.8734
127.7913
126.6163
126.5424
120.2374
44.0019
f1 (ppm)

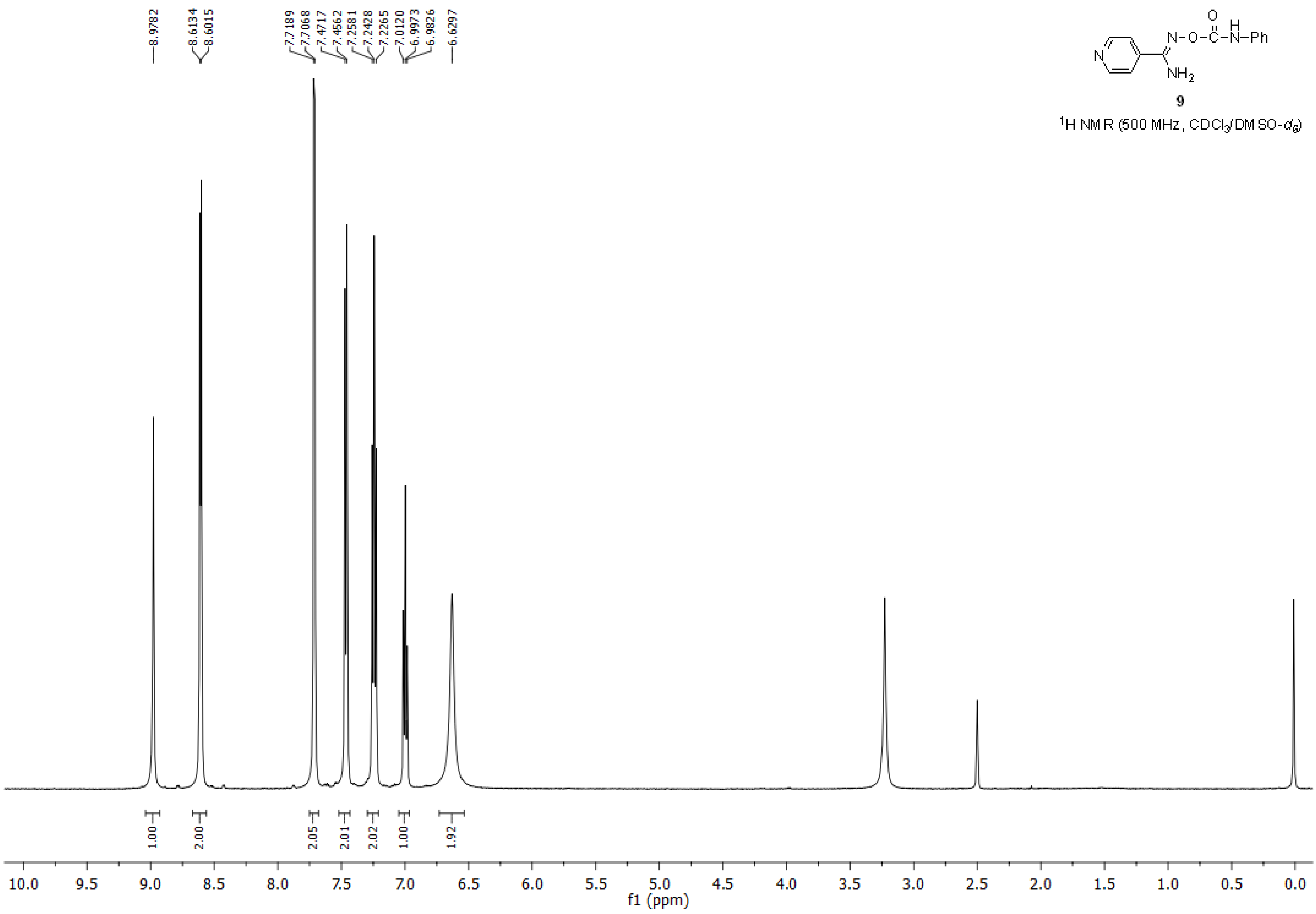
8.9782
8.6134
8.6015
7.7189
7.7068
7.4717
7.4562
7.2581
7.2428
7.2265
7.0120
6.9973
6.9826
6.6297
N-O-C(=O)-N(H)-Ph
NH2
9
1H NMR (500 MHz, CDCl3/DMSO-d6)
1.00
2.00
2.05
2.01
2.02
1.00
1.92
10.0 9.5 9.0 8.5 8.0 7.5 7.0 6.5 6.0 5.5 5.0 4.5 4.0 3.5 3.0 2.5 2.0 1.5 1.0 0.5 0.0
f1 (ppm)

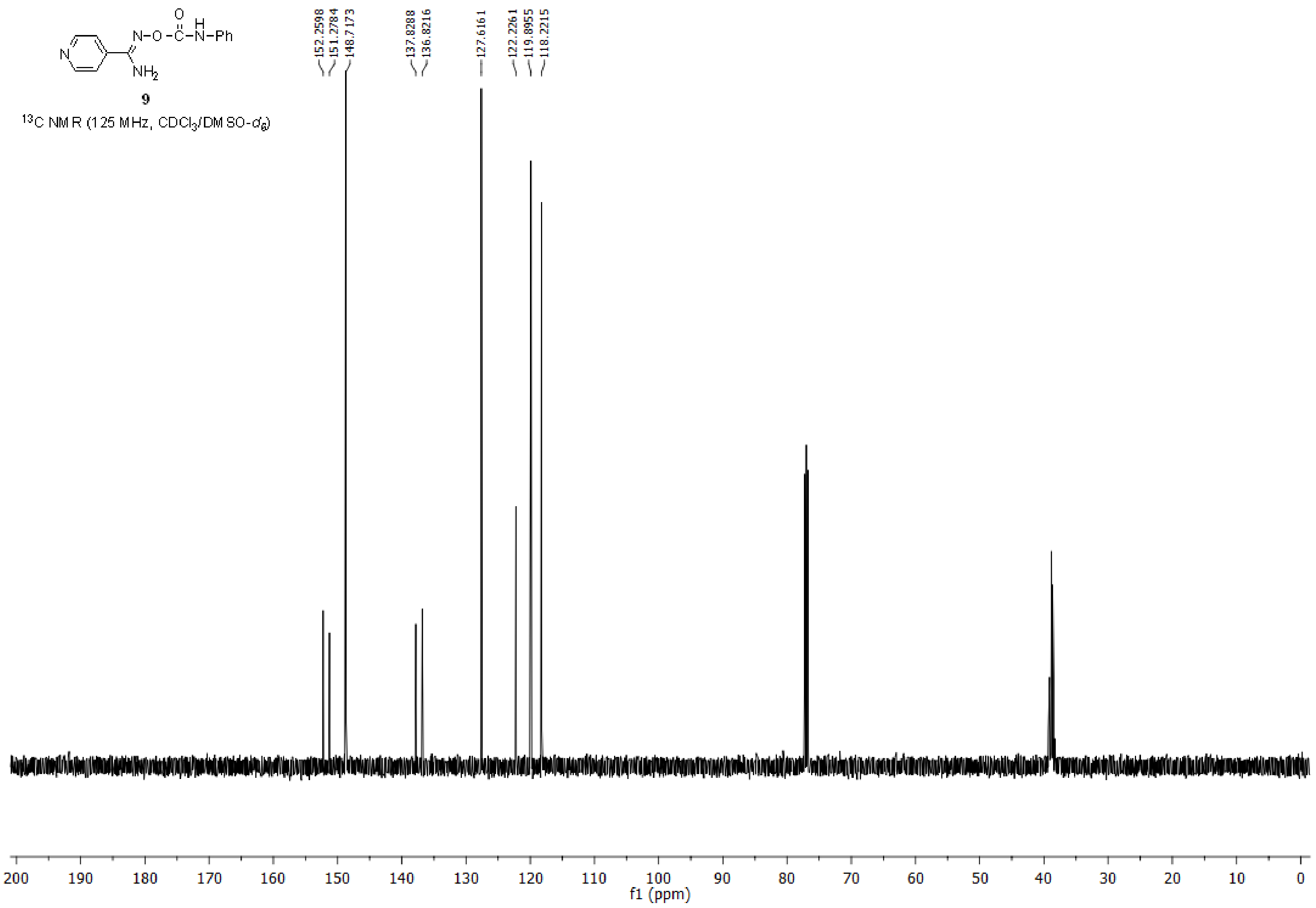

9

^{13}C NMR (125 MHz, $CDCl_3$/DMSO-d_6)

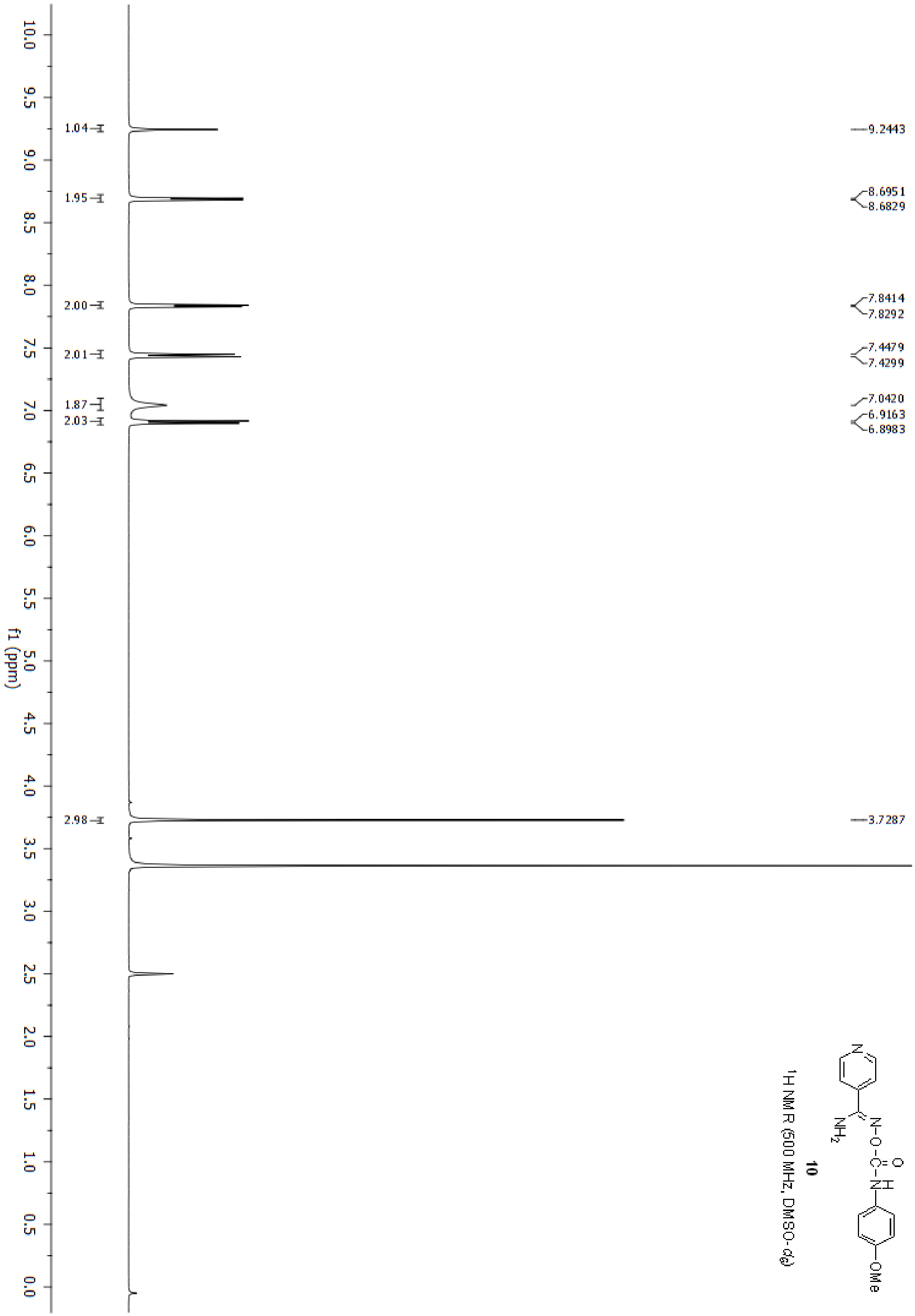

10
¹H NMR (500 MHz, DMSO-d6)
OMe
NH2
9.2443
8.6951
8.6829
7.8414
7.8292
7.4479
7.4299
7.0420
6.9163
6.8983
3.7287
1.04
1.95
2.00
2.01
1.87
2.03
2.98
f1 (ppm)

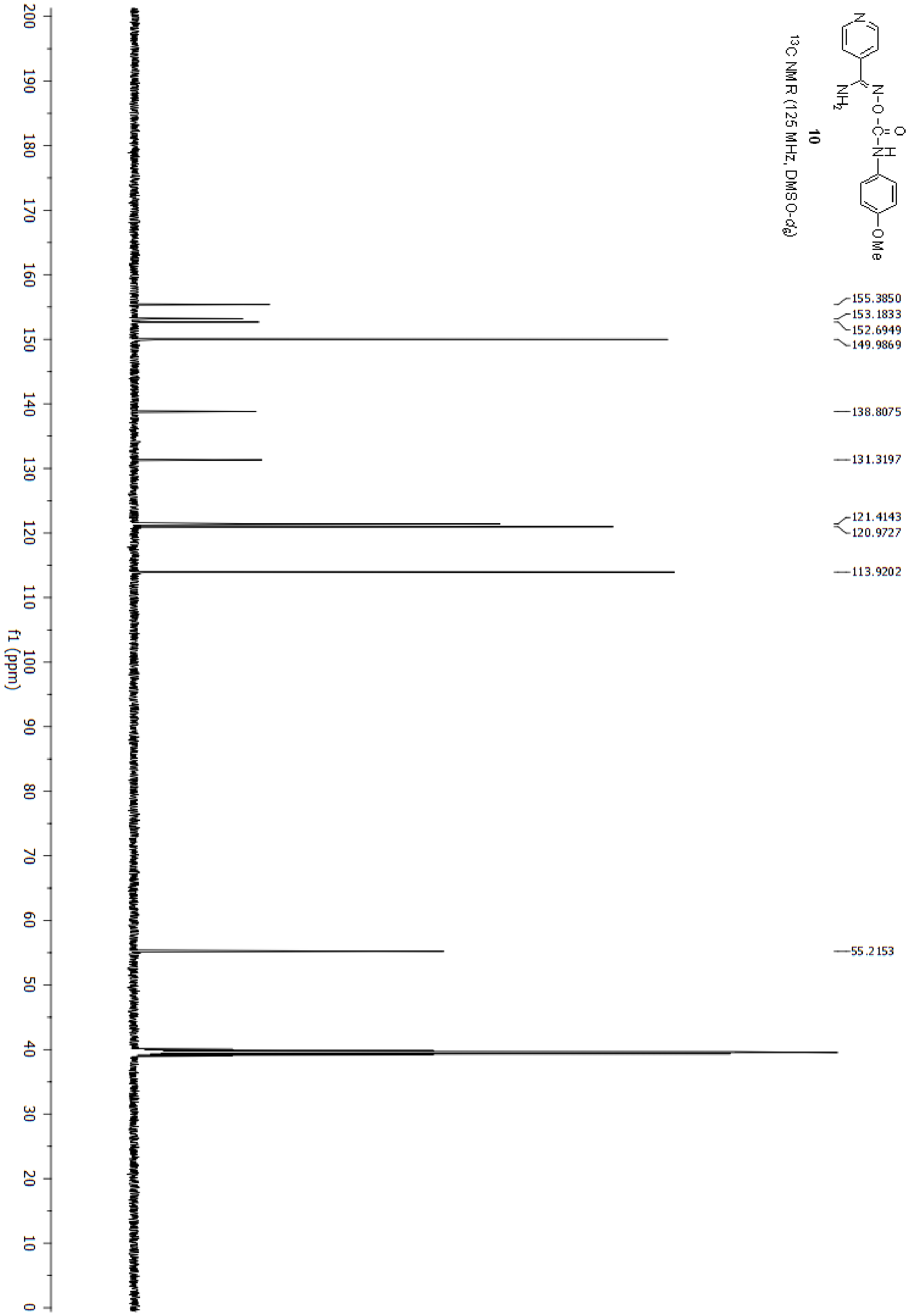
10
13C NMR (125 MHz, DMSO-d6)
155.3850
153.1833
152.6949
149.9869
138.8075
131.3197
121.4143
120.9727
113.9202
55.2153
f1 (ppm)

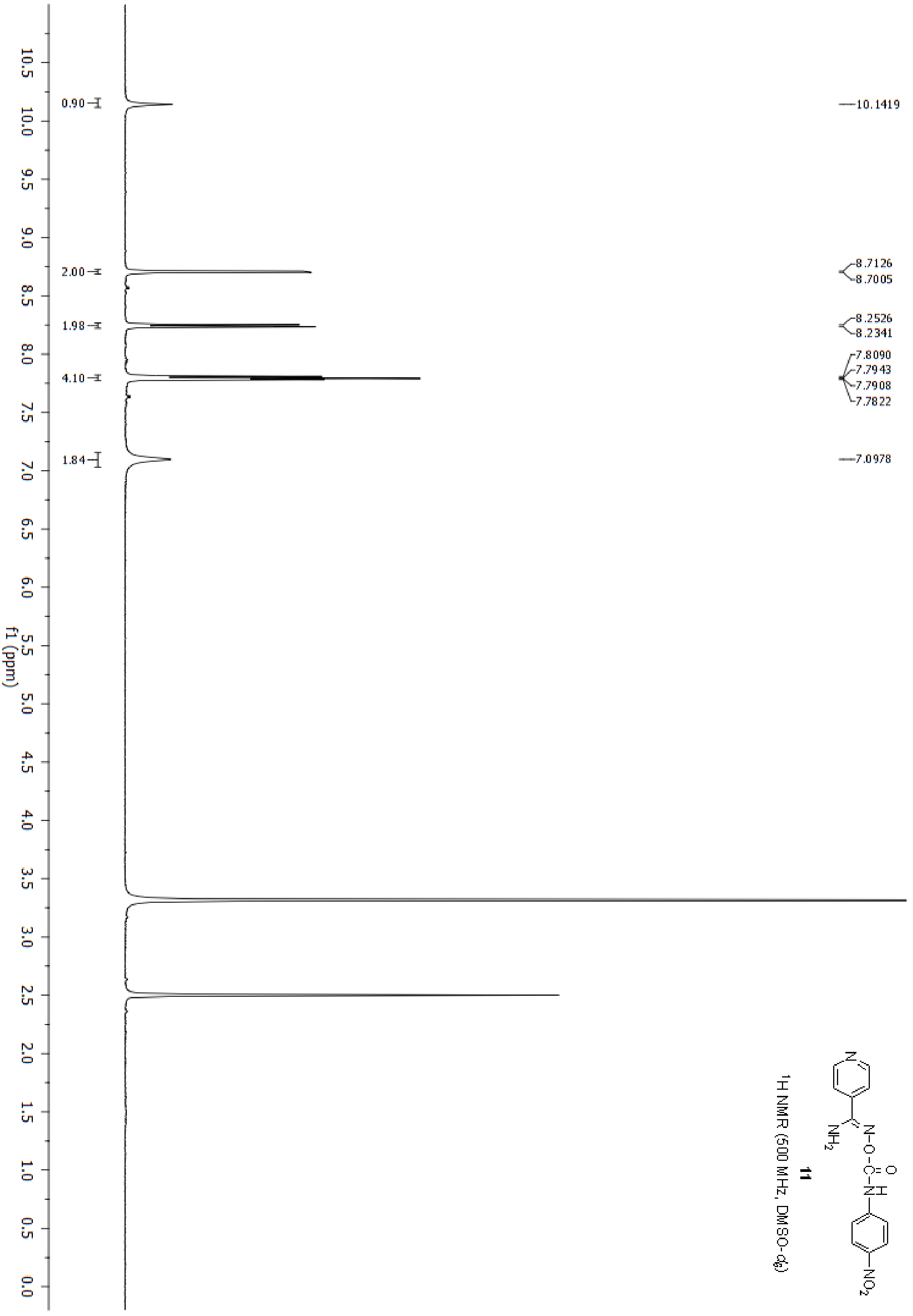

11

^{1}H NMR (500 MHz, DMSO-d_6)

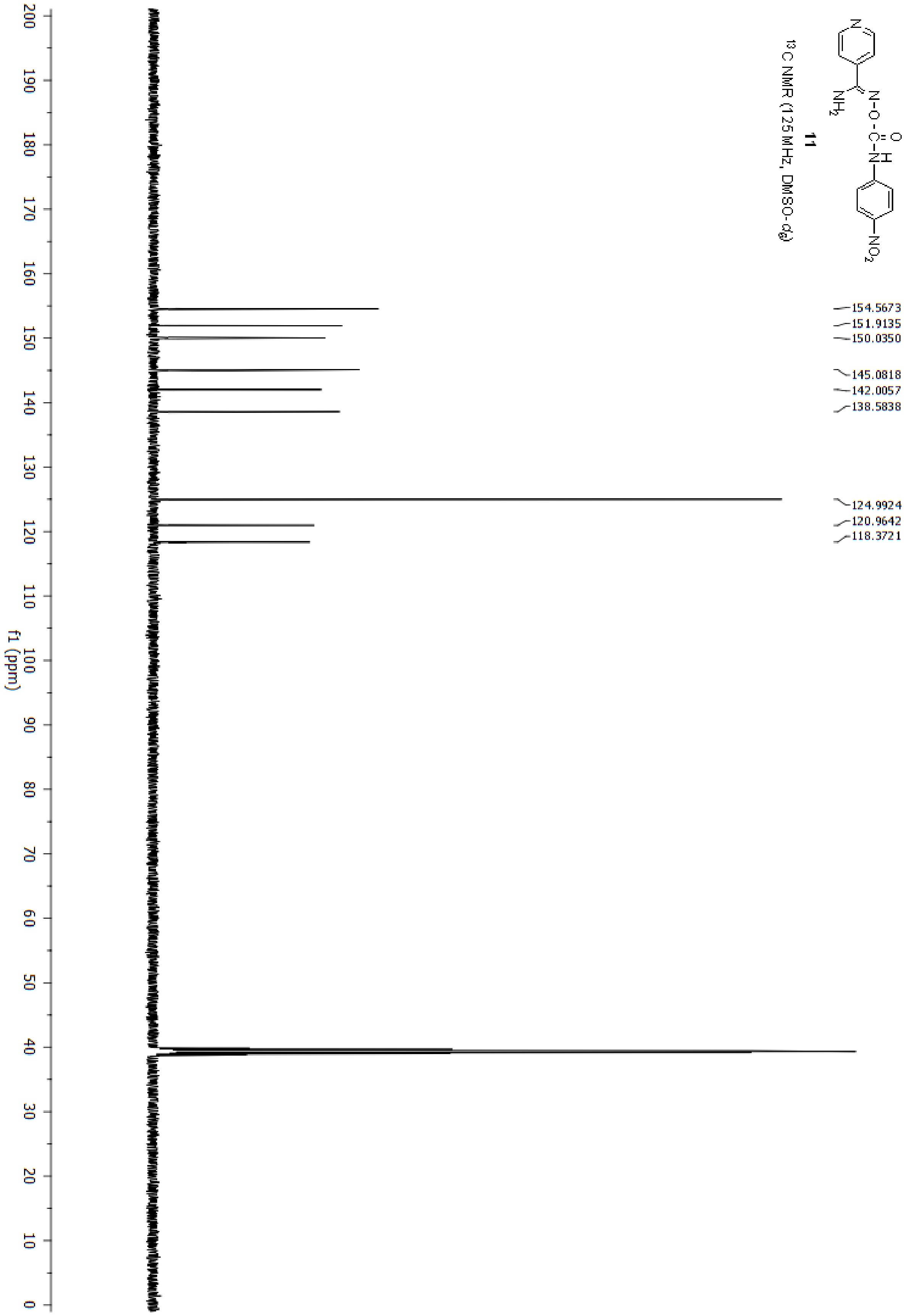
NH2
N-O-C-N
O
H
NO2
11
13C NMR (125 MHz, DMSO-d6)
154.5673
151.9135
150.0350
145.0818
142.0057
138.5838
124.9924
120.9642
118.3721
200
190
180
170
160
150
140
130
120
110
100
90
80
70
60
50
40
30
20
10
0
f1 (ppm)

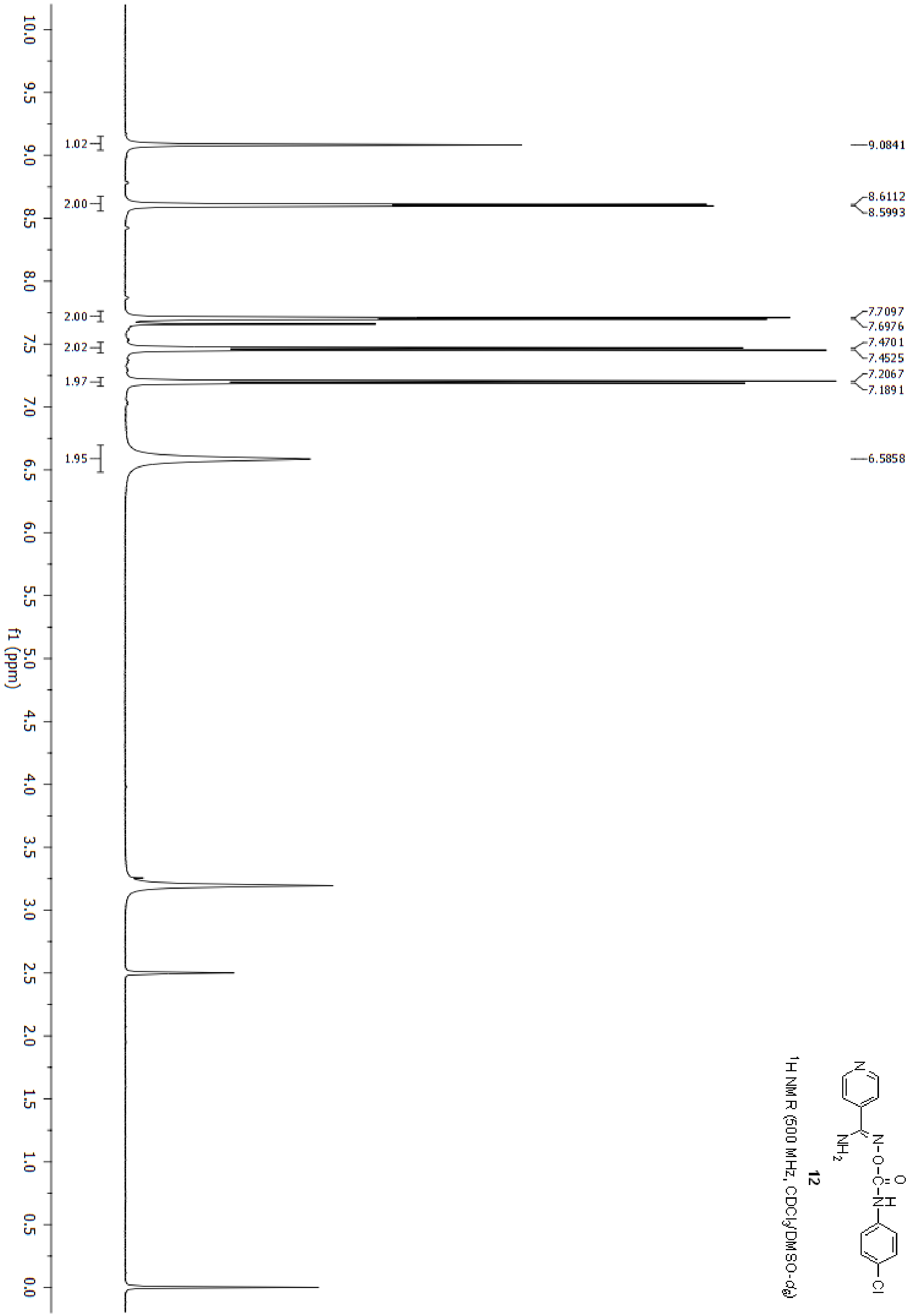

12

^{1}H NMR (500 MHz, $CDCl_3$/DMSO-d_6)

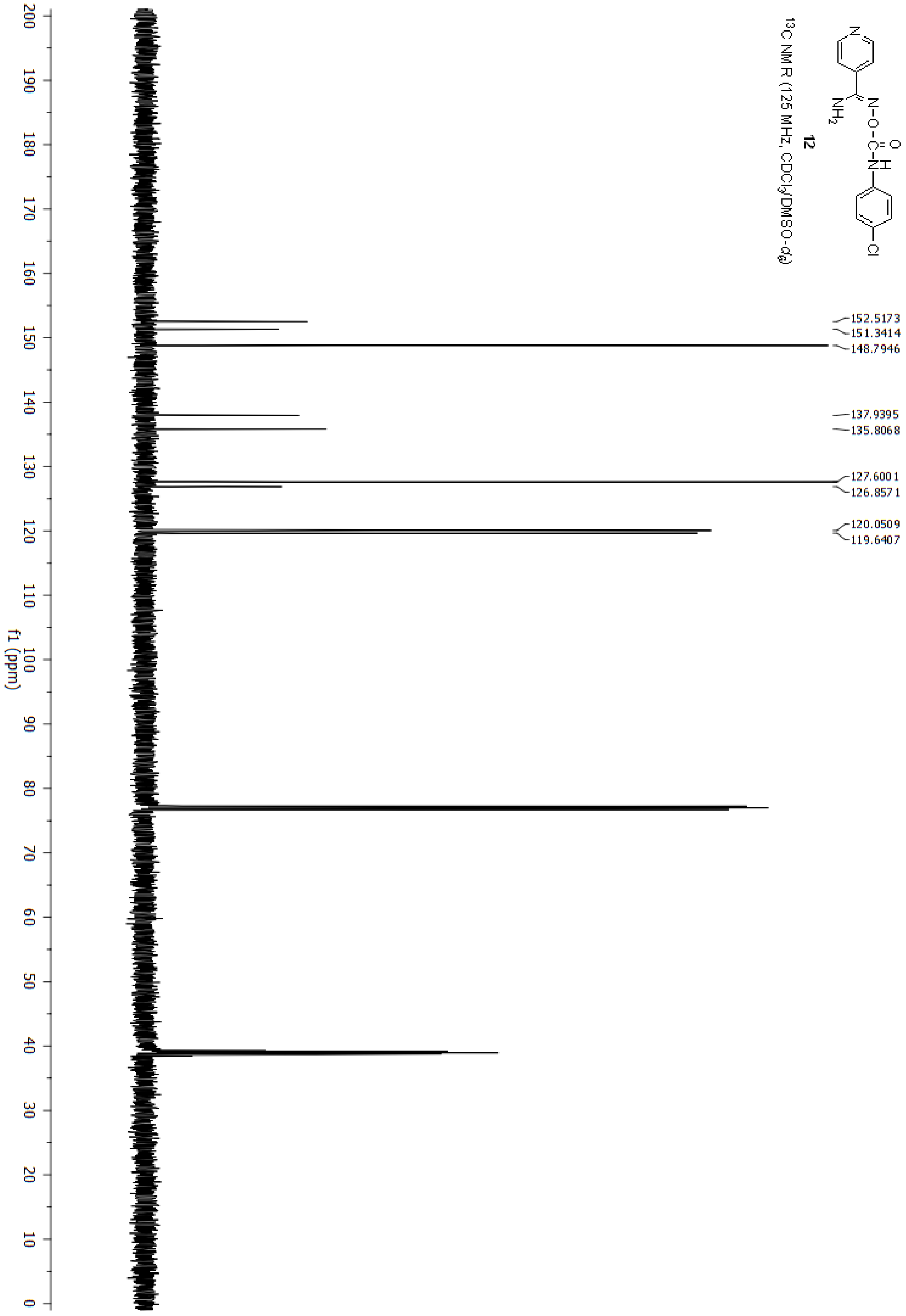
12
13C NMR (125 MHz, CDCl3/DMSO-d6)
152.5173
151.3414
148.7946
137.9395
135.8068
127.6001
126.8571
120.0509
119.6407
f1 (ppm)
200
190
180
170
160
150
140
130
120
110
100
90
80
70
60
50
40
30
20
10
0

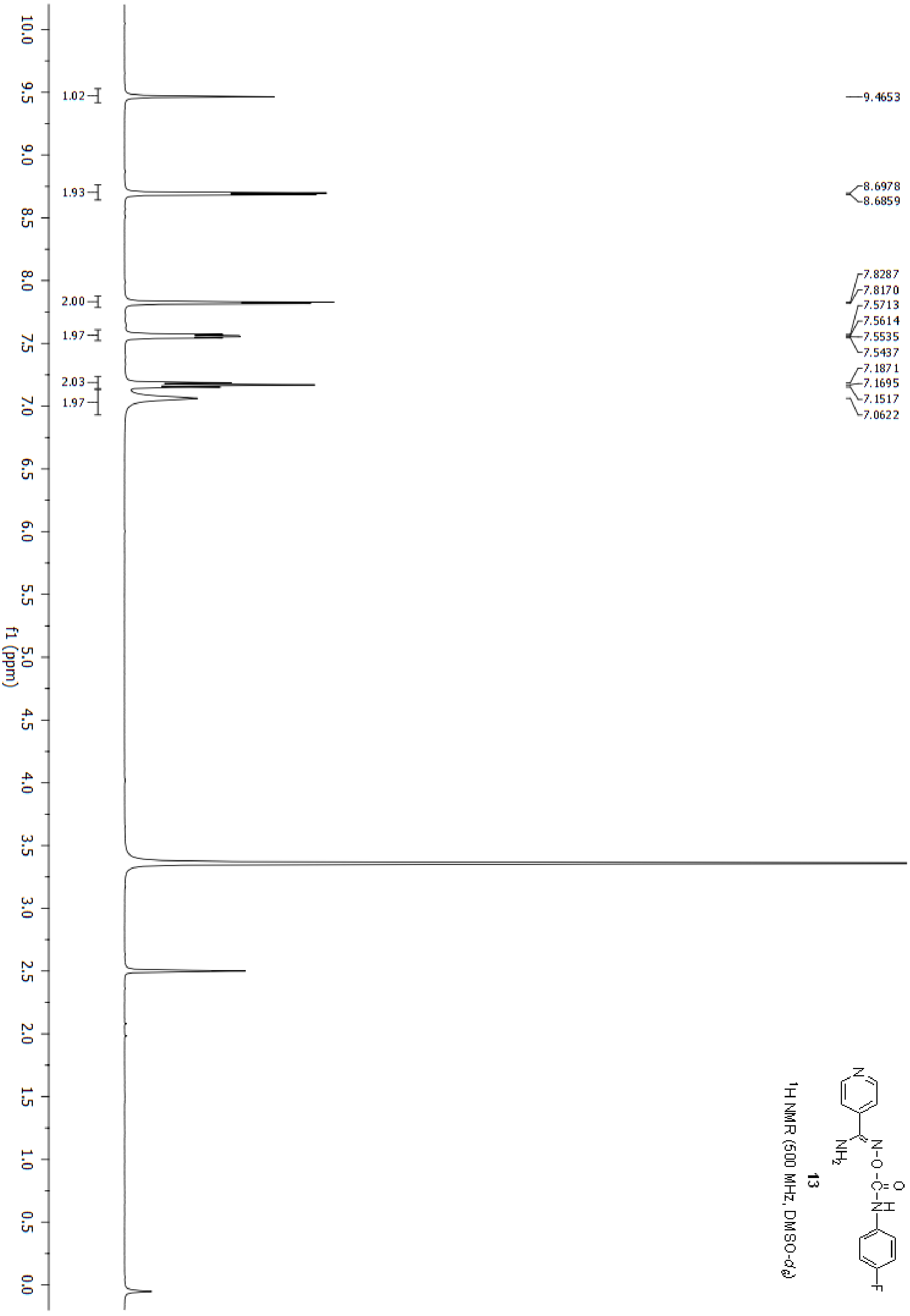

13

^{1}H NMR (500 MHz, DMSO-d_6)

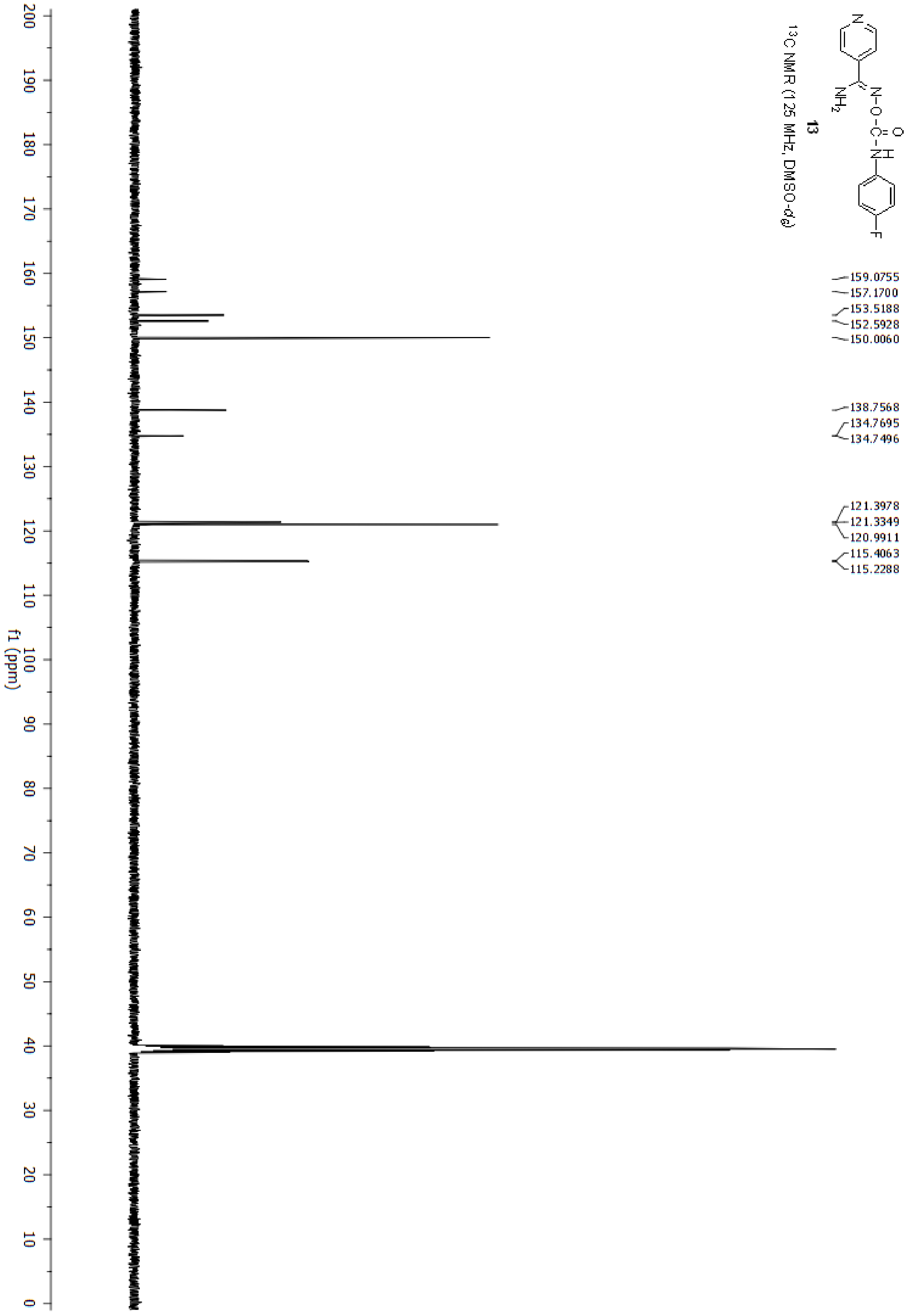
13
13C NMR (125 MHz, DMSO-d6)
159.0755
157.1700
153.5188
152.5928
150.0060
138.7568
134.7695
134.7496
121.3978
121.3349
120.9911
115.4063
115.2288
f1 (ppm)
200
190
180
170
160
150
140
130
120
110
100
90
80
70
60
50
40
30
20
10
0

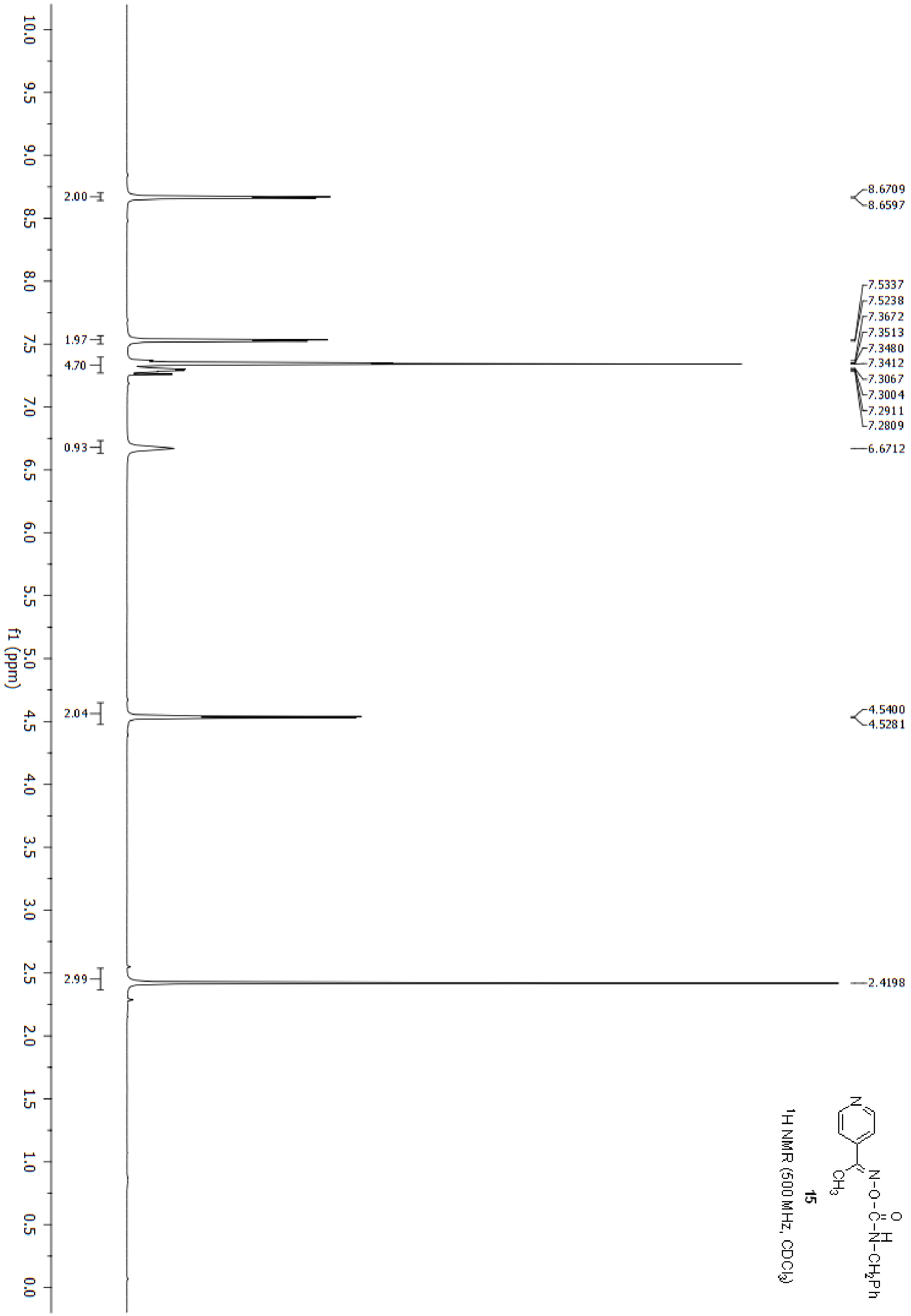
8.6709
8.6597
7.5337
7.5238
7.3672
7.3513
7.3480
7.3412
7.3067
7.3004
7.2911
7.2809
6.6712
4.5400
4.5281
2.4198
2.00
1.97
4.70
0.93
2.04
2.99
f1 (ppm)
15
¹H NMR (500 MHz, CDCl3)

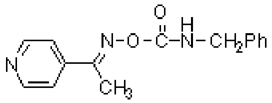

15

^{13}C NMR (125 MHz, $CDCl_3$)

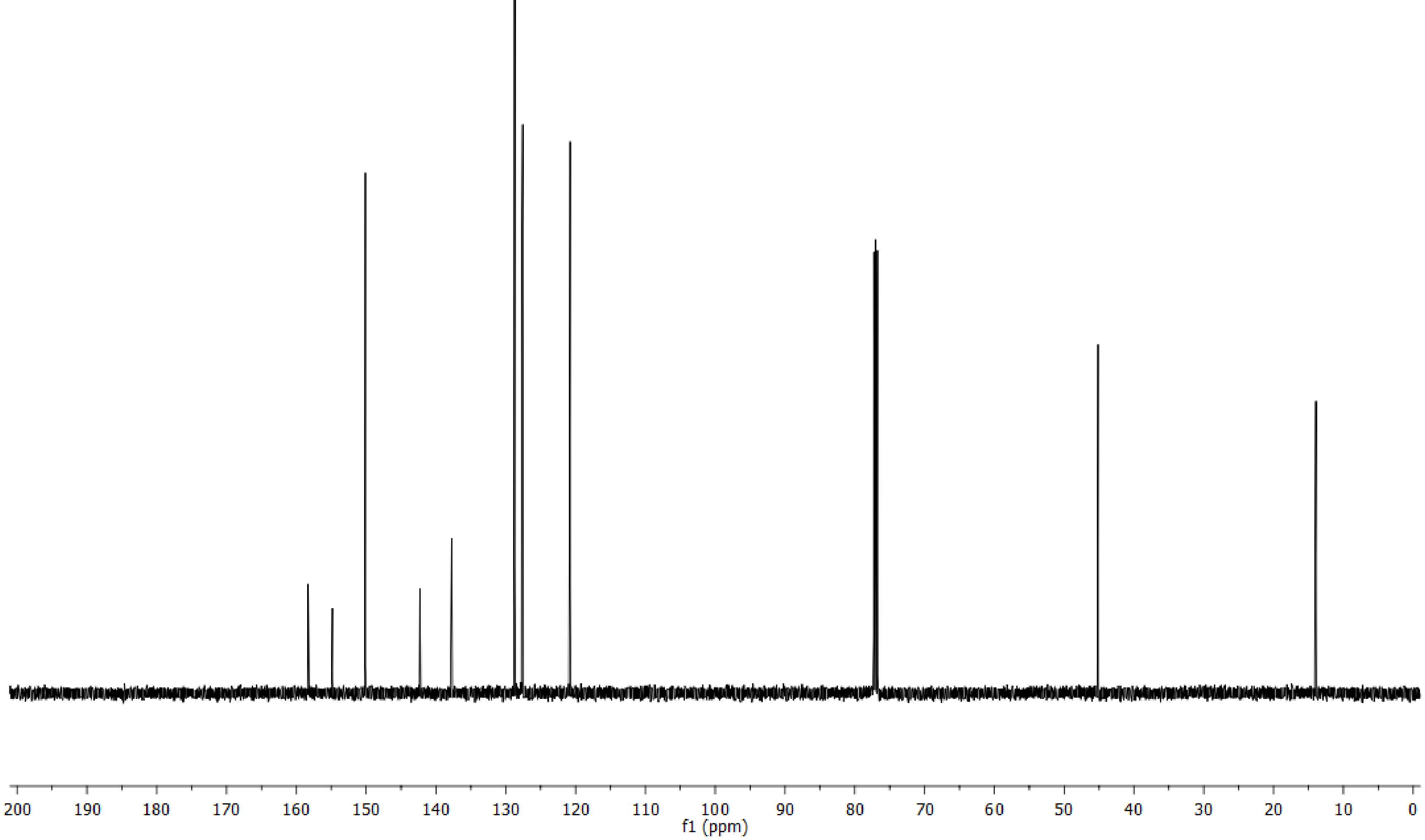

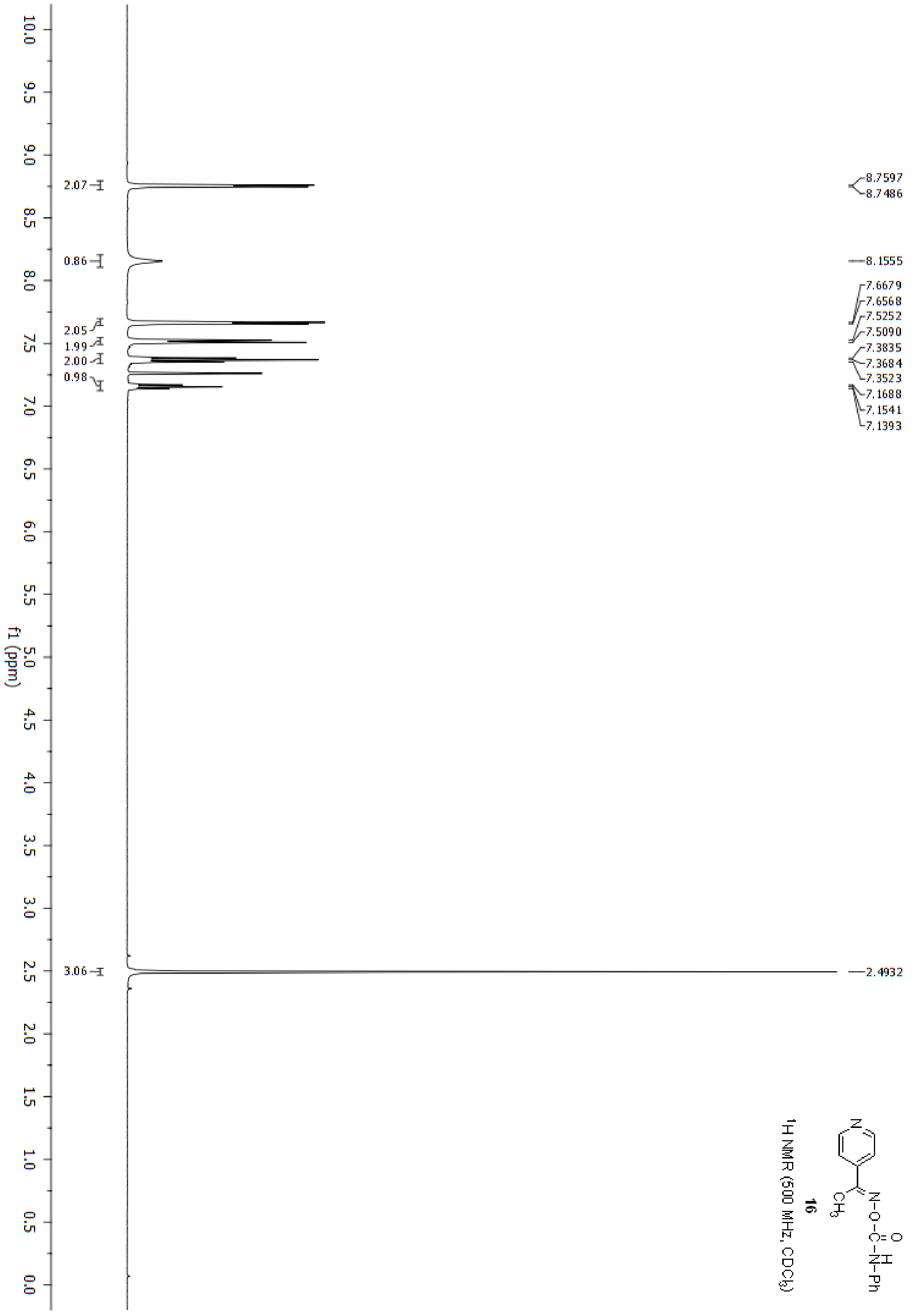

8.7597
8.7486
8.1555
7.6679
7.6568
7.5252
7.5090
7.3835
7.3684
7.3523
7.1688
7.1541
7.1393
2.4932
2.07
0.86
2.05
1.99
2.00
0.98
3.06
f1 (ppm)
16
1H NMR (500 MHz, CDCl3)

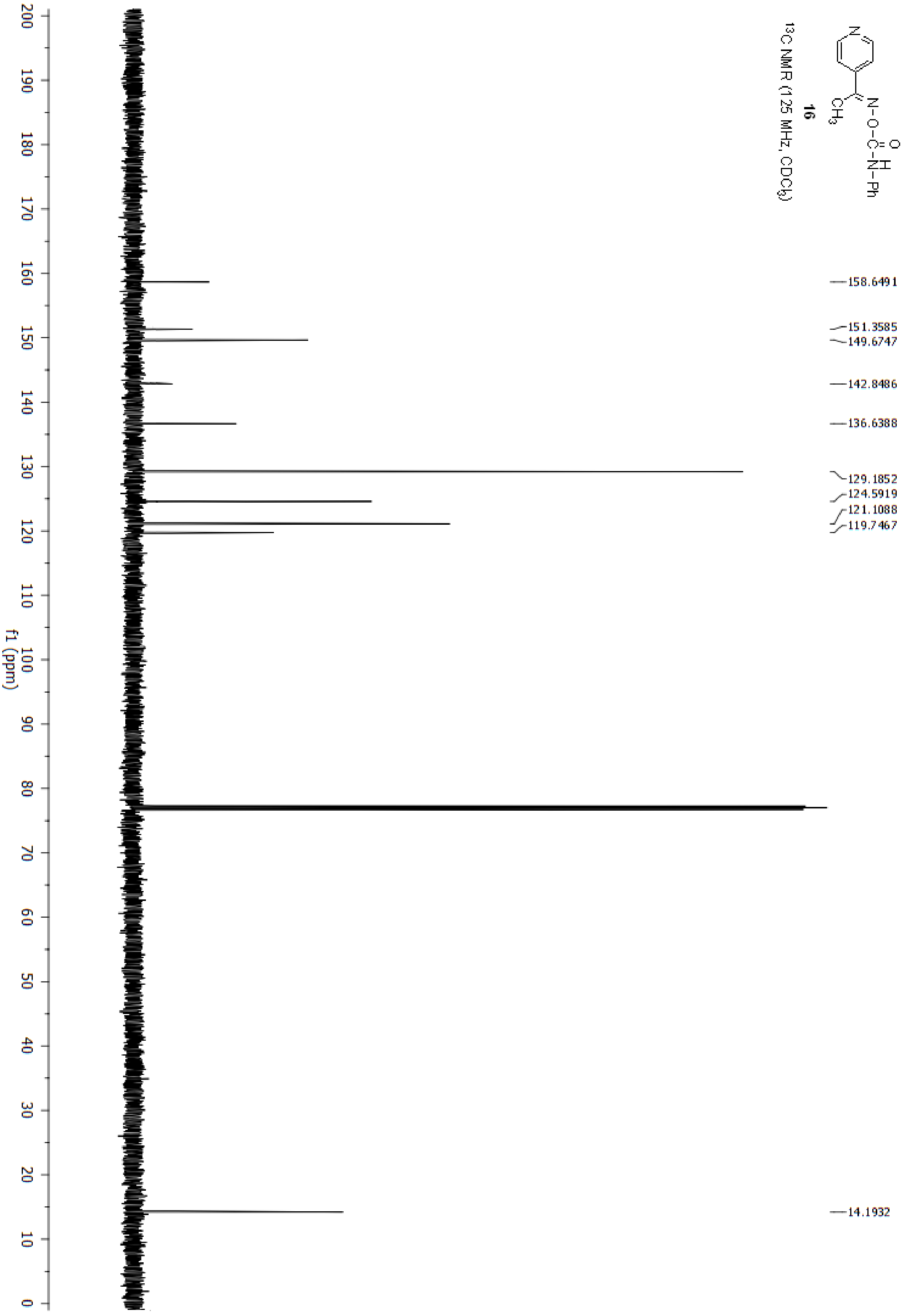
16
13C NMR (125 MHz, CDCl3)
158.6491
151.3585
149.6747
142.8486
136.6388
129.1852
124.5919
121.1088
119.7467
14.1932
f1 (ppm)
200
190
180
170
160
150
140
130
120
110
100
90
80
70
60
50
40
30
20
10
0

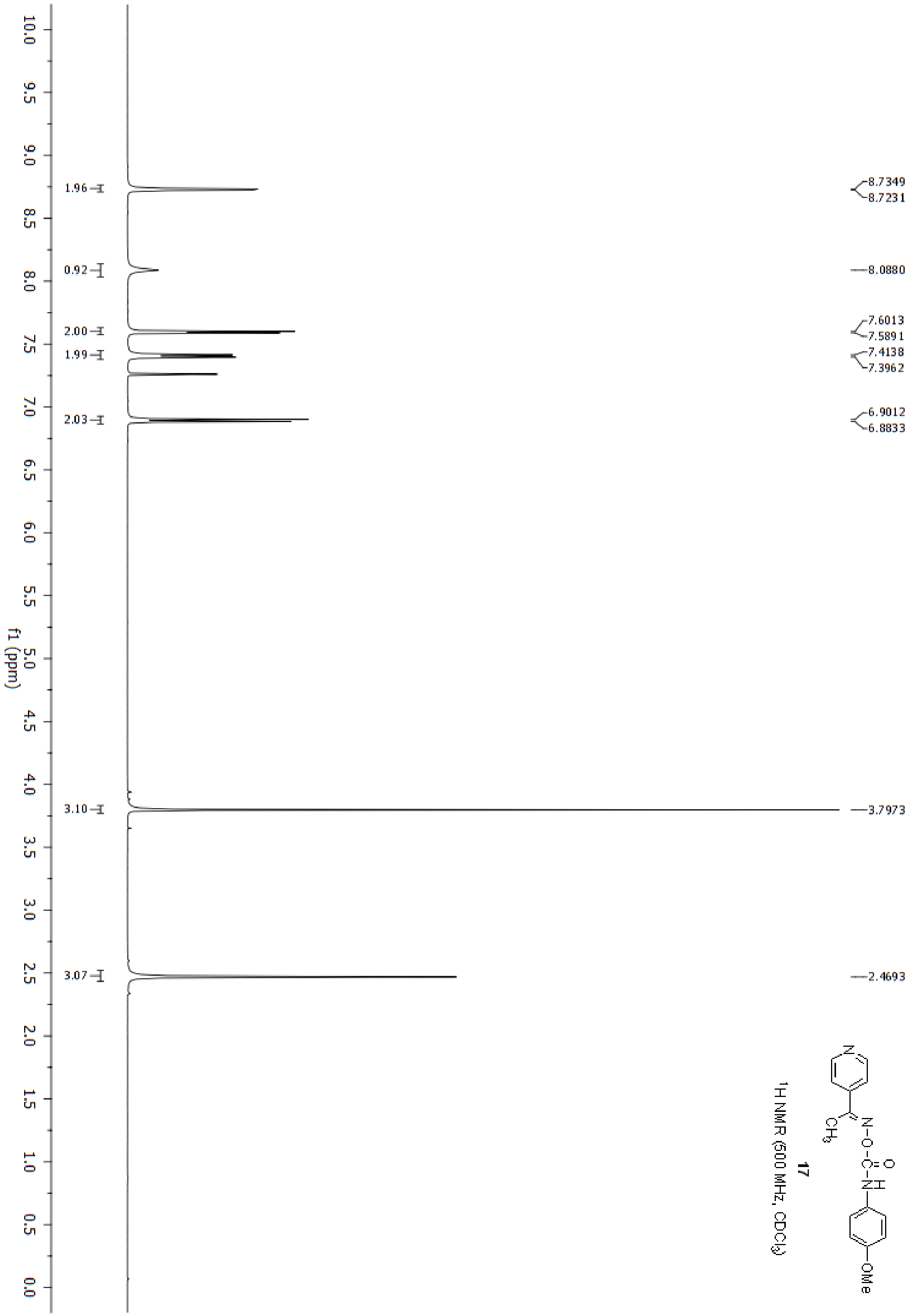

17

^1H NMR (500 MHz, $CDCl_3$)

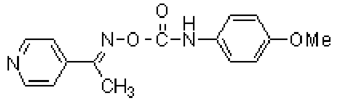

17

^{13}C NMR (125 MHz, $CDCl_3$)

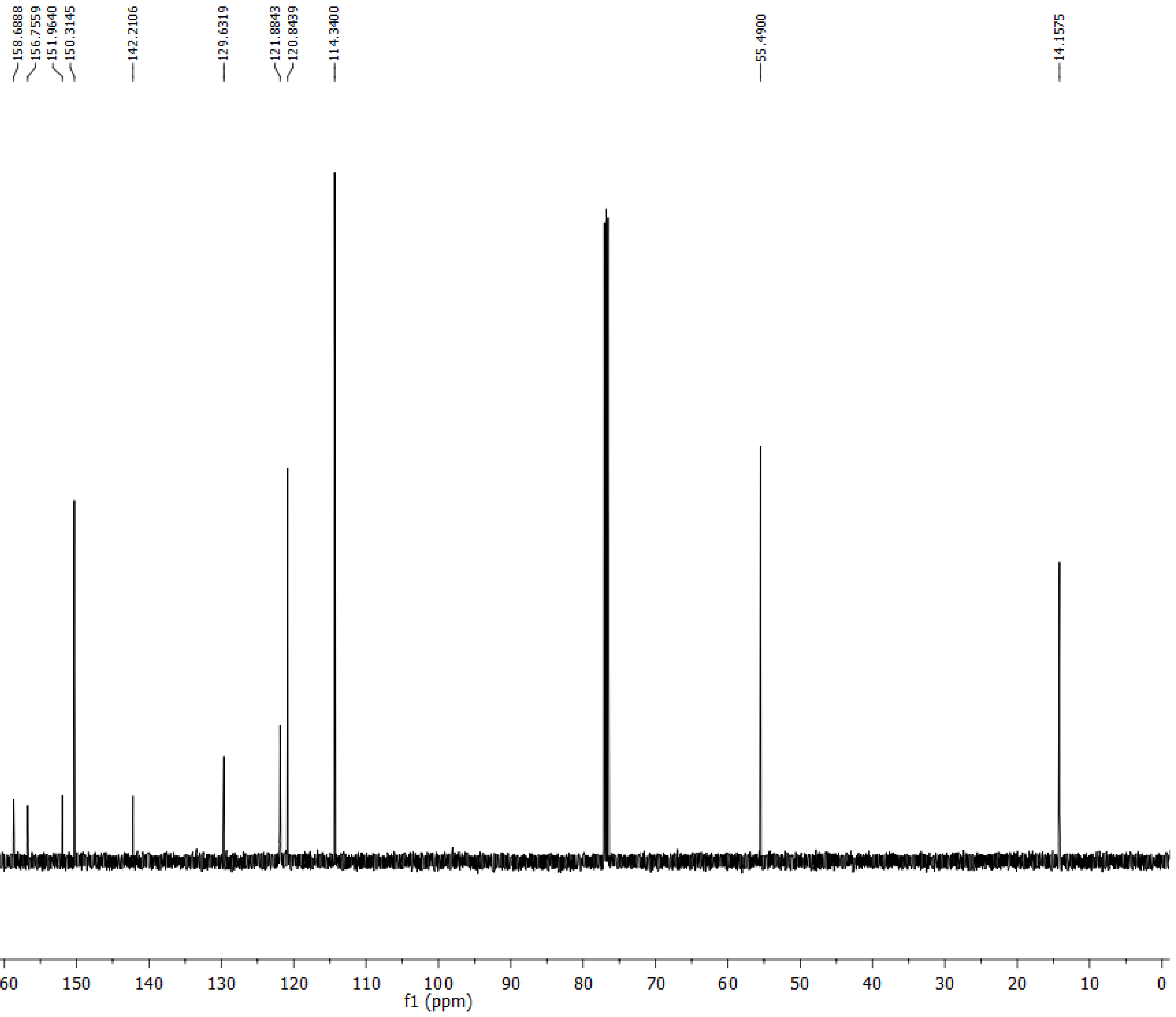

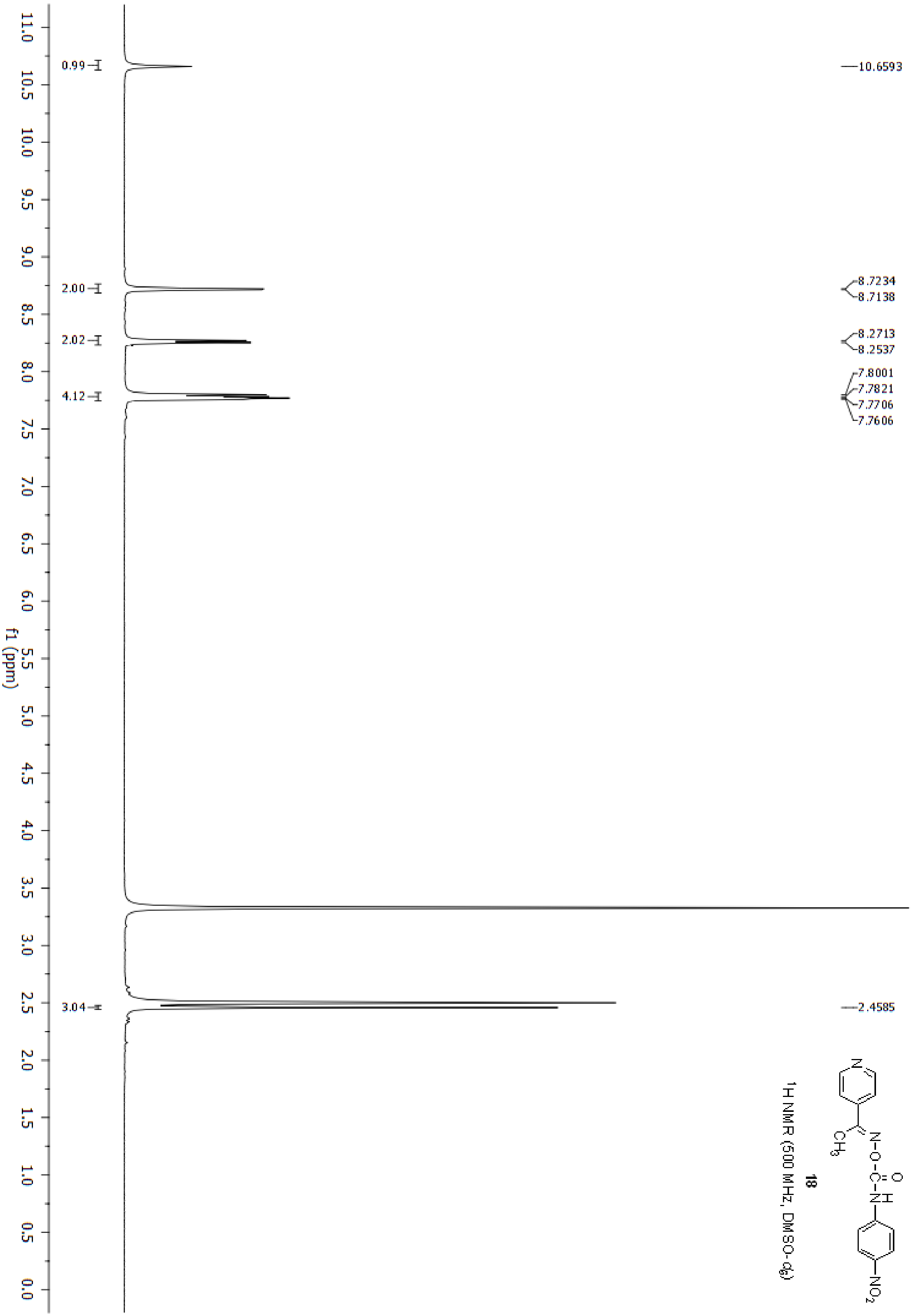

10.6593
8.7234
8.7138
8.2713
8.2537
7.8001
7.7821
7.7706
7.7606
2.4585
0.99
2.00
2.02
4.12
3.04
f1 (ppm)
18
1H NMR (500 MHz, DMSO-d6)

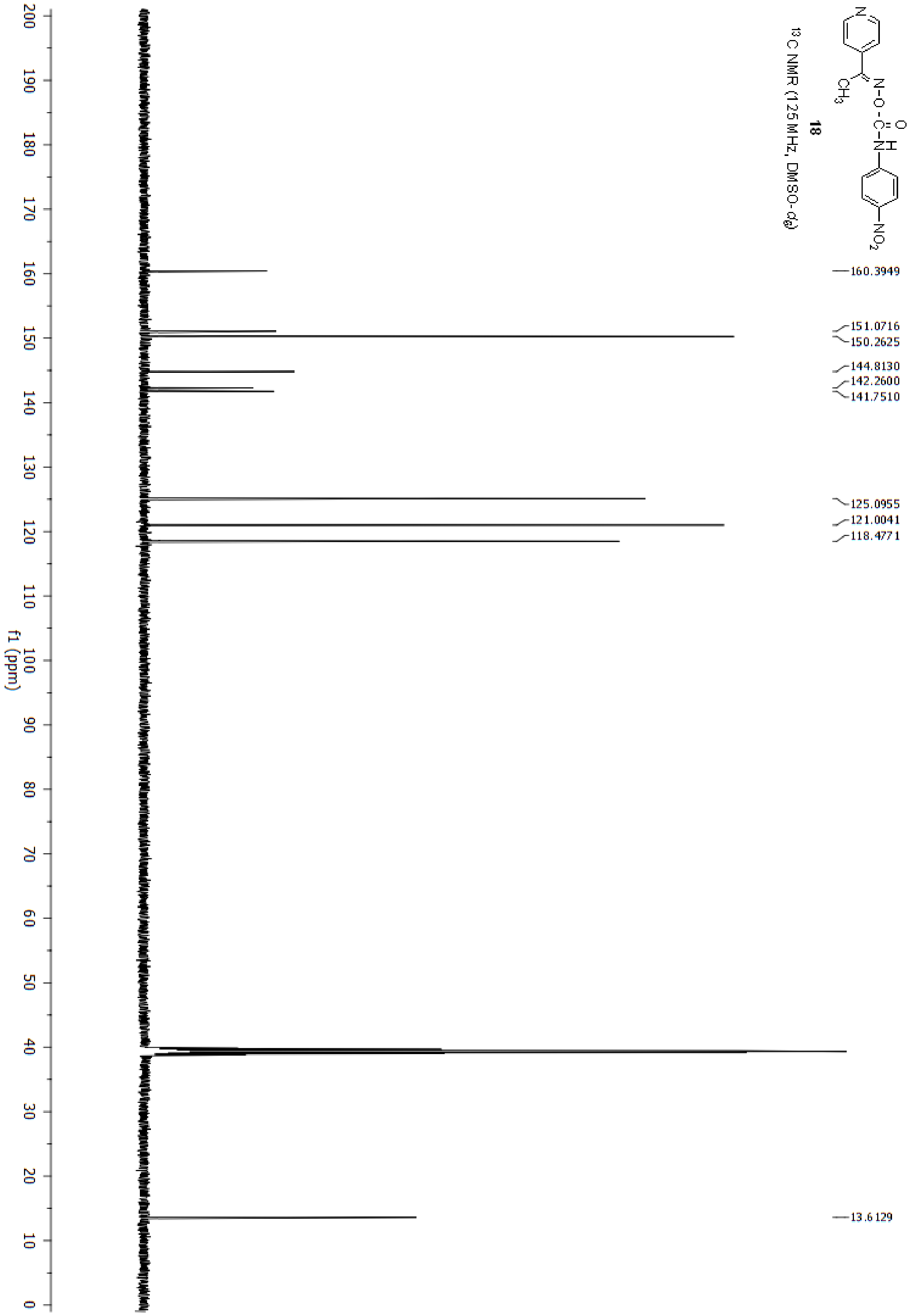

18

^{13}C NMR (125 MHz, DMSO-d_6)

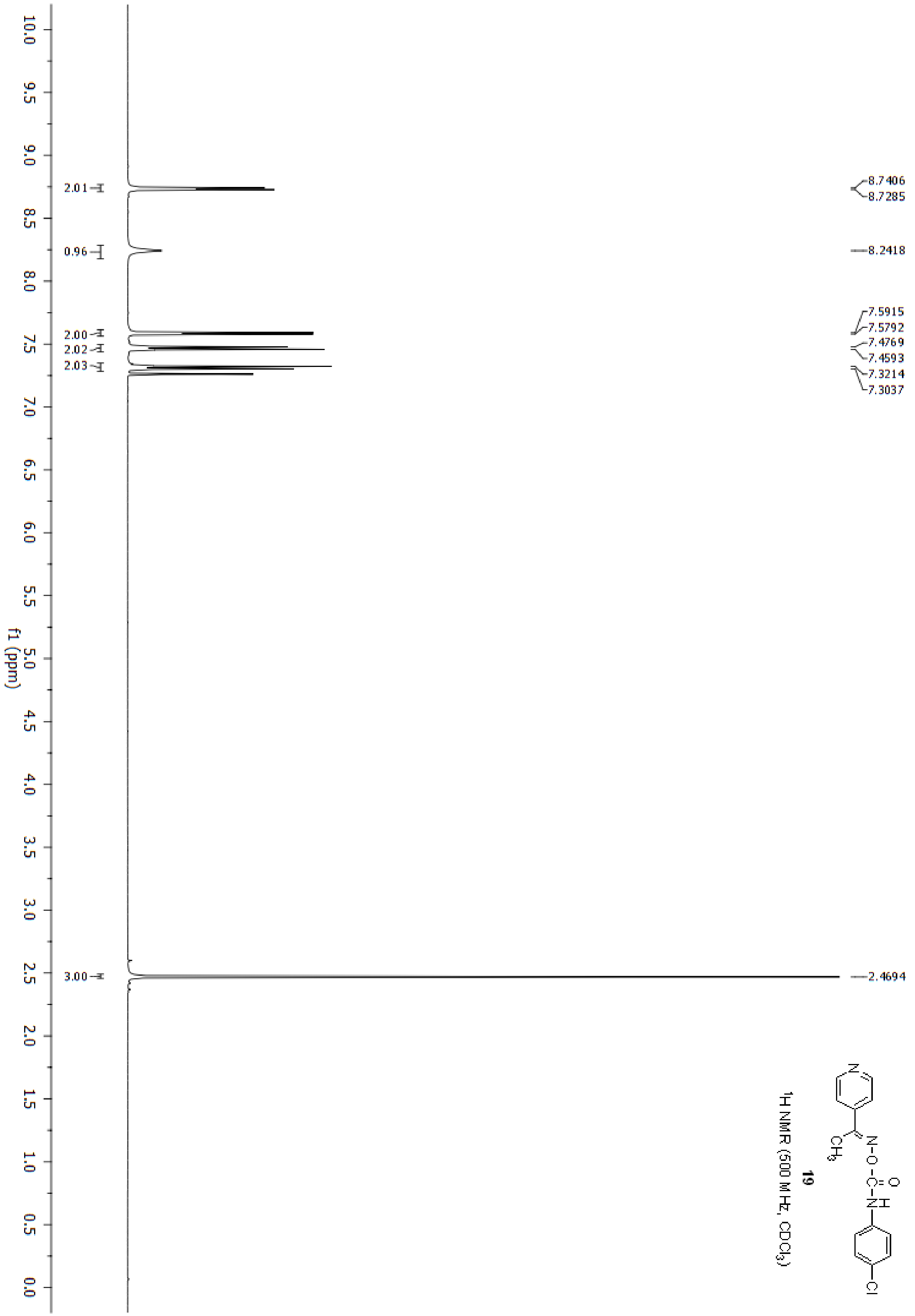

10.0
9.5
9.0
8.5
8.0
7.5
7.0
6.5
6.0
5.5
5.0
4.5
4.0
3.5
3.0
2.5
2.0
1.5
1.0
0.5
0.0
f1 (ppm)
2.01
0.96
2.00
2.02
2.03
3.00
8.7406
8.7285
8.2418
7.5915
7.5792
7.4769
7.4593
7.3214
7.3037
2.4694
N
CH3
N-O-C-N
O
H
Cl
19
1H NMR (500 MHz, CDCl3)

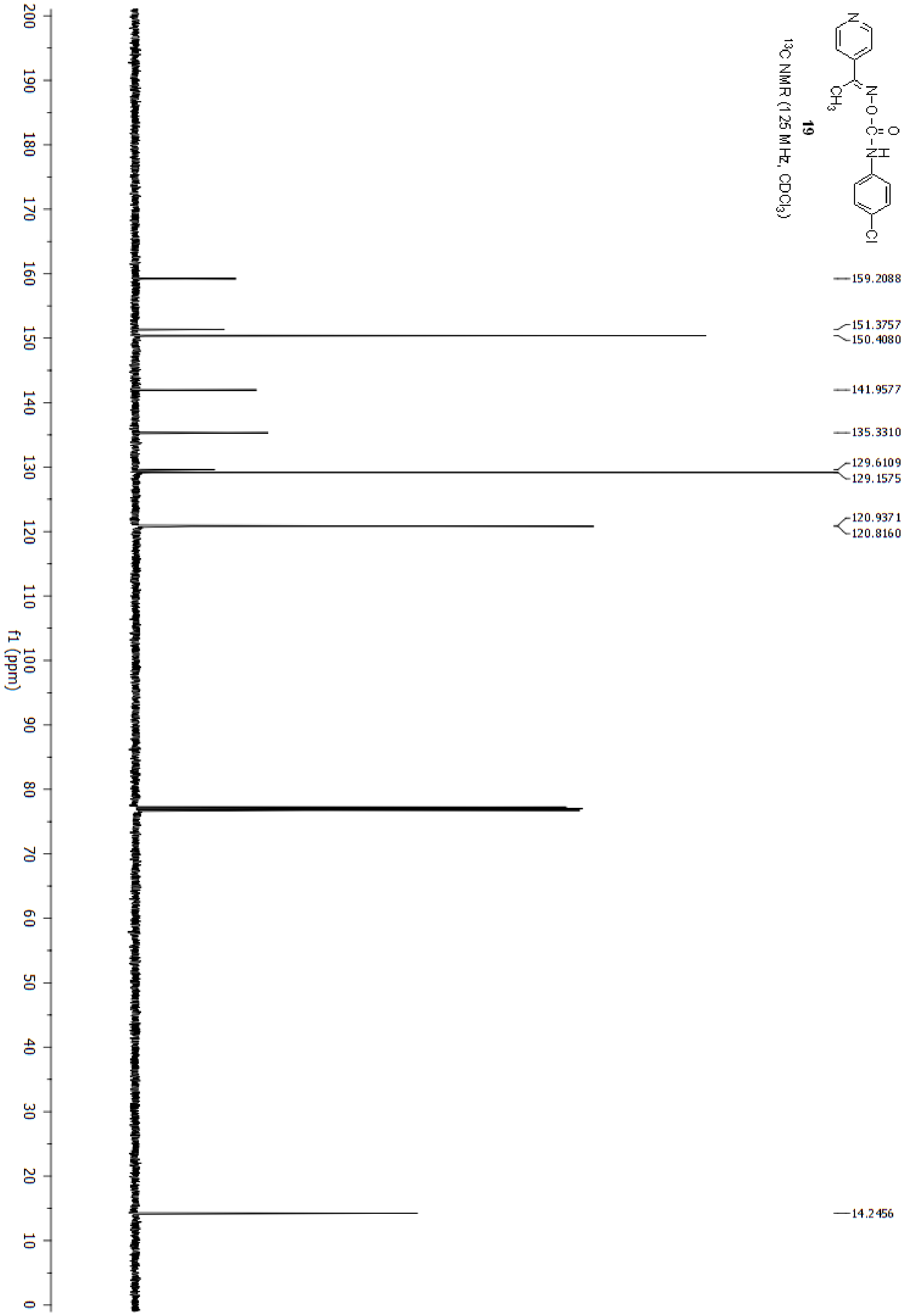
19
13C NMR (125 MHz, CDCl3)
159.2088
151.3757
150.4080
141.9577
135.3310
129.6109
129.1575
120.9371
120.8160
14.2456
f1 (ppm)

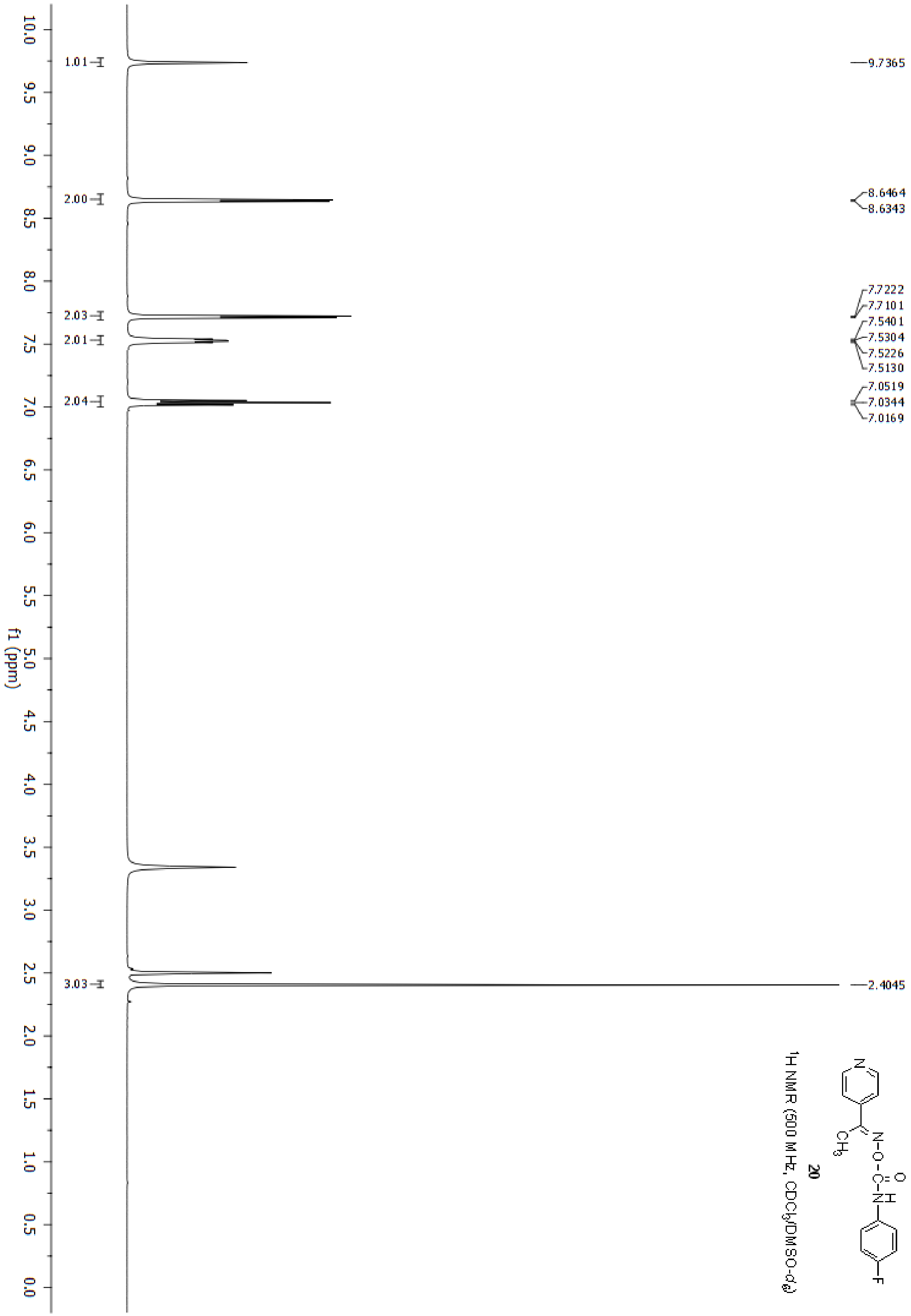
9.7365
8.6464
8.6343
7.7222
7.7101
7.5401
7.5304
7.5226
7.5130
7.0519
7.0344
7.0169
2.4045
1.01
2.00
2.03
2.01
2.04
3.03
f1 (ppm)
20
1H NMR (500 MHz, CDCl3/DMSO-d6)

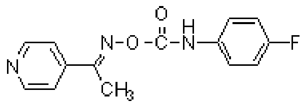

20

^{13}C NMR (125 MHz, $CDCl_3$/DMSO-d_6)

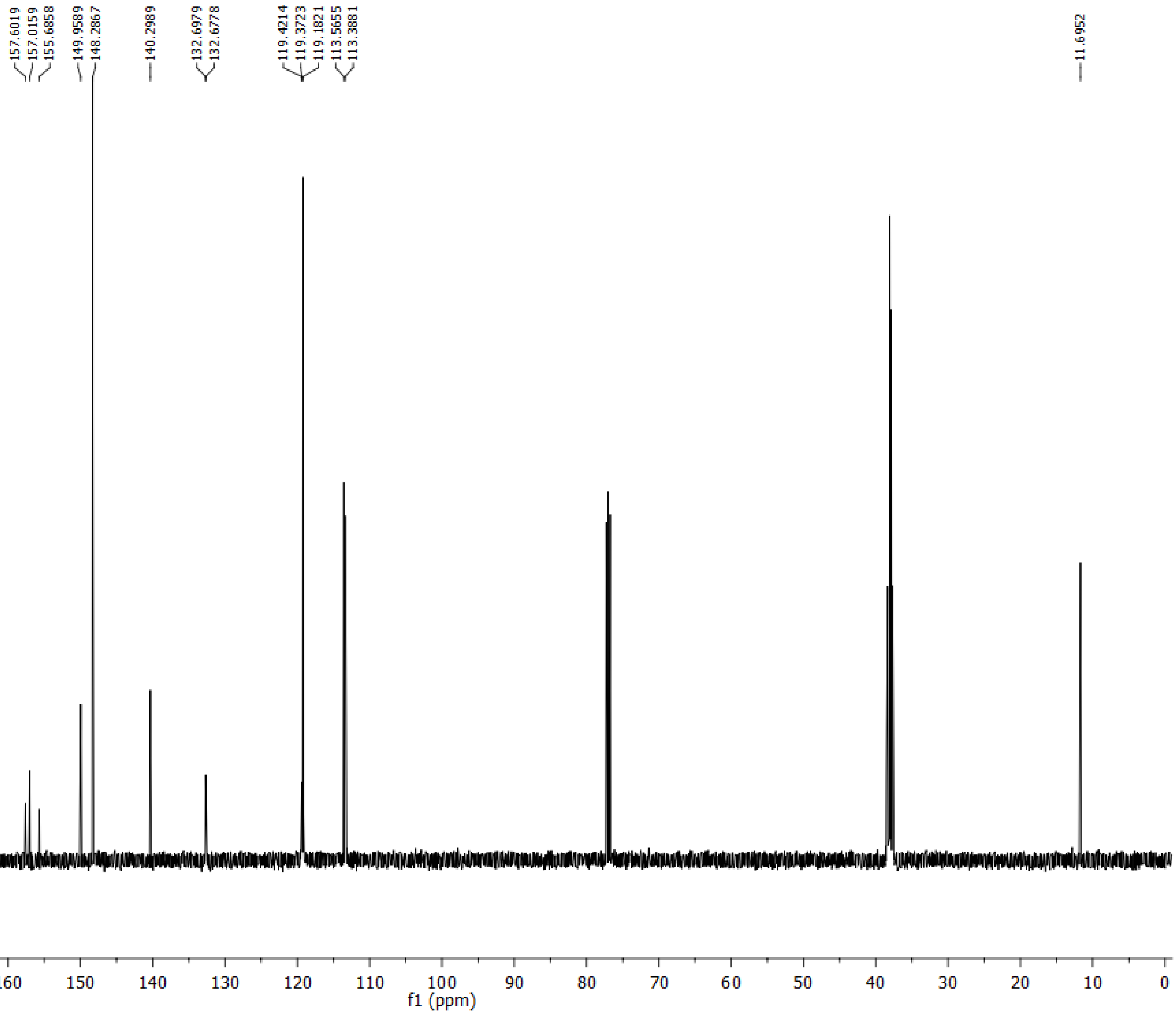

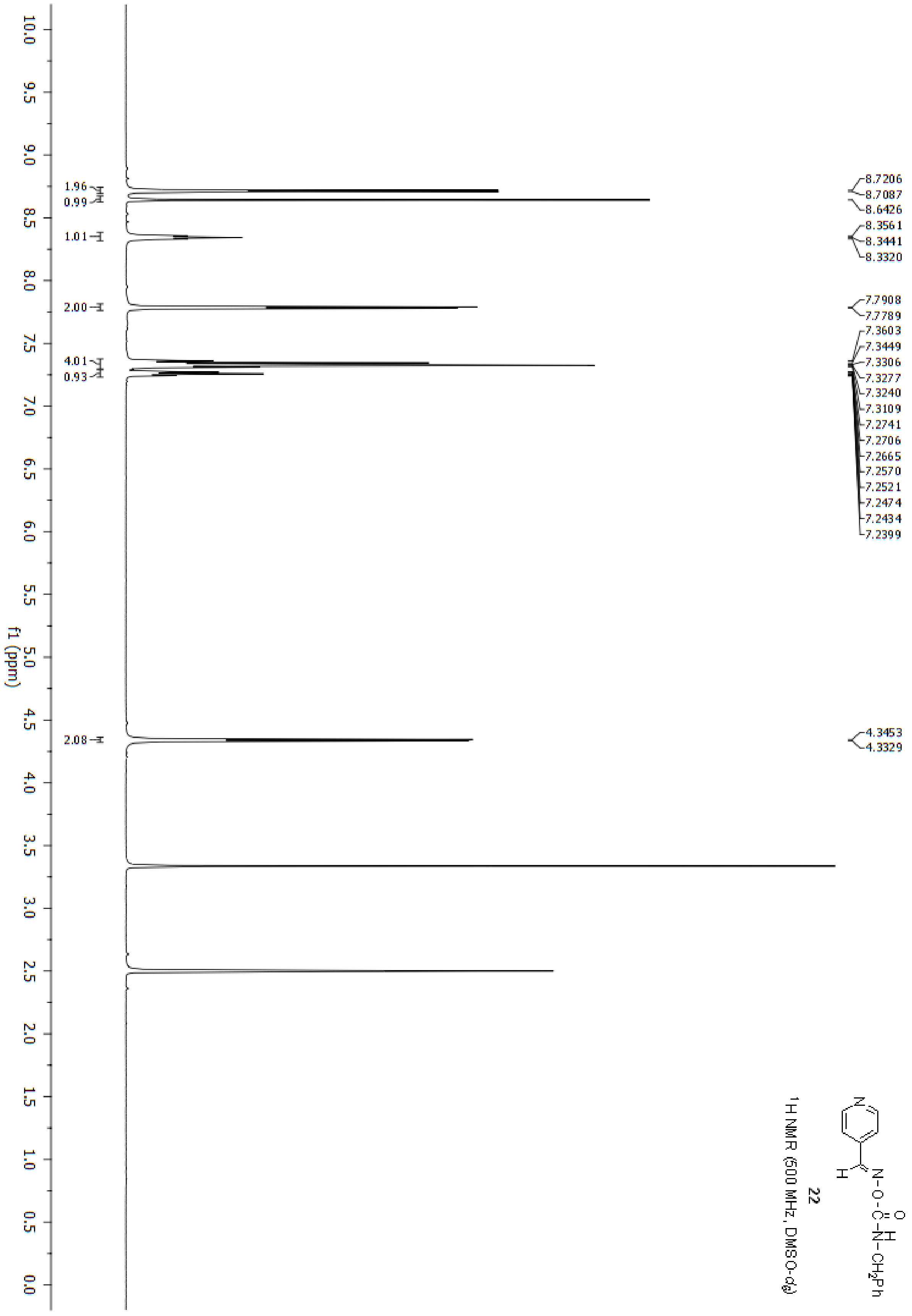
8.7206
8.7087
8.6426
8.3561
8.3441
8.3320
7.7908
7.7789
7.3603
7.3449
7.3306
7.3277
7.3240
7.3109
7.2741
7.2706
7.2665
7.2570
7.2521
7.2474
7.2434
7.2399
4.3453
4.3329
1.96
0.99
1.01
2.00
4.01
0.93
2.08
f1 (ppm)
22
1H NMR (500 MHz, DMSO-d6)

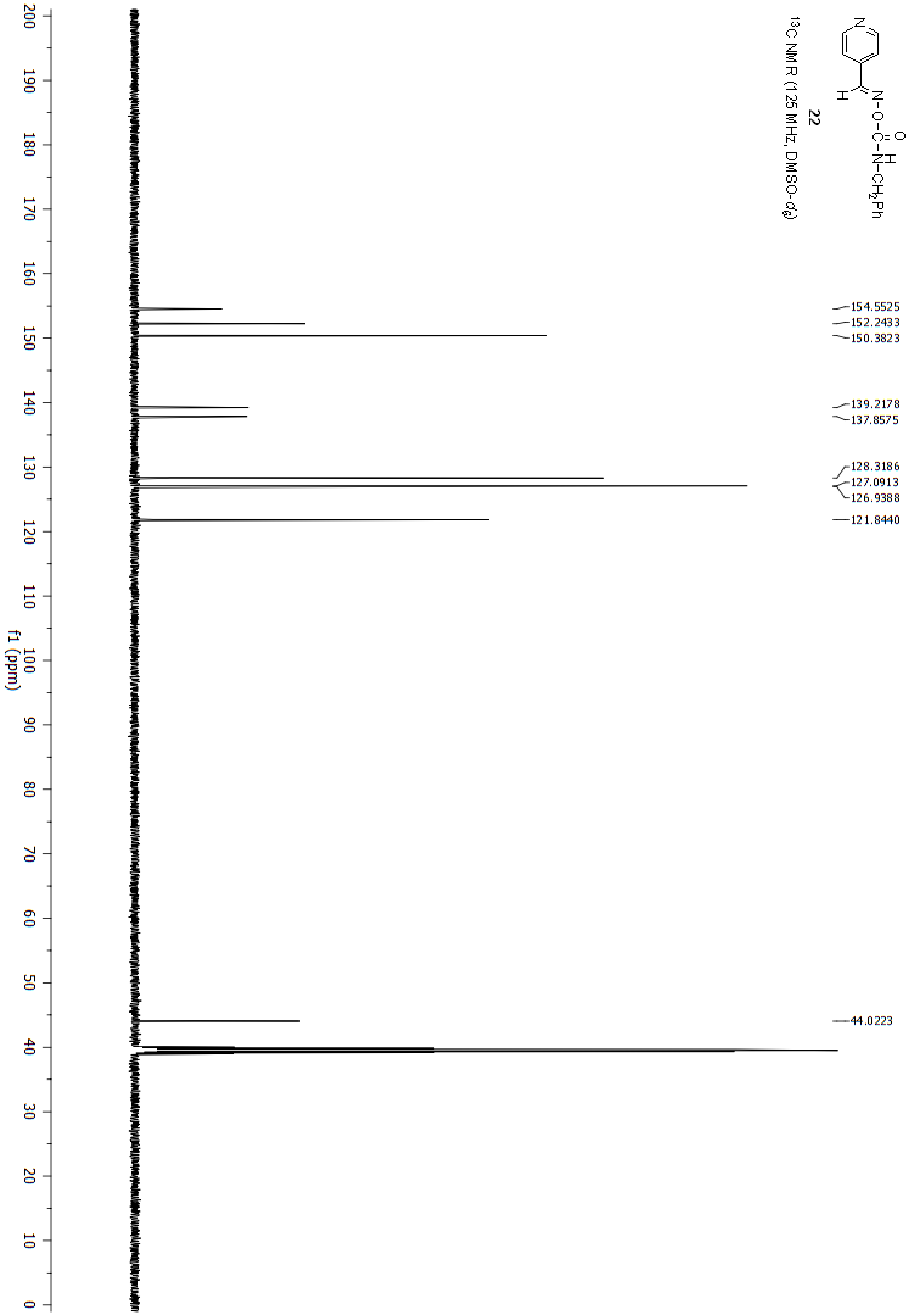
22
13C NMR (125 MHz, DMSO-d6)
154.5525
152.2433
150.3823
139.2178
137.8575
128.3186
127.0913
126.9388
121.8440
44.0223
f1 (ppm)
200
190
180
170
160
150
140
130
120
110
100
90
80
70
60
50
40
30
20
10
0

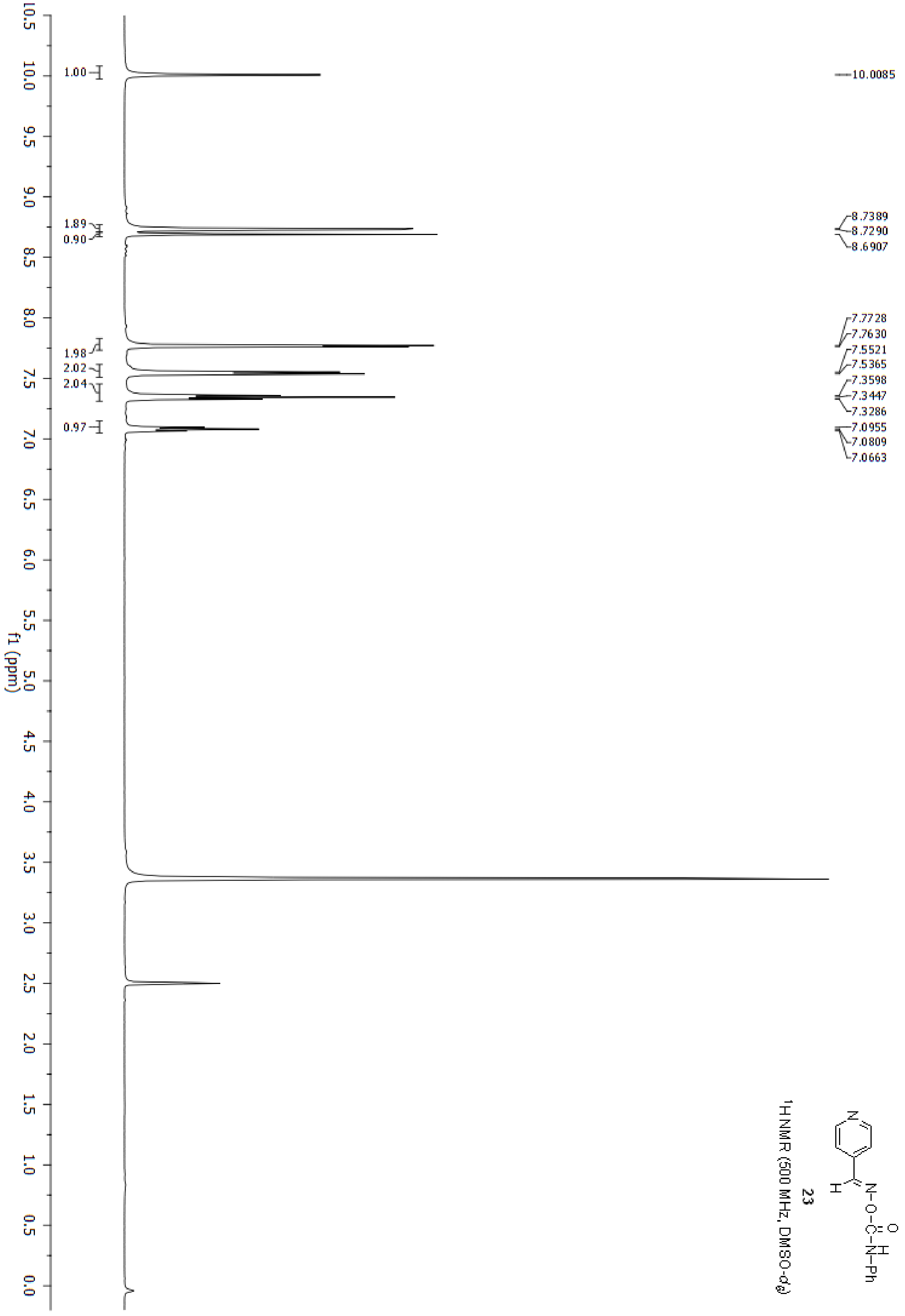

10.0085
8.7389
8.7290
8.6907
7.7728
7.7630
7.5521
7.5365
7.3598
7.3447
7.3286
7.0955
7.0809
7.0663
1.00
1.89
0.90
1.98
2.02
2.04
0.97
f1 (ppm)
23
^{1}H NMR (500 MHz, DMSO-d_6)

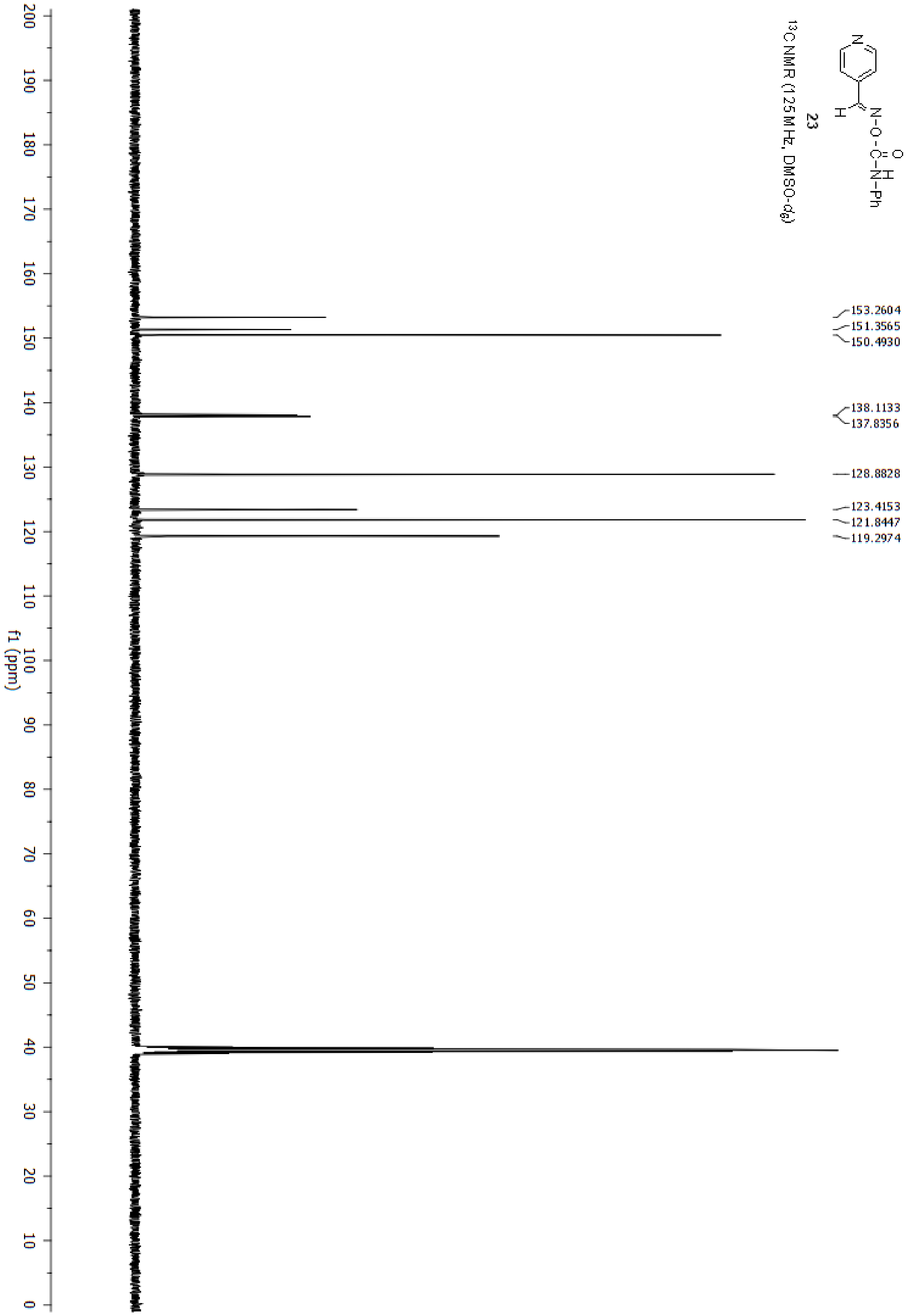

23

^{13}C NMR (125 MHz, DMSO-d_6)

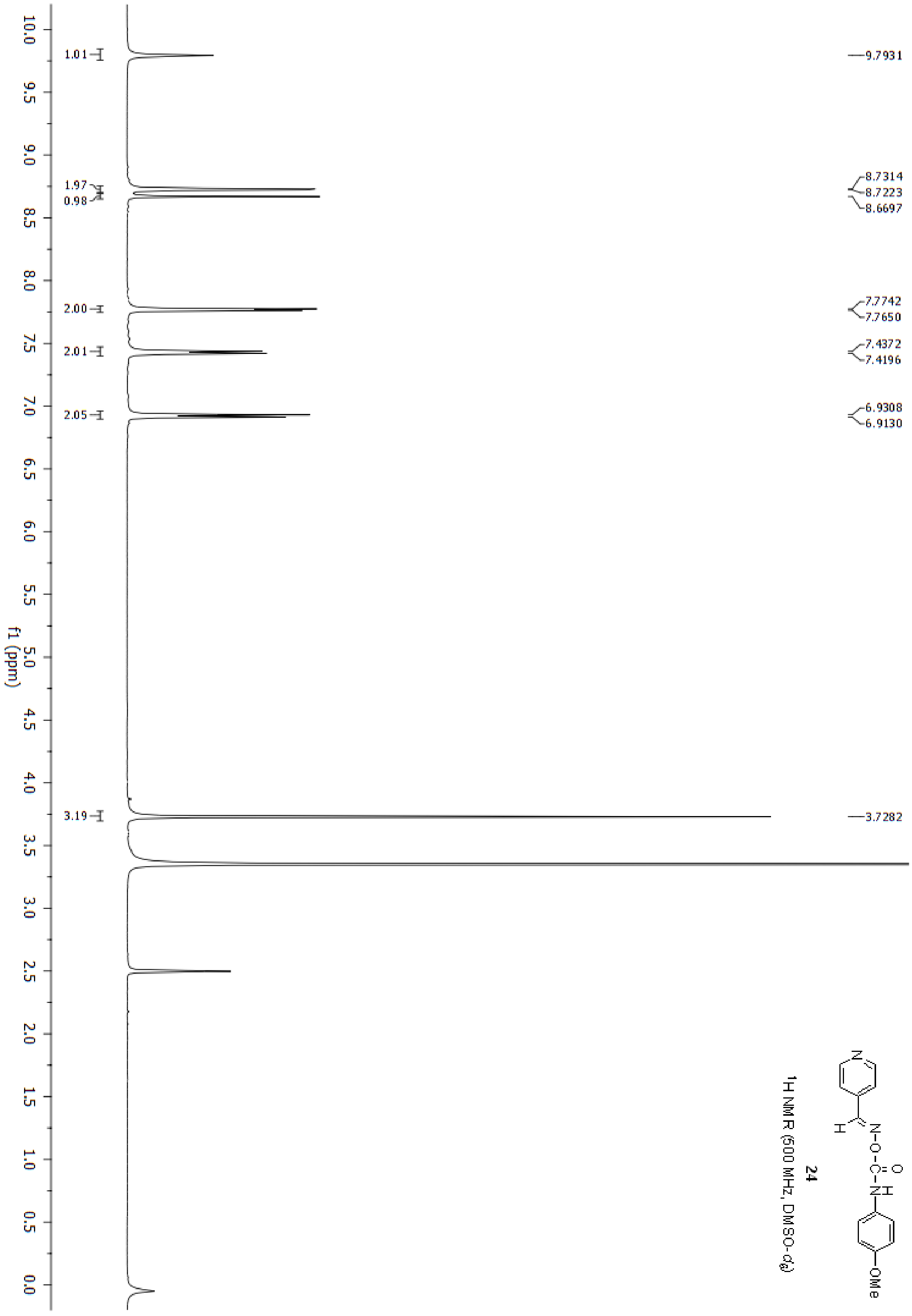

24

^{1}H NMR (600 MHz, DMSO-d_6)

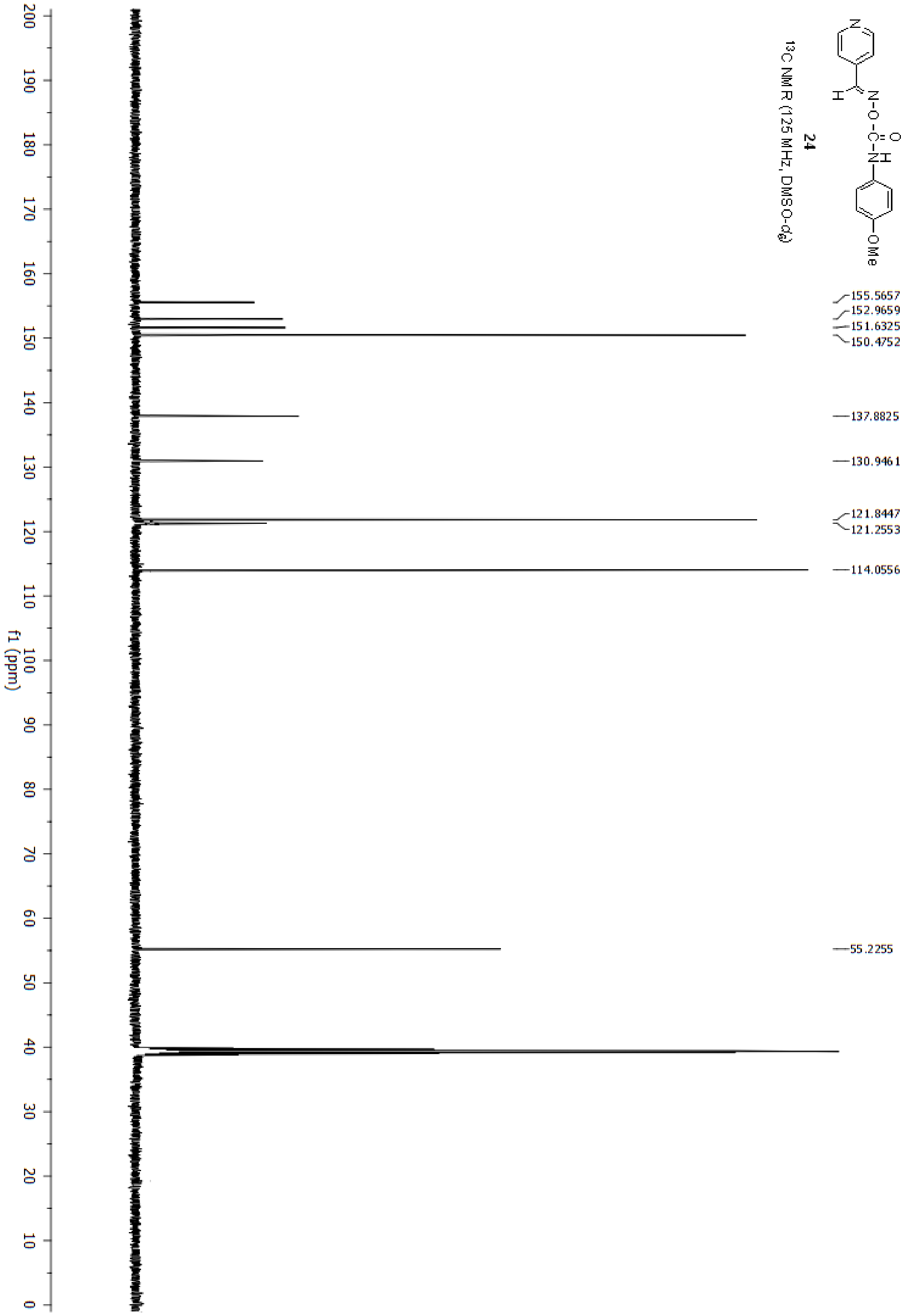

24
13C NMR (125 MHz, DMSO-d6)
OMe
155.5657
152.9659
151.6325
150.4752
137.8825
130.9461
121.8447
121.2553
114.0556
55.2255
f1 (ppm)
200
190
180
170
160
150
140
130
120
110
100
90
80
70
60
50
40
30
20
10
0

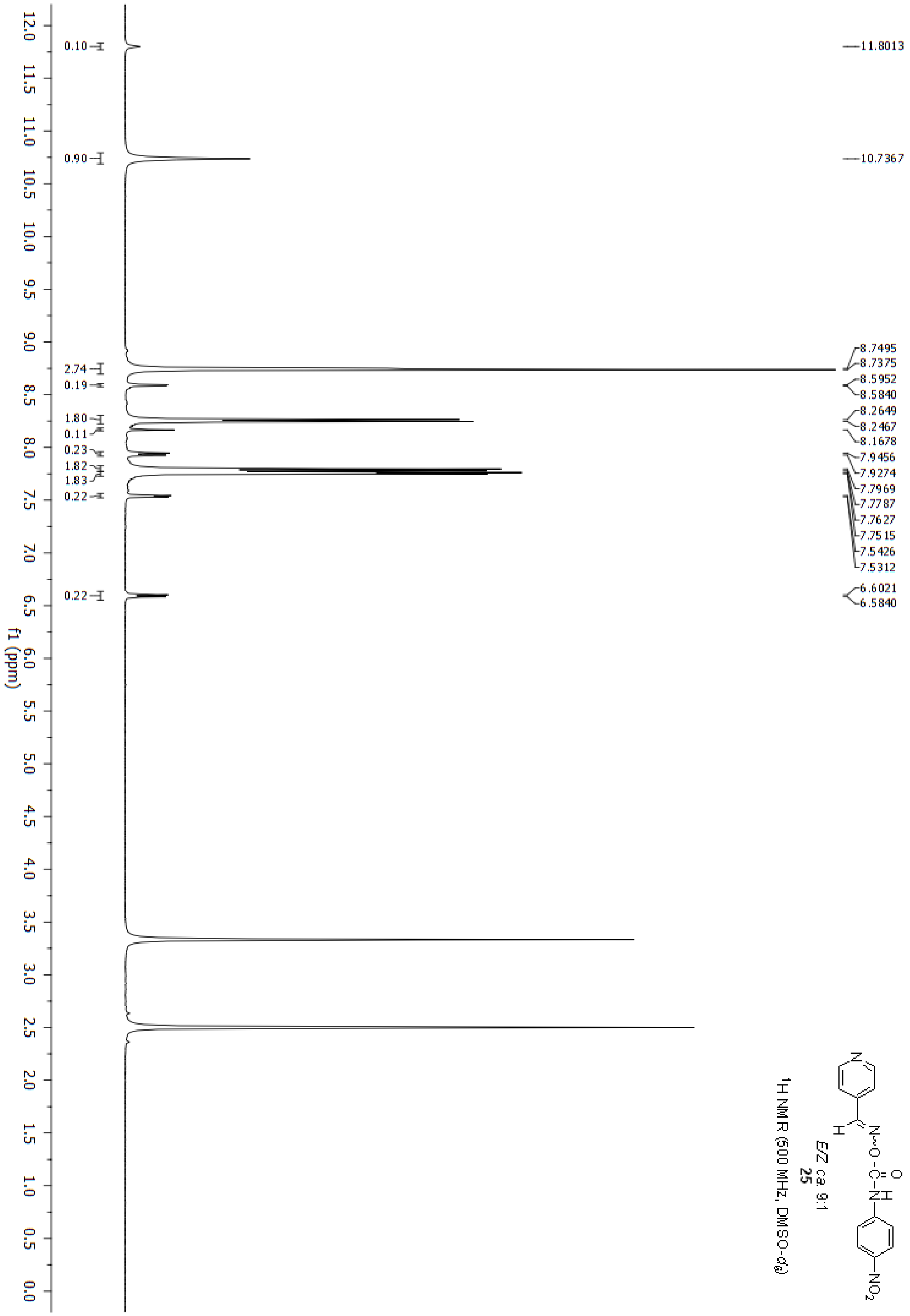

f1 (ppm)
0.10
0.90
2.74
0.19
1.80
0.11
0.23
1.82
1.83
0.22
0.22
11.8013
10.7367
8.7495
8.7375
8.5952
8.5840
8.2649
8.2467
8.1678
7.9456
7.9274
7.7969
7.7787
7.7627
7.7515
7.5426
7.5312
6.6021
6.5840
E/Z ca. 9:1
25
1H NMR (600 MHz, DMSO-d6)

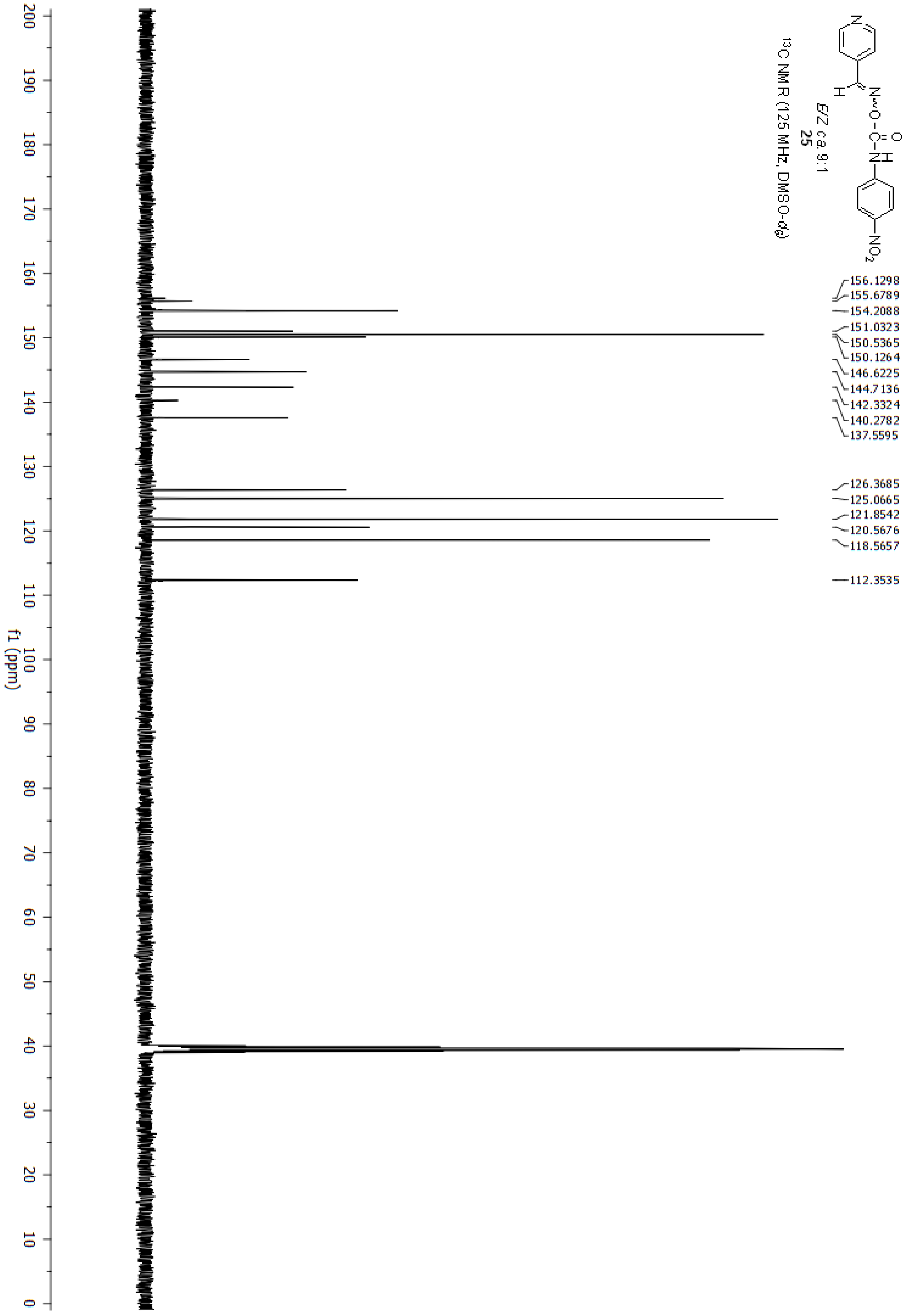
E/Z ca. 9:1
25
13C NMR (125 MHz, DMSO-d6)
156.1298
155.6789
154.2088
151.0323
150.5365
150.1264
146.6225
144.7136
142.3324
140.2782
137.5595
126.3685
125.0665
121.8542
120.5676
118.5657
112.3535
f1 (ppm)

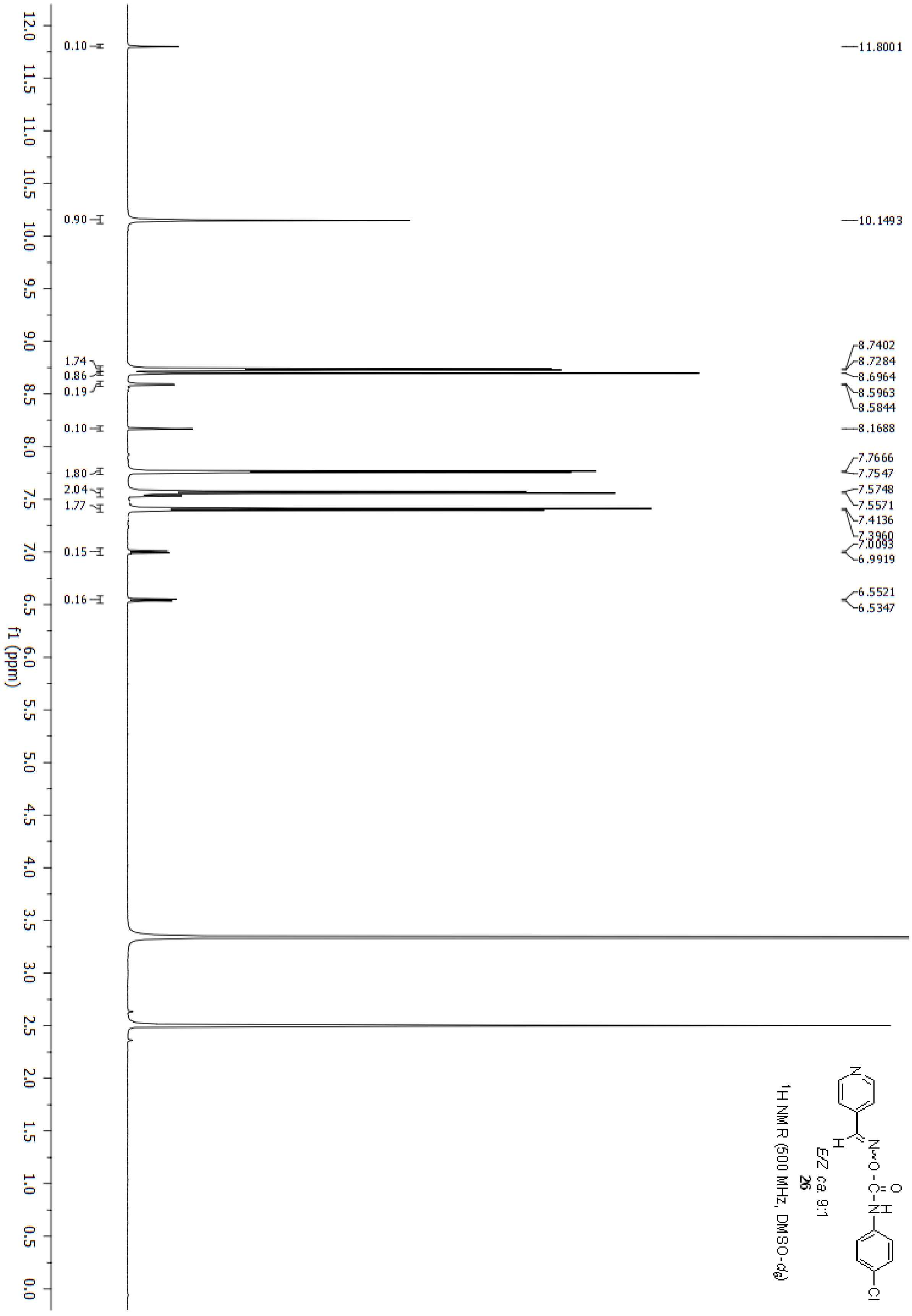

11.8001
10.1493
8.7402
8.7284
8.6964
8.5963
8.5844
8.1688
7.7666
7.7547
7.5748
7.5571
7.4136
7.3960
7.0093
6.9919
6.5521
6.5347
0.10
0.90
1.74
0.86
0.19
0.10
1.80
2.04
1.77
0.15
0.16
f1 (ppm)
E/Z ca. 9:1
26
1H NMR (500 MHz, DMSO-d6)

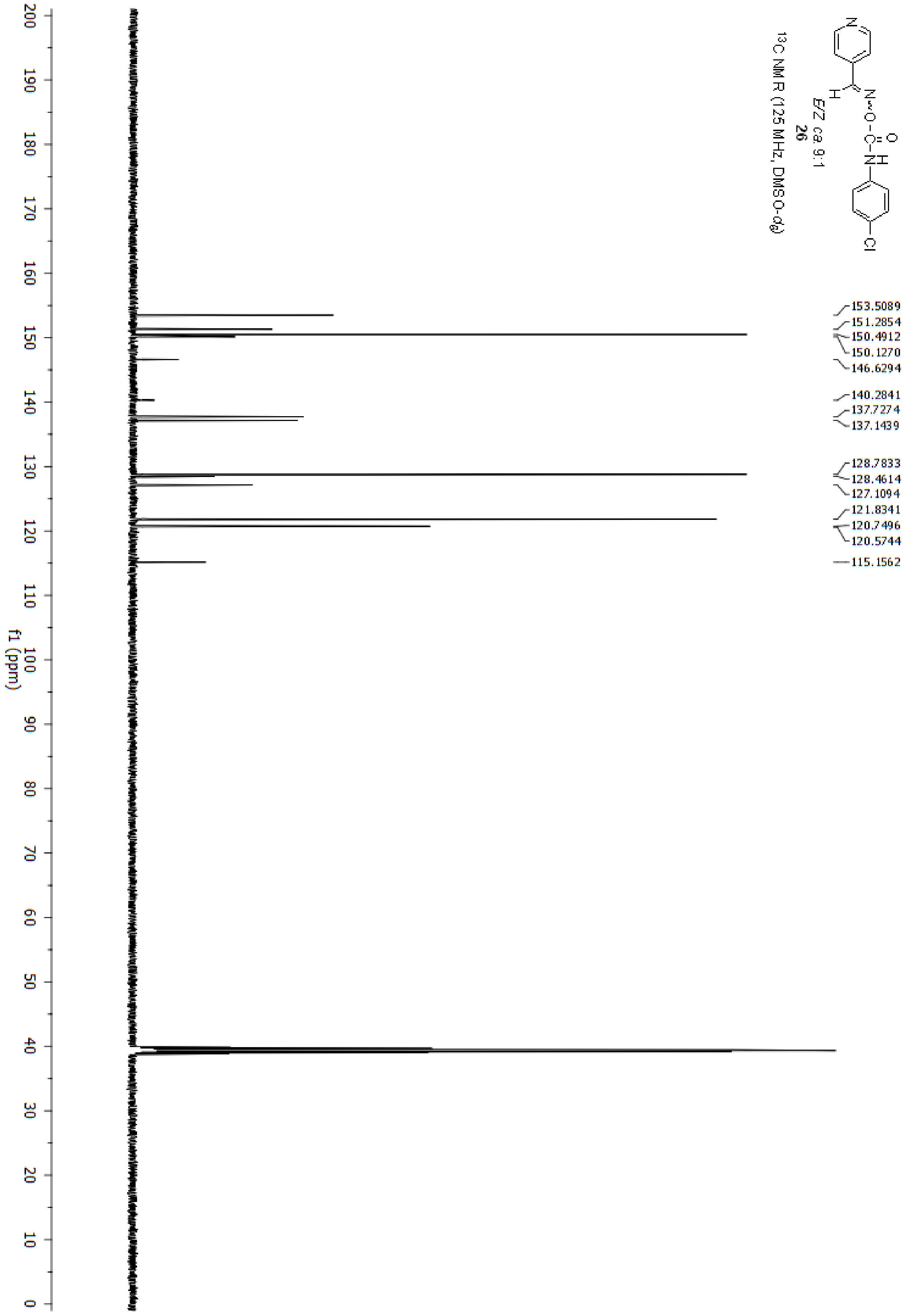
E/Z ca. 9:1
26
13C NMR (125 MHz, DMSO-d6)
153.5089
151.2854
150.4912
150.1270
146.6294
140.2841
137.7274
137.1439
128.7833
128.4614
127.1094
121.8341
120.7496
120.5744
115.1562
f1 (ppm)

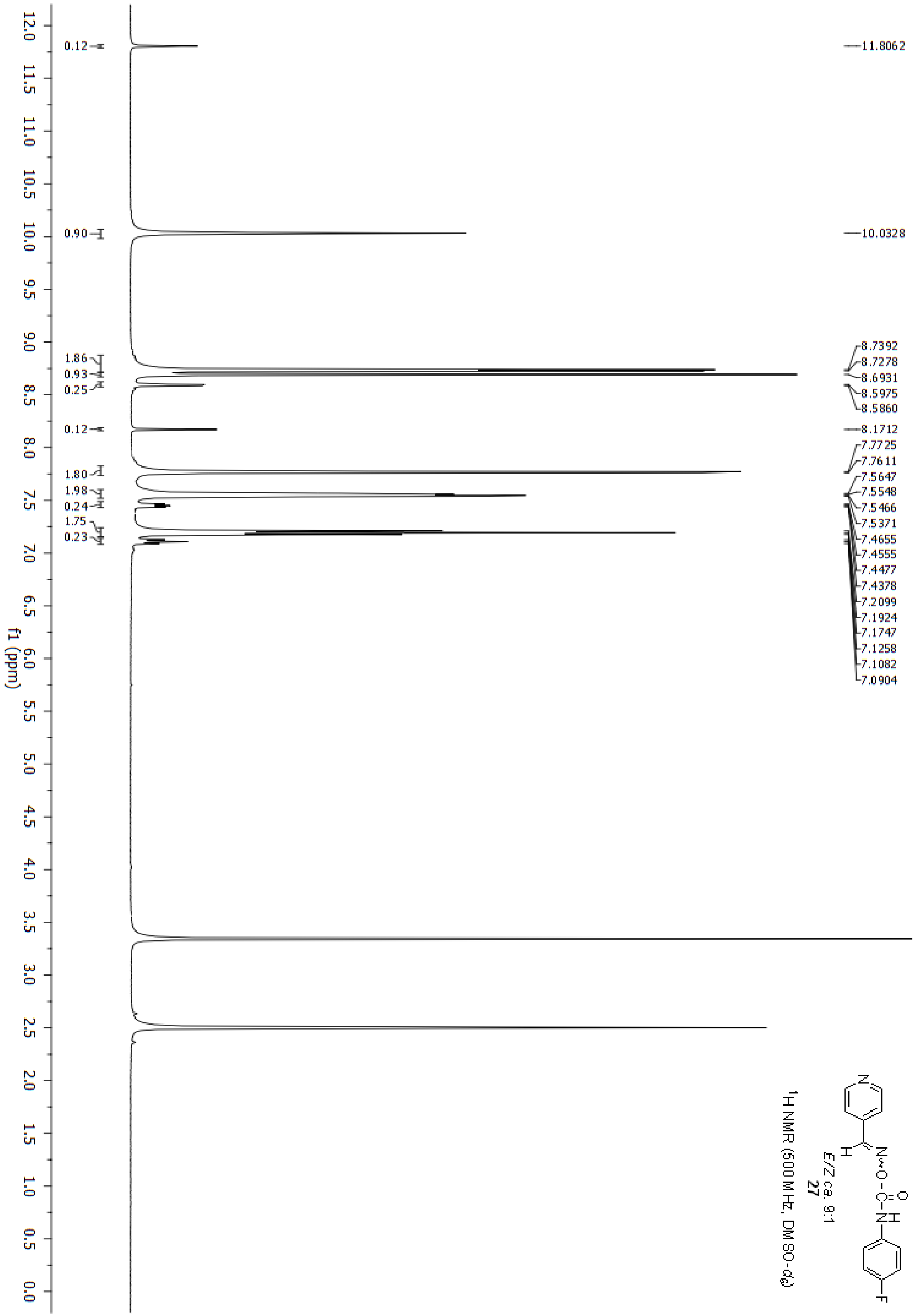

11.8062
10.0328
8.7392
8.7278
8.6931
8.5975
8.5860
8.1712
7.7725
7.7611
7.5647
7.5548
7.5466
7.5371
7.4655
7.4555
7.4477
7.4378
7.2099
7.1924
7.1747
7.1258
7.1082
7.0904
0.12
0.90
1.86
0.93
0.25
0.12
1.80
1.98
0.24
1.75
0.23
f1 (ppm)
E/Z ca. 9:1
27
1H NMR (500 MHz, DMSO-d6)

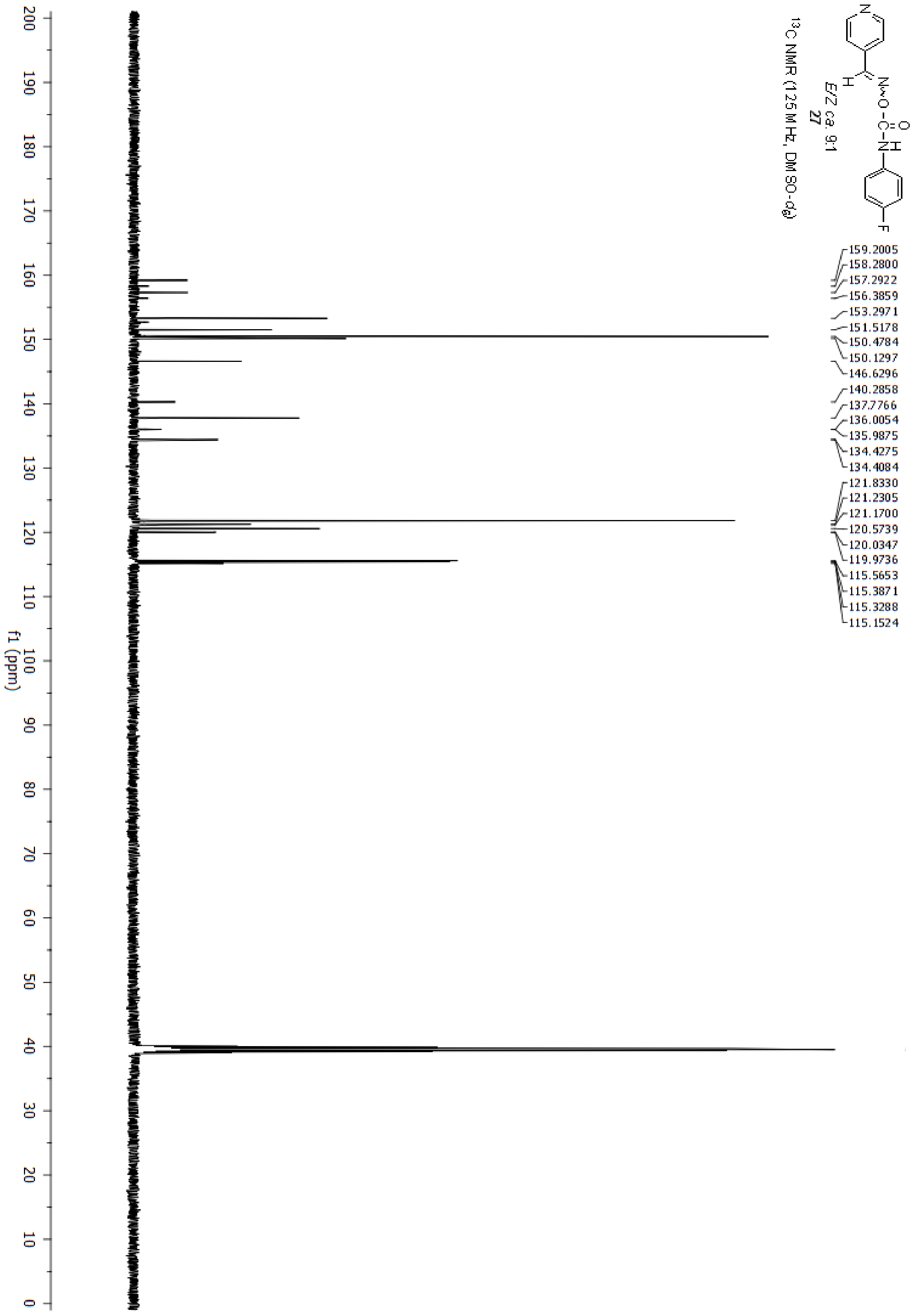

E/Z ca. 9:1
27
^{13}C NMR (125 MHz, DMSO-d_6)
159.2005
158.2800
157.2922
156.3859
153.2971
151.5178
150.4784
150.1297
146.6296
140.2858
137.7766
136.0054
135.9875
134.4275
134.4084
121.8330
121.2305
121.1700
120.5739
120.0347
119.9736
115.5653
115.3871
115.3288
115.1524
f1 (ppm)

3. *Optimized molecular geometries for NMR prediction*

1. Optimized molecular geometries of *E*-25-DMSO (A) and *Z*-25-DMSO (B) complexes at B3LYP/631G(d) level

A. 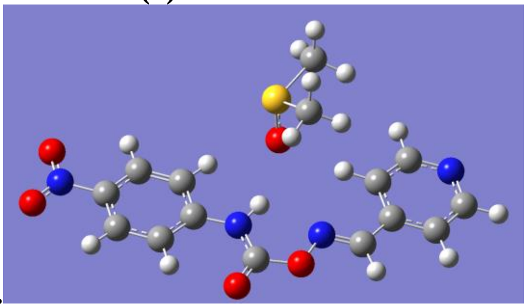 **B.** 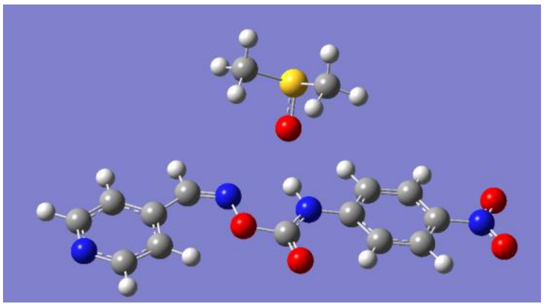

2. Optimized molecular geometries of *E*-26-DMSO (A) and *Z*-26-DMSO (B) complexes at B3LYP/631G(d) level

A. 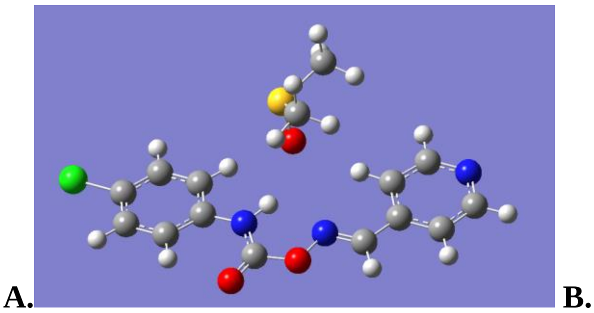 **B.** 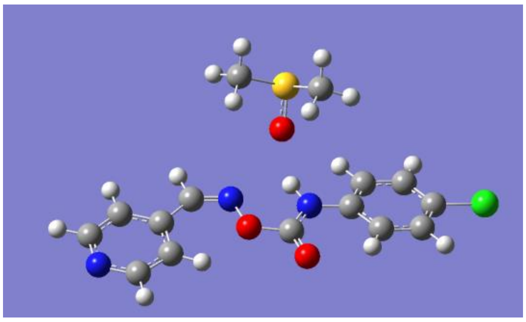

3. Optimized molecular geometries of *E*-27-DMSO (A) and *Z*-27-DMSO (B) complexes at B3LYP/631G(d) level

A. 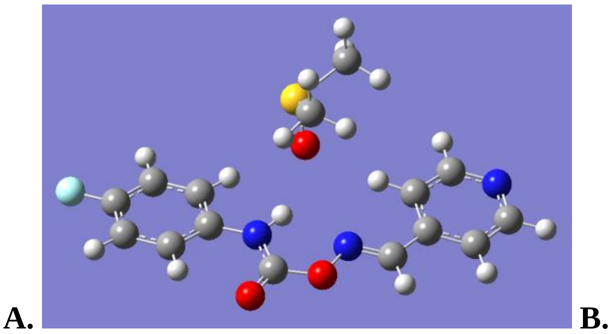 **B.** 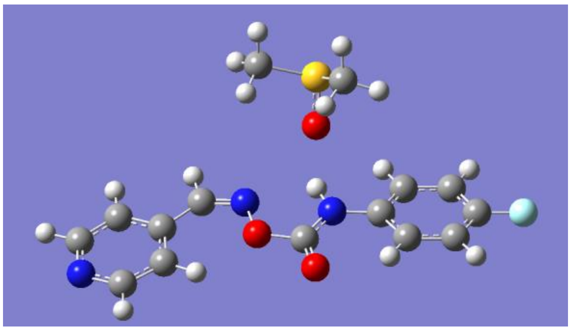

4. ***DNA binding studies***

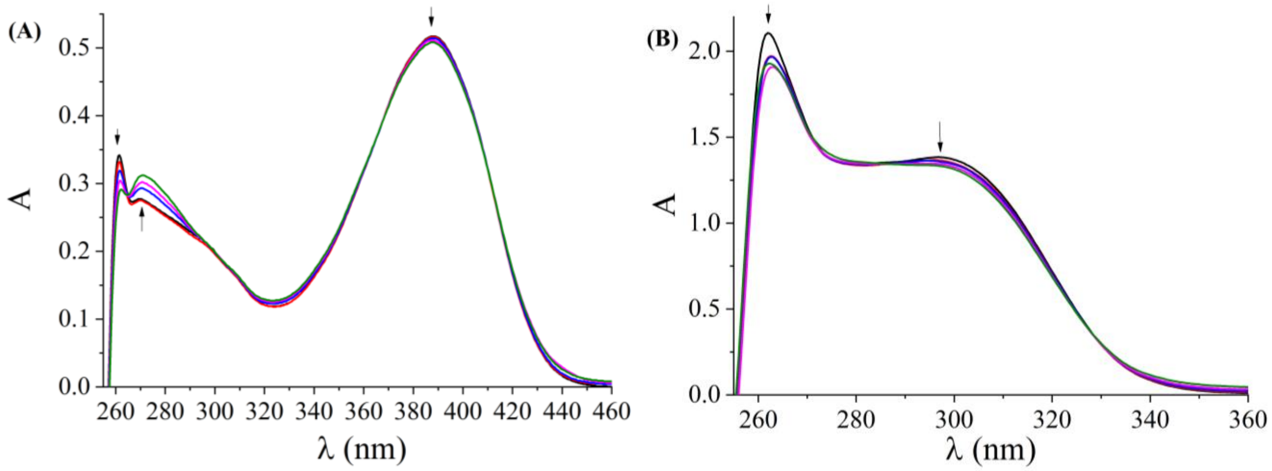

Figure S-4.1. UV–vis spectra of DMSO solution of compound (A) **11** (1×10^{-4} M) and (B) **12** (1×10^{-4} M) in the presence of increasing amounts of CT DNA (r' = [DNA]/[compound] = 0–0.8). The arrows show the changes upon increasing amounts of CT DNA.

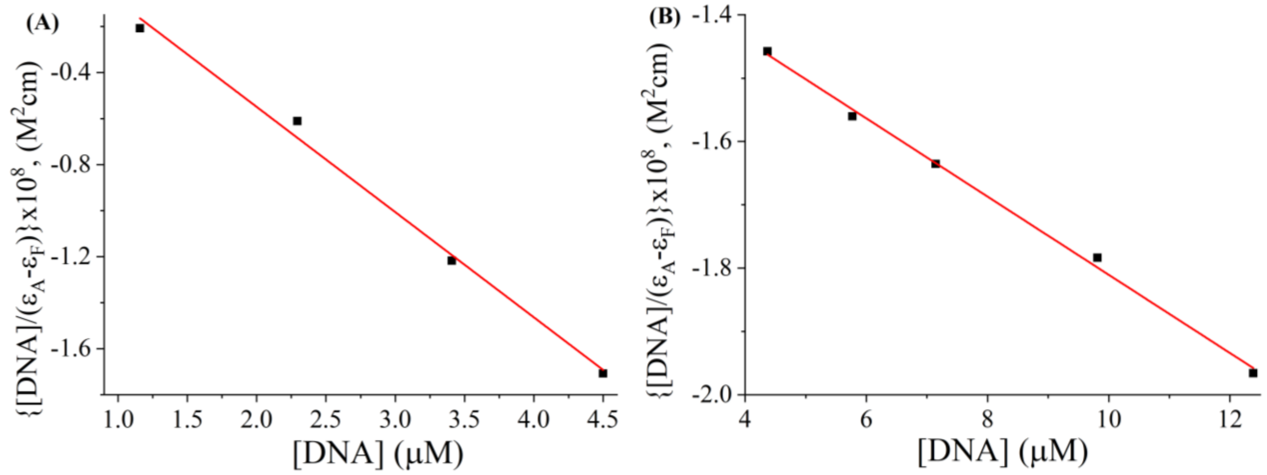

Figure S-4.2. Plot of [DNA]/(ε_A-ε_f) versus [DNA] for compound (A) **11** and (B) **12**.

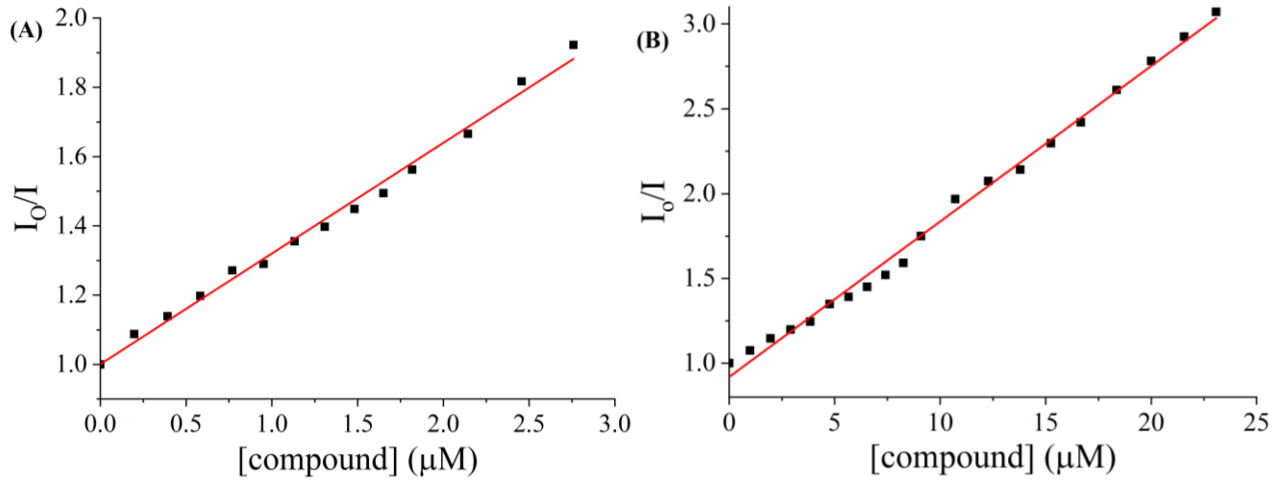

Figure S-4.3. Stern–Volmer quenching plot of EB bound to CT DNA for compound (A) **11** and (B) **12**.

5. *UV absorption spectra of amidoxime, ethanone oxime and aldoxime carbamates*

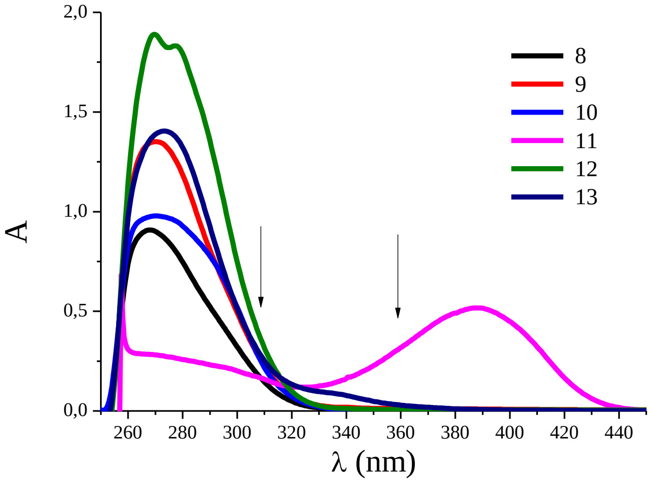

Concentration
**8:** 10^{-4} M
**9:** 10^{-4} M
**10:** 10^{-4} M
**11:** $2x10^{-5}$ M
**12:** 10^{-4} M
**13:** 10^{-4} M

Figure S-5.1. UV–vis spectra of amidoxime carbamates **8–13**.

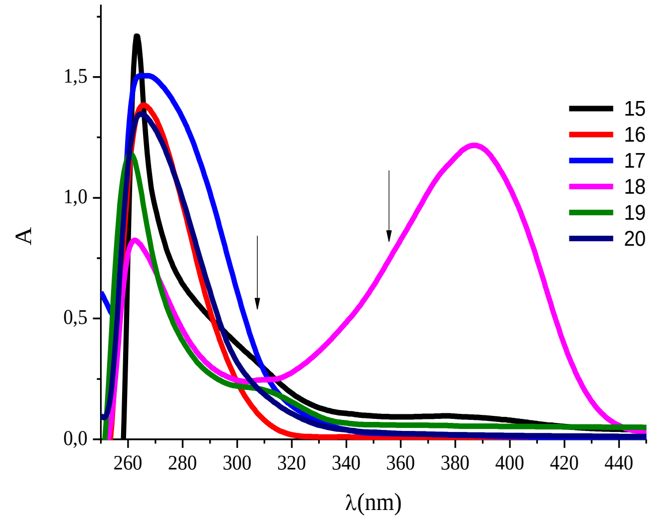

Concentration
**15:** 10^{-4} M
**16:** 10^{-4} M
**17:** 10^{-4} M
**18:** 10^{-4} M
**19:** 10^{-4} M
**20:** 10^{-4} M

Figure S-5.2. UV–vis spectra of ethanone oxime carbamates **15–20**.

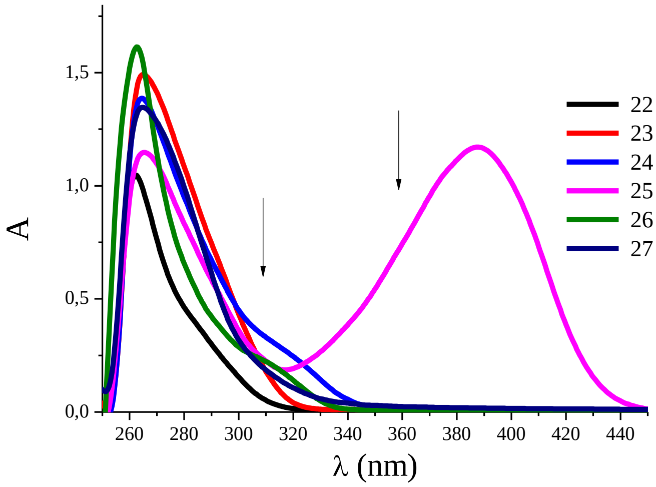

Concentration
**22:** 10^{-4} M
**23:** 10^{-4} M
**24:** 10^{-4} M
**25:** $5x10^{-5}$ M
**26:** 10^{-4} M
**27:** 10^{-4} M

Figure S-5.3. UV–vis spectra of aldoxime carbamates **22–27**.

6. *Gel electrophoresis pictures of amidoxime, ethanone oxime and aldoxime carbamates*

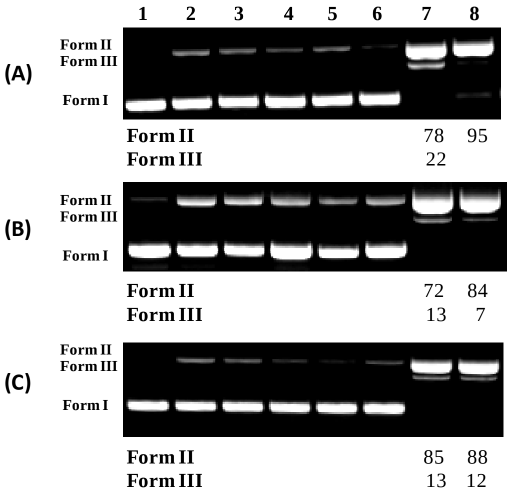

Figure S-6.1. DNA photo-cleavage at concentration of 500 μM. Gel electrophoreses pictures, Top: (**A**): Lane 1: DNA without UV irradiation; Lane 2: DNA with UV irradiation; Lanes 3–8: DNA + carbamoyl amidoximes (**8** or **9**, or **10**, or **11**, or **12**, or **13**, respectively) + UV irradiation; (**B**): Lane 1: DNA without UV irradiation; Lane 2: DNA with UV irradiation; Lanes 3–8: DNA + carbamoyl ethanone oximes (**15** or **16**, or **17**, or **18**, or **19**, or **20**, respectively) + UV irradiation; (**C**): Lane 1: DNA without UV irradiation; Lane 2: DNA with UV irradiation; Lanes 3–8: DNA + carbamoyl aldoximes (**22** or **23**, or **24**, or **25**, or **26**, or **27**, respectively) + UV irradiation; Bottom: Calculation of the % conversion to ss and ds damage.

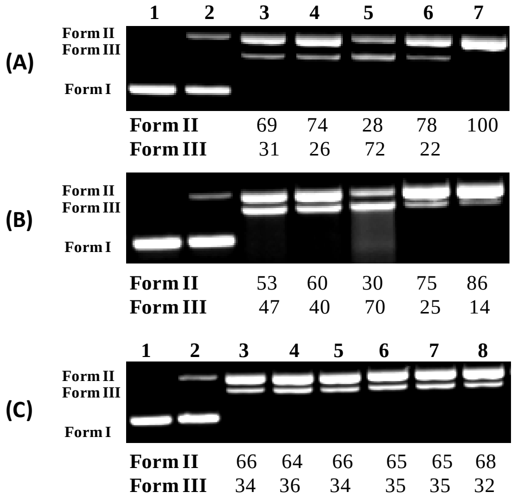

Figure S-6.2. DNA photo-cleavage at concentration of 500 μM. Gel electrophoreses pictures, Top: (**A**): Mechanistic studies involved by derivative **12**. Lane 1: DNA without UV irradiation; Lane 2: DNA with UV irradiation; Lane 3: DNA + **12**; Lane 4: DNA + **12** + argon; Lane 5: DNA + **12** + DMSO (20%); lane 6: DNA + **12** + NaN_3 (20 mM); lane 7: DNA + **12** + D_2O; (**B**): Mechanistic studies involved by derivative **26**. Lane 1: DNA without UV irradiation; Lane 2: DNA with UV irradiation; Lane 3: DNA + **26**; Lane 4: DNA + **26** + argon; Lane 5: DNA + **26** + DMSO (20%); lane 6: DNA + **26** + NaN_3 (20 mM); lane 7: DNA + **26** + D_2O; (**C**): Effect of pH on the cleavage of compound **12**. Lane 1: DNA without UV irradiation; Lane 2: DNA with UV irradiation; Lane 3−8: DNA + **12** + UV irradiation at pH 5, 6, 7, 8, 9, 10, respectively; Bottom: Calculation of the % conversion to ss and ds damage.

7. *UV absorption spectra of amidoxime carbamates 11 and 12 under irradiation*

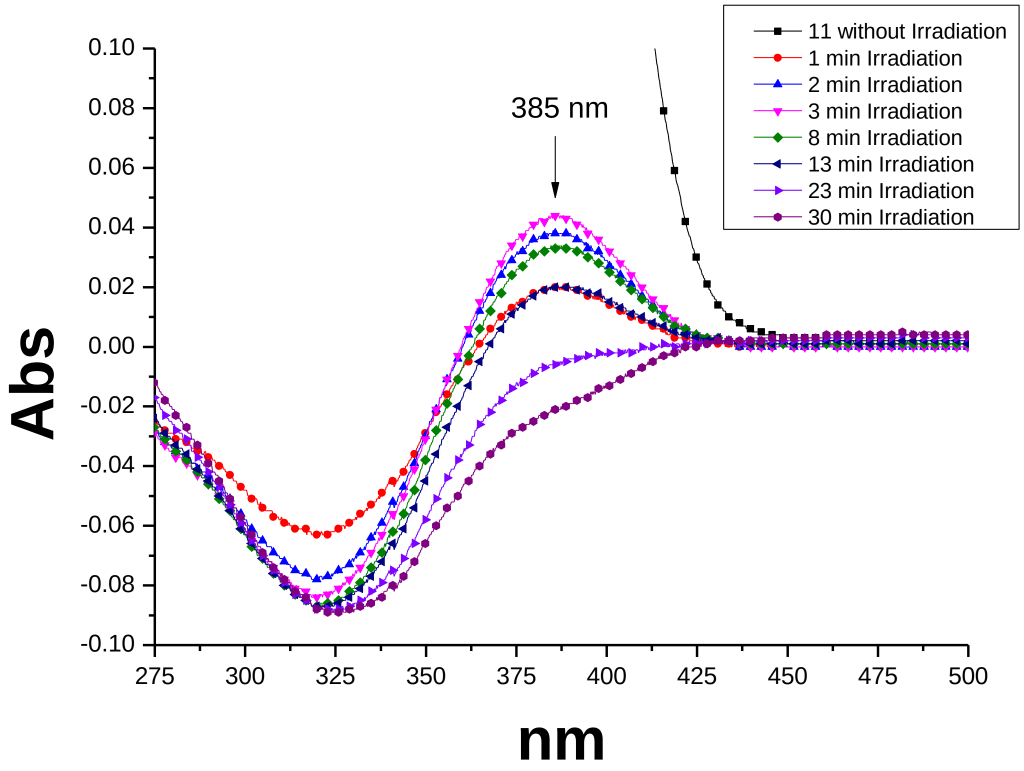

Figure S-7.1. UV absorption spectrum of amidoxime carbamate **11** under irradiation (312 nm).

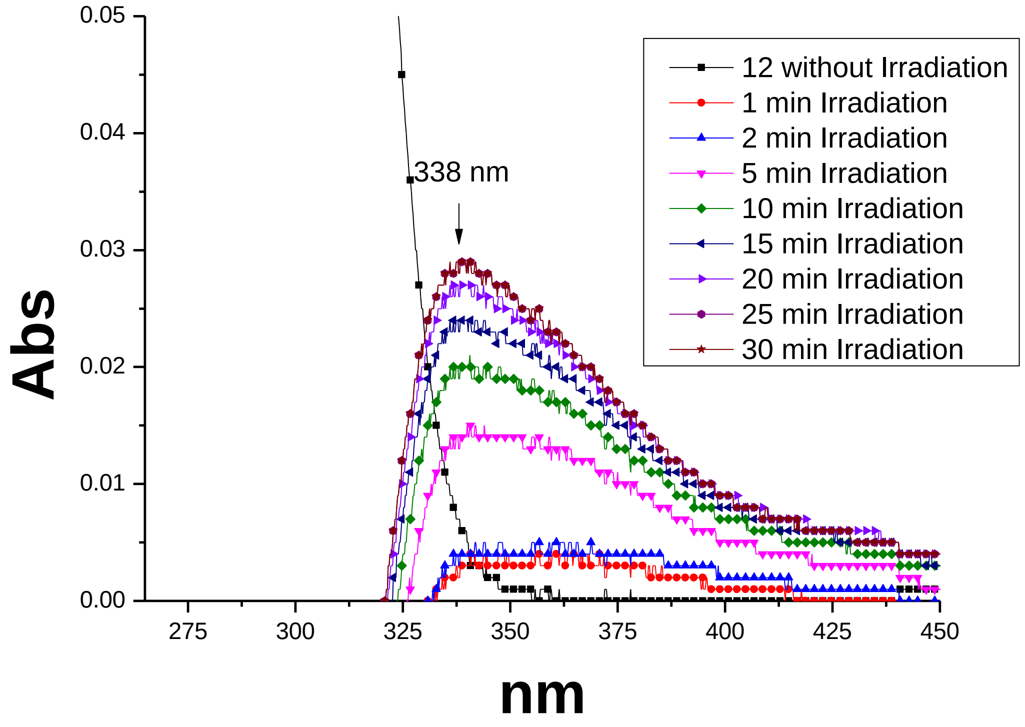

Figure S-7.2. UV absorption spectrum of amidoxime carbamate **12** under irradiation (312 nm).

8. *A computational study and photochemical aspects of compounds 11 and 12*

8.1. Ground state energies

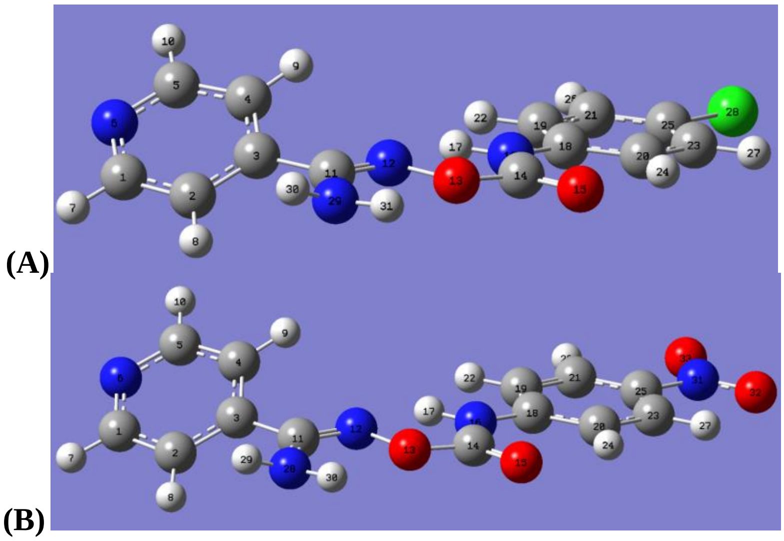

(A)

(B)

Figure S-8.1. Ground state (S_0) structures of **12** (A) and **11** (B).

Table S-8.1. Molecular geometries of compounds 12 and 11. Selected B3PW91/6-31G(d) geometrical parameters for compounds **12** and **11** in the ground (S_0) and lowest triplet excited state (T_1)[a] in aqueous solution.

comp.	state	r_{C3C11}	r_{C11N12}	r_{N12O13}	r_{O13C14}	r_{C14N16}	φ_1	φ_2
**12**	S_0	1.483	1.299	1.423	1.370	1.357	162.61	164.02
	T_1	1.421	1.409	1.386	1.408	1.347	92.31	91.66
**11**	S_0	1.484	1.300	1.425	1.364	1.365	162.68	163.67
	T_1	1.484	1.300	1.424	1.367	1.360	162.41	163.84

[a]Bond lengths (r) in Angstroms (Å) and angles,φ (dihedral) in degrees. The values are given according to the suggestions of Hoffmann, Schleyer and Schaefer III [3], $\varphi_1 = \varphi$(C4C11C18C20), $\varphi_2 = \varphi$(C4C11N12O13).

8.2. Franck–Condon excitation energies

Table S-8.2. Franck–Condon excitation energies. Franck–Condon (vertical) excitation energies (ΔE_{ex}/kcal·mol^{-1}) and their corresponding wave-lengths (λ/nm) for compounds **12** and **11** [PBE0/6-31G(d)//B3PW91/6-31G(d)]

comp./ state	T_1		T_2		T_3		S_1		S_2		S_3	
	ΔE_{ex}	λ	ΔE_{ex}	λ	ΔE_{ex}	λ	ΔE_{ex}	λ	ΔE_{ex}	λ	ΔE_{ex}	λ
**12**	74.38	384.36	78.45	364.44	87.87	325.37	91.75	311.61	95.60	299.05	99.17	288.29
**11**	66.18	432.05	67.18	425.58	75.30	379.32	77.33	369.72	88.00	324.90	90.79	314.92

8.3. Potential energy surface for the dissociation of 11 in T_1 excited state in aqueous solution

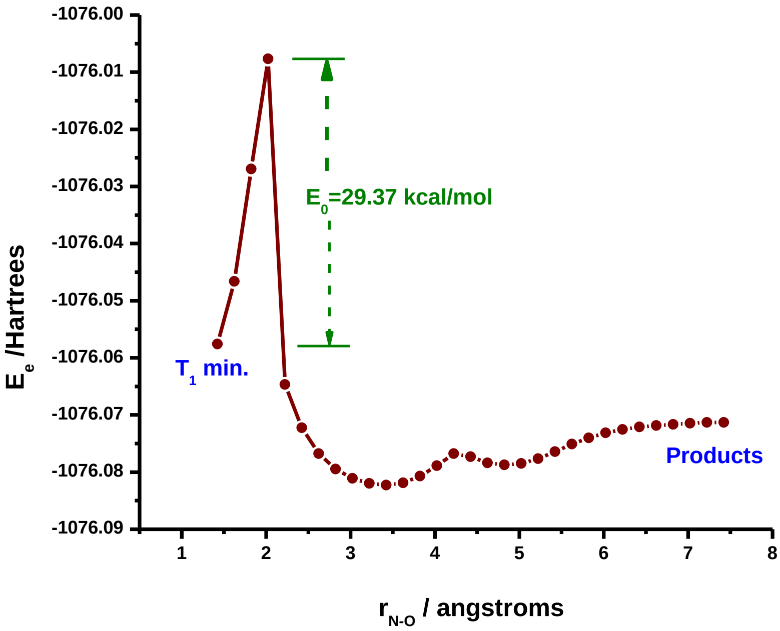

Figure S-8.3. PES for the dissociation of **11** in the first excited triplet state. The reaction coordinate is the N12–O13 bond. The products are ground state radicals.

8.4. Mathematical appendix

In this section we shall prove that the infinite series given in equation (8) of the paper,

$$\kappa(T)=\sum_{n=0}^{\infty}(-1)^n\beta\cdot\left[\frac{1-e^{\{[\beta-(n+1)\alpha]\cdot\Delta E_0^{\neq}\}}}{(n+1)\alpha-\beta}+\frac{1}{n\alpha+\beta}\right] \quad (1)$$

is convergent and we shall compute its sum. First we note that the above series can be written as a sum of three terms,

$$\kappa(T)=\sum_{n=0}^{\infty}\frac{(-1)^n\beta}{(n+1)\alpha-\beta}+\sum_{n=0}^{\infty}\frac{(-1)^n\beta}{n\alpha+\beta}-\sum_{n=0}^{\infty}\frac{(-1)^n\beta e^{[\beta-(n+1)\alpha]\Delta E_0^{\neq}}}{(n+1)\alpha-\beta} \quad (2)$$

Consider first the third series. In order to test it for convergence we'll need the Ratio or D'Alembert's test [1-2]: if $\sum u_n$ is an infinite series and if $\lim_{n\to\infty}\left|u_{n+1}/u_n\right|=l$, then if (i) $l<1$ the series is absolutely convergent and hence is convergent, (ii) $l>1$ the series is divergent, while if $l=1$ the test gives no information. For our case, α > β > 0 and $\alpha\cdot\Delta E^{\neq}_0 = 12.54 >> 1$,

$$\lim_{n\to\infty}\left|\frac{u_{n+1}}{u_n}\right|=\lim_{n\to\infty}\left|\frac{(-1)^{n+1}\beta e^{[\beta-(n+2)\alpha]\Delta E_0^{\neq}}/[(n+2)\alpha-\beta]}{(-1)^n\beta e^{[\beta-(n+1)\alpha]\Delta E_0^{\neq}}/[(n+1)\alpha-\beta]}\right|=\lim_{n\to\infty}\left|\frac{(n+1)\alpha-\beta}{(n+2)\alpha-\beta}\cdot e^{-\alpha\Delta E_0^{\neq}}\right|$$

$$=\lim_{n\to\infty}\left|\frac{\left(1+\frac{1}{n}\right)\alpha-\frac{\beta}{n}}{\left(1+\frac{2}{n}\right)\alpha-\frac{\beta}{n}}\right|\cdot e^{-\alpha\Delta E_0^{\neq}}=e^{-\alpha\Delta E_0^{\neq}}<<1 \quad (3)$$

Hence the series $\sum_{n=0}^{\infty}\frac{(-1)^n \beta e^{[\beta-(n+1)\alpha]\Delta E_0^{\neq}}}{(\mathrm{n}+1)\alpha-\beta}$ converges.

For the other two series the ratio test cannot be applied since $\lim_{n\to\infty}\left|{u_{n+1}}/{u_n}\right|=1$. In this case we shall use the alternating series test[1-2] : suppose that in the series $\sum u_n$ (i) the terms are alternately positive and negative, (ii) $|\mathrm{u}_{\mathrm{n+1}}|<|u_n|$ and (iii) $\lim_{n\to\infty} u_n=0$. Then the series $\sum u_n$ converges. The first series of equation (2) gives,

$$|\mathrm{u}_{\mathrm{n+1}}|=\left|\frac{(-1)^{n+1}\beta}{(n+2)\alpha-\beta}\right|=\left|\frac{(-1)^{n}\beta}{(n+2)\alpha-\beta}\right|$$

Since $|(n+2)\alpha-\beta|>|(n+1)\alpha-\beta|$ it follows that $\left|\frac{1}{(n+2)\alpha-\beta}\right|<\left|\frac{1}{(n+1)\alpha-\beta}\right|$, and therefore $|\mathrm{u}_{\mathrm{n+1}}|<|u_n|$.

Taking the limit of the absolute value of the nth term gives,

$$\lim_{n\to\infty}|u_n|=\lim_{n\to\infty}\left|\frac{(-1)^{n}\beta}{(n+1)\alpha-\beta}\right|=\beta\cdot\lim_{n\to\infty}\left|\frac{1/n}{(1+\frac{1}{n})\alpha-\frac{\beta}{n}}\right|=0$$

Consequently, the given series converges. By a similar argument it can be shown that the second series is also convergent. This completes the proof.

The sums of each series in equation (2) were computed at www.wolframalpha.com and are given below,

$$\sum_{n=0}^{\infty}\frac{(-1)^n\beta}{(n+1)\alpha-\beta}=0.56123,$$

$$\sum_{n=0}^{\infty}\frac{(-1)^n\beta}{n\alpha+\beta}=0.80692 \text{ and}$$

$$\sum_{n=0}^{\infty}\frac{(-1)^n \beta e^{[\beta-(n+1)\alpha]\Delta E_0^{\neq}}}{(\mathrm{n}+1)\alpha-\beta}=0.0005266$$

Finally, $\kappa(T)$ = 0.56123 + 0.80692 − 0.0005266 = 1.3676234 at T = 298.15 K.

References for session 8

1. J. A. Green, Sequences and Series, Routledge and Kegan Paul Ltd (1975)
2. M. H. Porter, C. B. Morrey, A first Course in Real Analysis, Springer-Verlag (1977)
3. Hoffmann, R.; Schleyer, P. von R.; Shaefer, H. F. III, Angew. Chem., Int. Ed. 2008, **47,** 7164
